# Discovery of NLX-266, an Orally Available and Metabolically Stable ERK1/2-Biased 5-HT_1A_R Agonist with Superior Antidepressant and Antiparkinsonian Activity

**DOI:** 10.1021/acs.jmedchem.5c00484

**Published:** 2025-04-23

**Authors:** Joanna Sniecikowska, Monika Gluch-Lutwin, Adam Bucki, Beata Gryzlo, Krzysztof Wieckowski, Justyna Godyn, Anna Wieckowska, Agata Siwek, Magdalena Jastrzebska-Wiesek, Anna Partyka, Agnieszka Cios, Anna Wesolowska, Adrian Newman-Tancredi, Marcin Kolaczkowski

**Affiliations:** † Jagiellonian University Medical College, 9 Medyczna Street, Kraków 30-688, Poland; ‡ Neurolixis SAS, Labruguière, Castres 81290, France

## Abstract

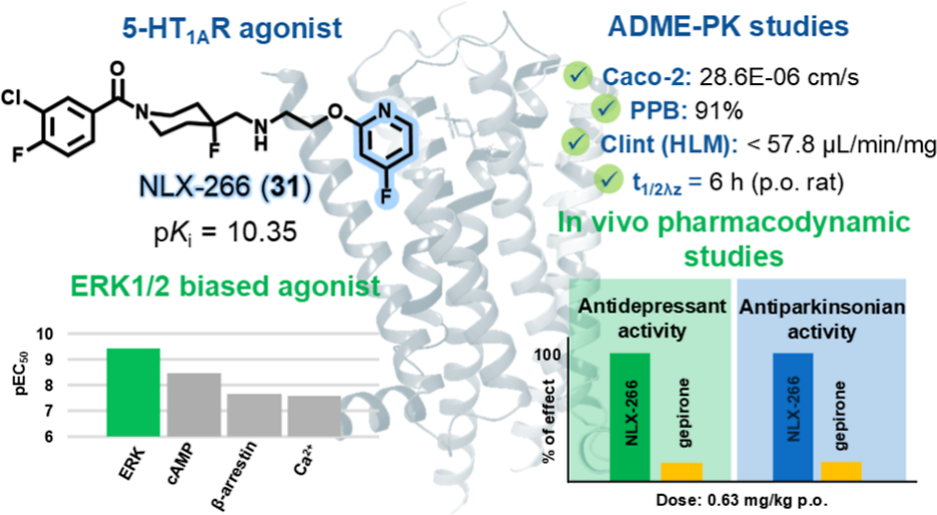

We report the discovery of NLX-266 (**31**), an orally available and metabolically stable ERK1/2-biased 5-HT_1A_ receptor agonist, which demonstrates both enhanced antidepressant and antiparkinsonian-like activities. A new series of 1-(1-benzoylpiperidin-4-yl)methanamine derivatives were synthesized and screened for their affinity and selectivity toward the 5-HT_1A_ receptor. Notably, **31** exhibited exceptional binding affinity (p*K*_i_ > 10) and selectivity (>1000×) over the adrenergic α_1_ and dopaminergic D_2_ receptors. In vitro functional assays revealed that **31** preferentially activates ERK1/2 phosphorylation, correlating with significant antidepressant effects in the forced swim test in rats at low doses (MED = 0.63 mg/kg p.o.). Furthermore, **31** demonstrated potent antiparkinsonian effects by reversing haloperidol-induced catalepsy at very low doses (MED = 0.04 mg/kg p.o.). The pharmacokinetic profile of **31** indicates favorable exposure and a prolonged half-life following oral administration. These findings suggest that **31** is a promising candidate for future exploration aiming at treatment of depression and/or Parkinson’s disease.

## 1 Introduction

Among the 14 subtypes of serotonin receptors, the 5HT_1A_ receptor subtype was the first to be identified, cloned, and sequenced.^1,2^ In fact, 5-HT_1A_ receptors have a central role in the control of serotonergic activity due to their involvement in the regulation of many physiological functions, such as mood, emotions, circadian rhythm, and pain conduction.^3^ The double neuronal localization of 5-HT_1A_ receptors, both postsynaptically as heteroreceptors in various brain regions (e.g., cerebral cortex, hippocampus, or hypothalamus) and presynaptically as autoreceptors on the bodies of serotonin neurons in the raphe nuclei, determines their potential therapeutic benefit in the treatment of various neuropsychiatric and neurological disorders, such as anxiety disorders, depression, schizophrenia, Parkinson’s disease, or pain.

However, the diverse localization of 5-HT_1A_ receptors, along with their involvement in various functions and sometimes opposing effects (pre-vs postsynaptic), presents a significant therapeutic challenge. This complication could potentially be addressed by developing more precise pharmacological tools that selectively activate specific subpopulations of these receptors. For years, the above-mentioned complexity has been a substantial obstacle in the development of drugs that are selective, full agonists of the 5-HT_1A_ receptors, with high therapeutic effectiveness and limited side effects. While 5-HT_1A_ receptor agonism, especially partial agonism, is an element of the pharmacological profile of various approved drugs, none of them is a fully selective, full agonist of the 5-HT_1A_ receptor. Numerous ligands, of diversified and interesting structures, have so far been developed aiming at preferential activation of 5-HT_1A_ receptors.^4^ However, many of them suffered from serious limitations such as insufficient (or only partially explored) selectivity, only partial agonism, or low (or unknown) metabolic stability. These features made them not suitable for precision pharmacology and did not allow the full realization of the therapeutic potential of targeting 5-HT_1A_ receptors.^3^

**Figure 1:**
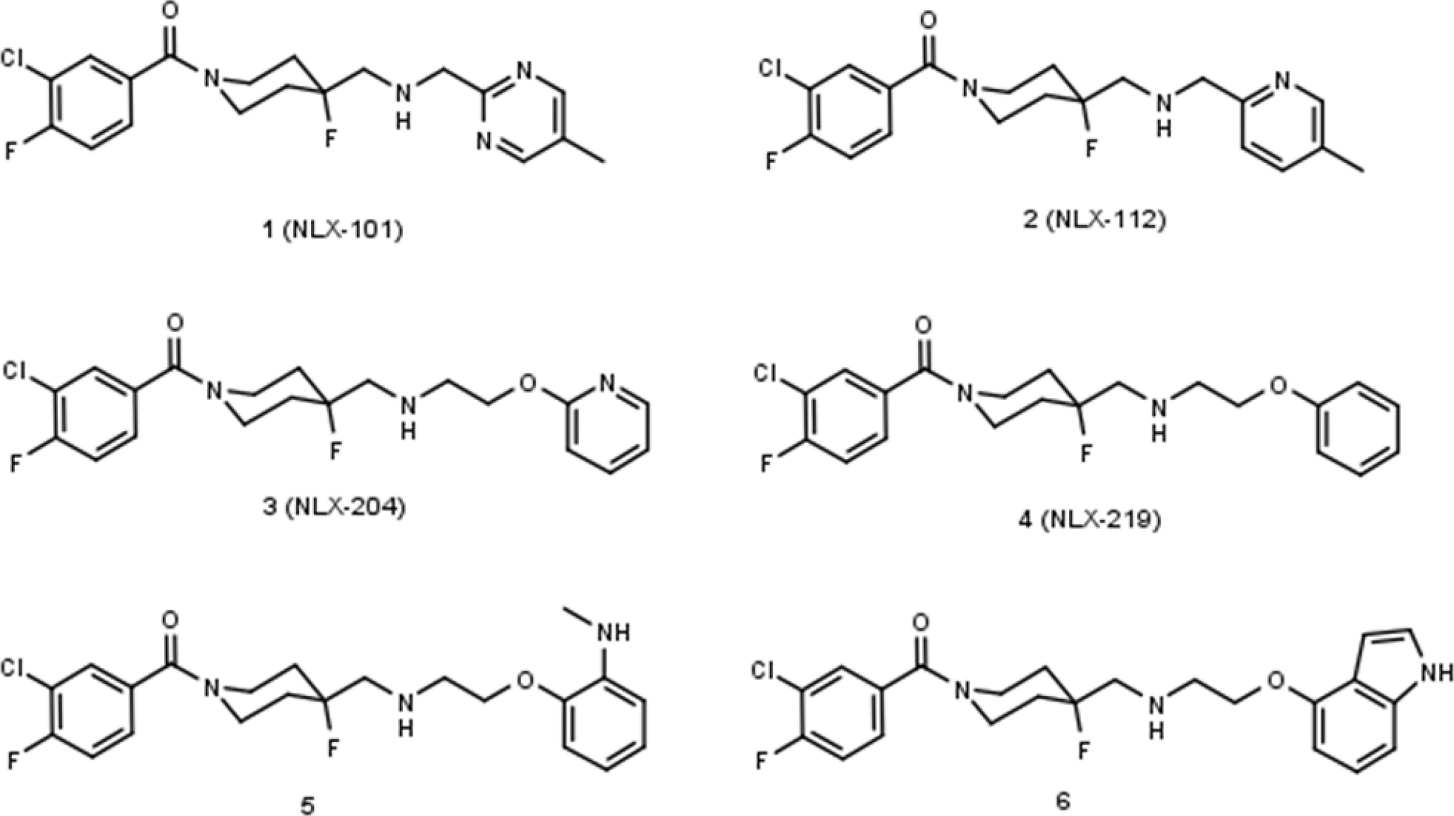
Functionally selective 5-HT_1A_R agonists based on 1-(1-benzoylpiperidin-4-yl)methanamine structure.

A major advance in this respect was the development of a series of 1-(1-benzoylpiperidin-4-yl)methanamine derivatives, which were characterized by both high selectivity and full agonism at 5-HT_1A_ receptors.^4,5^ Among the derivatives of this group is the compound NLX-101 (aka F15599, **1**), the first “biased” agonist at postsynaptic 5-HT_1A_ receptors, which exhibits preferential activation of cortical brain regions in vivo, high antidepressant activity, and low side effects liability (Figure 1).^6−10^ Moreover, another compound from this series, NLX-112 (aka F13640, befiradol, **2**), was characterized by pronounced analgesic activity and also developed as an ^18^F-labeled ligand with unique properties as the first 5-HT_1A_ full agonist PET radiotracer for brain imaging (Figure 1).^11−14^ In preclinical studies, **2** showed exceptional efficacy in models of Parkinson’s disease, and in a recent phase IIa clinical trial, it alleviated both levodopa-induced dyskinesias and motor disability in patients with Parkinson’s disease.^15−21^ This is the first time such dual therapeutic efficacy in Parkinson’s disease has been reported for a compound with serotonergic activity, suggesting a high therapeutic potential for **2** and new selective full agonists at 5-HT_1A_ receptors.

Recently, we discovered and patented a new group of 1-(1-benzoylpiperidin-4-yl)methanamine derivatives, containing aryloxyethylene moieties (e.g., Figure 1, compounds **3–6**).^22−24^ This new series is characterized by a better match to the 5-HT_1A_ receptor binding site, which resulted in compounds with even higher affinities while maintaining a high selectivity and a high level of 5-HT_1A_ receptor agonism. Moreover, the new series is also characterized by high synthetic flexibility, which allowed the synthesis of numerous derivatives.^24^ These have been structurally diversified, mainly in the aryloxy fragment, because this moiety is accommodated in the part of the 5-HT_1A_ receptor binding site which is crucial for receptor activation.^25^ These studies led to the identification of compounds with diverse functional profiles in four cellular functional assays (cAMP inhibition, pERK1/2 activation, Ca^2+^ mobilization, and β-arrestin recruitment).^22,23^

Following the discovery of the functional selectivity of NLX-101, which was found to preferentially activate ERK1/2 phosphorylation,^26^ we proved with the new series that it is possible to achieve systematic changes in functional selectivity, by rationally designing the series of potentially biased agonists. Accordingly, novel biased agonists were discovered that show functional selectivity for ERK1/2 phosphorylation or for β-arrestin recruitment (Figure 1, compounds **5** and **6**, respectively). Importantly, such distinct biased agonism translated to differences in their in vivo efficacy vs side effects profile.^23^ Noteworthy, the unsubstituted pyridine-2-yl oxyethyl derivative, **3** (NLX-204) (Figure 1, compound **3**), was found to have very promising developability properties and also a robust antidepressant effect in both the rat forced swimming test and the Chronic Mild Stress model, which is considered a golden standard for preclinical antidepressant activity.^8,9^ The antidepressant-like effects of **3** were rapid and sustained, occurring at low doses and achievable after oral administration, suggesting superior therapeutic activity compared with currently available reference antidepressants.^8,9,22^ The antidepressant potential of the selective 5-HT_1A_ receptor activation was corroborated by the recent Nature study showing that a 5-HT_1A_-selective analogue of a psychedelic 5-MeO-DMT is devoid of hallucinogenic-like effects while retaining anxiolytic-like and antidepressant-like activity in socially defeated animals.^27^

In our previous studies, we investigated the influence of the linker structure as well as the number and position of nitrogen atoms in the distal aryl moiety, yielding the two lead structures, namely the pyridin-2-yloxy- and phenoxy-ethyl derivatives of benzoylpiperidynemethanamine.^22^ We also explored the substitution pattern at the phenoxy moiety, to obtain numerous derivatives of **4** (Figure 1, compounds **4**–**6**).^23^ Therefore, in the present study, we decided to also investigate the effects of modifications of the pyridin-2-yloxy derivatives. Here, we present the synthesis, radioligand binding affinity, target selectivity studies, and broad functional characteristics of the variously substituted derivatives of lead compound **3** (Figure 1). The most promising new compounds were also studied for in vitro ADME properties, as well as early exploratory PK and PD studies, focusing on potential antidepressant and antiparkinsonian activity. This resulted in the discovery of NLX-266 (**31**), an ERK-1/2 biased 5-HT_1A_ receptor selective agonist with superior antidepressant and antiparkinsonian-like effects.

## 2 Results and Discussion

### 2.1 Synthesis

**Scheme 1:**
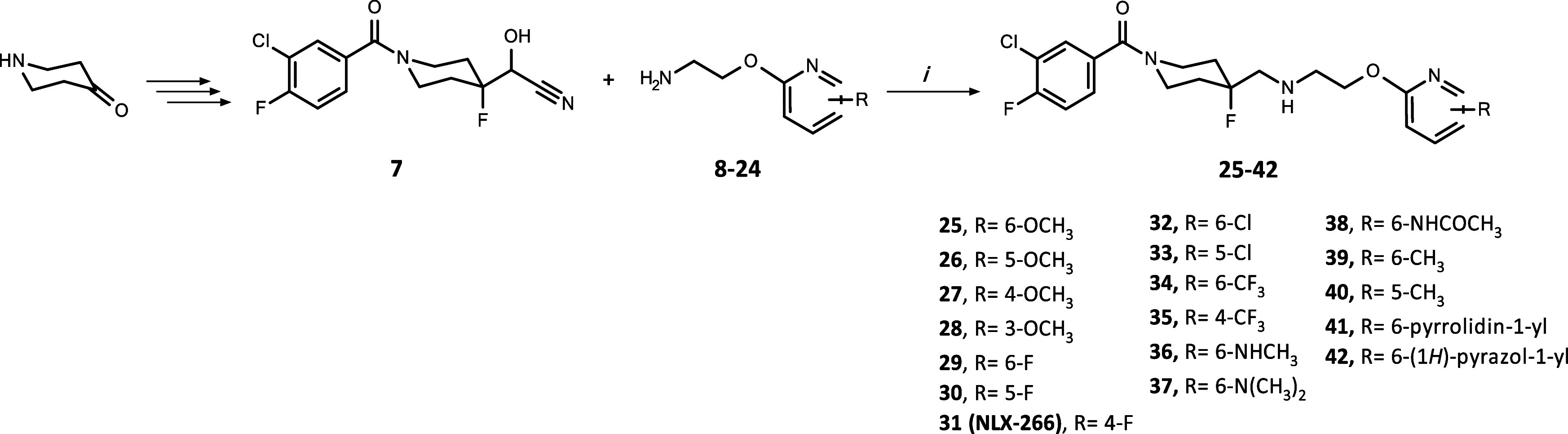
Synthesis of the Final Compounds **25–42** Reagents and conditions: (i) NaCNBH_3_, DABCO, molecular sieves, FeSO_4_·7H_2_O, MeOH, rt, 36–72 h, yield: 17–96%.

Target compounds **25**–**42** were prepared in the four-step synthesis that we have previously reported.^22,28^ Starting with the acylation of commercially available 4-piperidone with 3-chloro-4-fluorobenzoyl chloride followed by the Darzens reaction, we obtained cyanoepoxide, which was then converted into cyanohydrin **7** through a regioselective ring–opening reaction using Olah’s reagent. The last step of synthesis was a reductive amination (Scheme 1) between cyanohydrin **7** and appropriate pyridin-2-yl oxyethanamines **8**–**24**. This reaction was carried out using sodium cyanoborohydride as the reducing agent, DABCO (1,4-diazabicyclo[2.2.2]octane) as the base, and iron sulfate heptahydrate to complex cyanide ions.

**Scheme 2:**
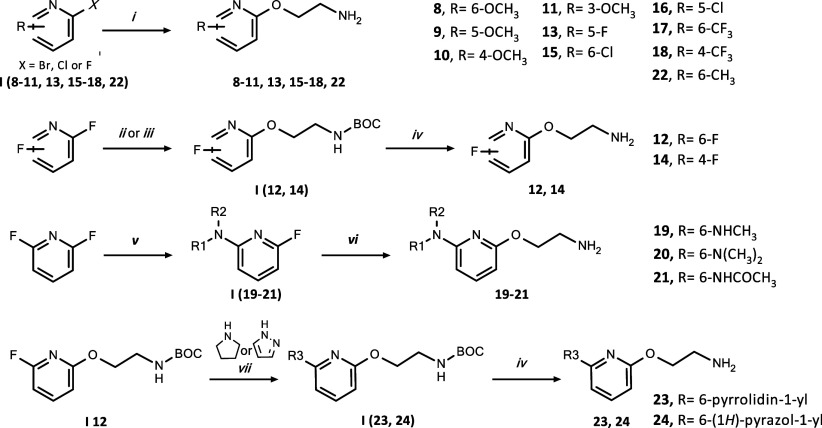
Synthesis of the Pyridin-2-yloxyethanamines **8–24** Reagents and conditions: (i): 2-aminoethanol, NaH, 1,4-dioxane, r.t. or 80 °C, 24–48 h.; (ii) *tert*-butyl-2-hydroxyethyl carbamate, NaH, THF, 0 °C then r.t., 3 h; (iii) *tert*-butyl-2-hydroxyethyl carbamate, NaH, 1,4-dioxane, microwave, 90 °C, 20 min; (iv) 1.0 M HCl in EtOAc, r.t., 24 h; (v) 2.0 M methylamine or dimethylamine in THF, microwave, 150 °C, 20 min, or acetamide, NaH, THF, MW, 100 °C, 20 min; (vi) 2-aminoethanol, NaH, 1,4-dioxane, microwave, 90 °C, 20 min; (vii) pyrrolidine, 80 °C, 3 h then r.t., 48 h, or pyrazole, NaH, DMF, 80 °C, 3 h.

Differently substituted pyridin-2-yl oxyethanamines **8–24** were prepared in the one- to three-step procedures, starting with nucleophilic aromatic substitution of pyridine halides (Scheme 2). Amines **8–11**, **13**, **15–18**, and **22** were synthesized by reaction of commercially available 2-pyridine halides **I** (**8–11**, **13**, **15–18**, and **22**) with 2-aminoethanol. Amines **12** and **14** were obtained by substitution of 2,6-difluoropyridine and 2,4-difluoropyridine, respectively, with *tert*-butyl-2-hydroxyethyl carbamate followed by Boc-deprotection. Compound **I 12** was also converted to pyrrolidine and pyrazole derivatives **I** (**23**, **24**), which after Boc-deprotection, led to amines **23** and **24**. Amines **I 19–21** were obtained by treating 2,6-difluoropyridine with methylamine (**I 19**), dimethylamine (**I 20**), and acetamide (**I 21**).^29^ Subsequent substitution of the fluorine atom in **I** (**19–21**) with 2-aminoethanol led to amines **19–21**. A detailed description of the preparation of amines **8–24** is provided in the Supporting Information.

### 2.2 Structure Activity Relationships

All of the final compounds obtained were analyzed for their radioligand inding affinity. In view of previous findings, the main focus was on the role of the substituents at the pyridine ring. Various types of substituents and their positions at the pyridine core were explored (Table 1). The compounds **27** and **31**, being the C-4 substituted analogs (methoxy- and fluoro-, respectively), showed the highest affinity, comparable to that of the unsubstituted compound **3**. On the other hand, compound **35** with trifluoromethyl substituent at C-4 position was significantly weaker, but it should be noted that this substituent also decreased affinity in the series of phenoxyethanamine derivatives,^23^ suggesting that it is generally unfavorable for aryloxyethyl derivatives of 1-(1-benzoylpiperidin-4-yl)methanamine. Very high affinity was observed for compounds **25**, **36** and **37**, being the C-6 substituted methoxy-, methylamino-, and dimethylamino-derivatives. In contrast, a decrease in affinity was observed for other derivatives substituted at the C-6 position, ranging from about 0.5 to 1.5 order of magnitude (in relation to **3**). Derivatives with substituent in C-5 position of pyridine (**26**, **30**, **33**, Table 1), similarly to *para* substituted phenoxy-derivatives,^23^ all reduced 5HT_1A_ receptor affinity (expressed as p*K*_i_) by 0.27 to 1.98 log units, the least unfavorable being the least bulky, 5-fluoro analog **30** (p*K*_i_ 9.92).

**Table 1:**
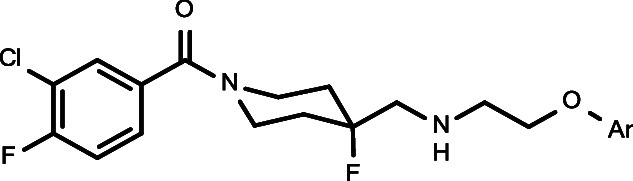

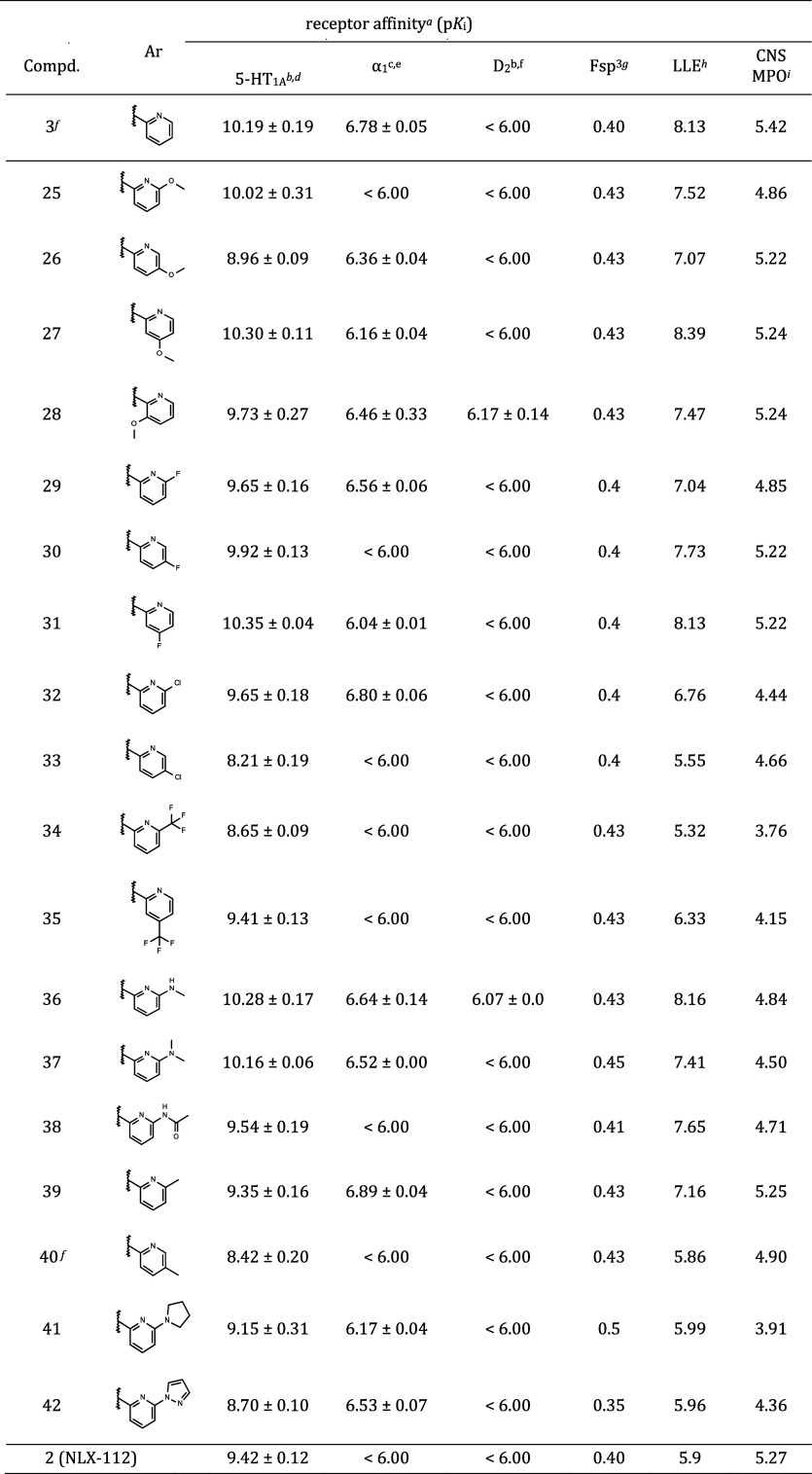
5-HT_1A_ Receptor Affinity, Selectivity vs Main Off-Targets, and Developability Parameters of the Final Compounds

a All binding affinity values were expressed as p*K*_i_, calculated as—log *K*_i_, and presented as mean ± SEM from at least three independent experiments conducted in duplicate.

b 5-HT_1A_ and D_2_ radioligand binding assays were performed using CHO-K1 cells.

c α_1_ radioligand binding experiment was conducted with rat cortex tissue.

d Affinity was measured using [^3^H]8-OH-DPAT.

e Affinity was measured using [^3^H]-prazosin, with phentolamine showing a p*K*_i_ of 7.95.

f Aaffinity was measured using [^3^H]-methylspiperone, where haloperidol exhibited a p*K*_i_ of 8.85.

g Fraction of sp^3^ carbon atoms.

h Ligand-lipophilicity efficiency referring to the 5HT_1A_R.

i Central nervous system multiparameter optimization score.

j Data for compounds **3** and **40** are reproduced from the previous paper.^22^

Worth emphasizing is the fact that the derivatives presented herein showed, in the worst case, single digit nanomolar binding affinity (when expressed as *K*_i_) and most of them reached subnanomolar values. In head-to-head comparison in almost all cases, the 5HT_1A_ receptor binding affinity of the pyridin-2-yl oxyethanamine derivatives (**25**, **28**, **29, 31–34**, **35**, **36**, **38**, and **39**) was relatively lower than their phenoxyethanamine counterparts,^23^ however, the differences were not pronounced. This data contrasts with results presented for pyridinemethanamine derivatives previously reported by Vacher et al., where the pyridine derivatives showed higher affinity than their benzene counterparts.^30^ It should be noted, however, that those works concerned a different chemotype, and our molecular modeling analyses indicate that the role of the electronegative pyridine nitrogen atom of the derivatives studied by Vacher et al. could be taken over by the oxygen atom of the ethoxy linker in the novel series.^22^

### 2.3 Selectivity vs Main Off-Targets (α_1_R and D_2_R)

The tested compounds showed very low affinity for dopamine D_2_ receptors, which reached p*K*_i_ values slightly above 6 only in the case of two compounds, **28** and **36**, the derivatives of 3-methoxy- and 6-methylamino-2-pyridine, respectively. In the light of the very high affinity of all these compounds for the 5-HT_1A_ receptor, their D_2_ receptor affinity can be considered comparatively negligible (>1000× selectivity). The tested compounds exhibited slightly greater affinity for the α_1_ adrenergic receptor compared to the D_2_ receptor, however, still much lower than for the main biological target, the 5-HT_1A_ receptor, resulting in overall high selectivity. The majority of the compounds exhibited strong selectivity for 5-HT_1A_ receptors, >1000-fold, and it was slightly lower only in the case of 6 compounds (**26**, **28**, **32**, **39**, **41**, **42**). Compound **32**, a 6-chloro-2-pyridine derivative, was characterized by the highest affinity for the α_1_ receptor.

### 2.4 Structure Functional Relationships

In order to investigate the in vitro functional profiles, all final compounds were evaluated using four functional assays: stimulation of ERK1/2 phosphorylation (pERK1/2), adenylyl cyclase inhibition (cAMP), β-arrestin recruitment (β-arrestin), and calcium mobilization (Ca^2+^) (Table 2). In accordance with our previous studies on 5-HT_1A_ receptor agonists, agonist efficacy (*E*_max_) was ranked as follows: *E*_max_ exceeding 80% of serotonin’s maximal effect was classified as indicative of a full agonist, while *E*_max_ values ranging from 79% to 21% were considered characteristic of partial agonism, and *E*_max_ values of 20% or below were considered to indicate antagonism.

**Table 2:**
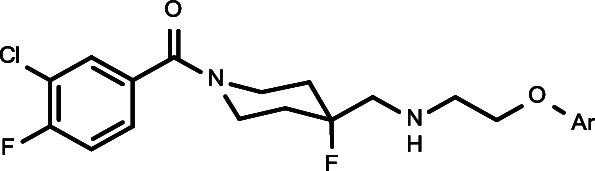

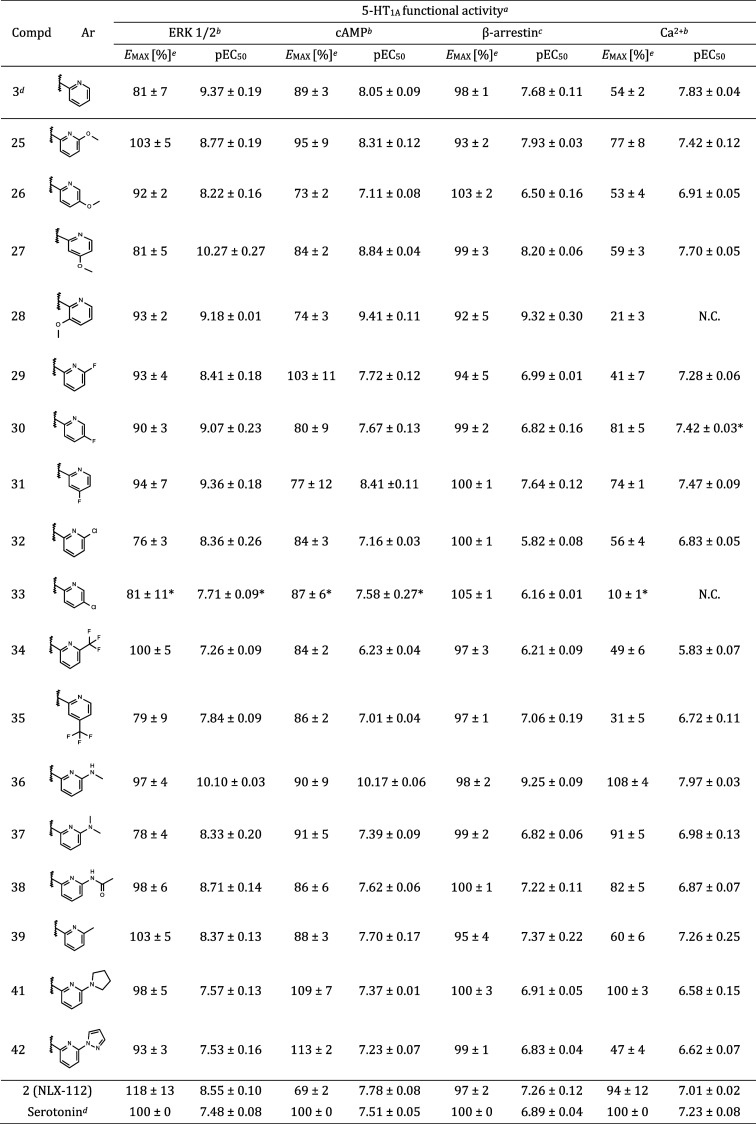
Functional Activity Results at 5-HT_1A_R

a All functional activity values are presented as means from a minimum of three experiments conducted in duplicate, unless stated otherwise. The functional assay was carried out using ^b^CHO-K1 cells and ^c^U2OS cells (Tango LiveBLAzer assay kit).

d Data for serotonin and compd. **3** on ERK, cAMP, and β-arrestin were taken from the previous paper.^22^ NC—not calculable; * values are reported as mean ± range from two experiments performed in duplicate.

e *E*_MAX_ values represent the ligand response, expressed as a percentage of the maximum effect induced by serotonin at 1.0 × 10^–5^ M.

Comparing the new derivatives with their parent structure **3**, the introduction of a substituent to the pyridine ring generally decreased potency. Only the substitution of the methylamine moiety in position 6 (**36**) or the methoxy substituent in position 4 (**27**) of the pyridine system allowed maintaining or increasing the activity in all signal transduction pathways. In addition, it was observed that the methoxy substituent in this series of derivatives was the best tolerated substituent in the cAMP and β-arrestin assays, because out of the 4 compounds tested, only the 5-methoxy derivative (**26**) was characterized by a decrease in activity relative to **3**. The low activity of **26** in relation to its isomers (**25**, **27**, and **28**) is probably due to the fact that position 5 in the pyridine system, similar to the *para* position in **4** derivatives, is not a privileged position, as the substituent in this place constitutes too much a steric hindrance in the receptor, leading to a decrease in activity.^23^ As with the previously published phenoxyethanamine derivatives, almost all new pyridin-2-yl oxyethanamine derivatives were full agonists in the pERK1/2, cAMP, and β-arrestin assays. The greatest variation in *E*_max_ values was again observed for the calcium mobilization pathway: only one compound **33** was characterized by a marginal level of activation (*E*_max_ < 20%), 5 compounds (**30**, **36**–**38**, and **41**) showed full agonism, and the remaining structures were classified as partial agonists in this assay.

In order to assess the relative preference of pathway activation and functional selectivity of the novel compounds, bias factors were calculated according to the methodology used previously (Table 3).^22,23^ Serotonin was used as a reference “unbiased” agonist. The analysis of bias factors showed that as in the previous series, most compounds tended to preferentially activate the ERK1/2 kinase phosphorylation pathway. This tendency was the strongest in the case of the pERK—Ca^2+^ mobilization relationship, which results from a weak activation of the latter pathway. When ERK phosphorylation was compared to the effect on cAMP levels and β-arrestin recruitment, the trend for ERK preference was lower but also clear. The highest bias factor for ERK phosphorylation, in relation to β-arrestin (above 1), was found for compounds **26**, **27**, **30**, **3**, and **32**. In relation to cAMP, a bias factor above 1 was also found for compounds **34** and **38**. Comparing the activation of the cAMP and β-arrestin pathway, it was observed that neither compound showed a significant bias for either of these pathways, however, most of the compounds showed a tendency to a relatively stronger recruitment of β-arrestin. Also, none of the tested compounds showed any tendency to a stronger activation of the calcium mobilization pathway over the ERK1/2 and cAMP pathways. Only two derivatives (**32** and **30**) showed a slight tendency toward the activation of calcium mobilization relative to the recruitment of β-arrestin.

**Table 3:**
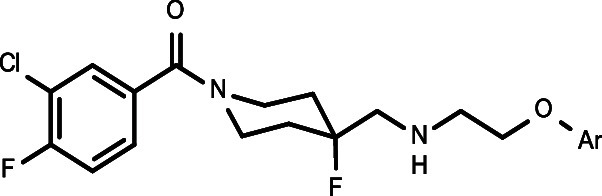

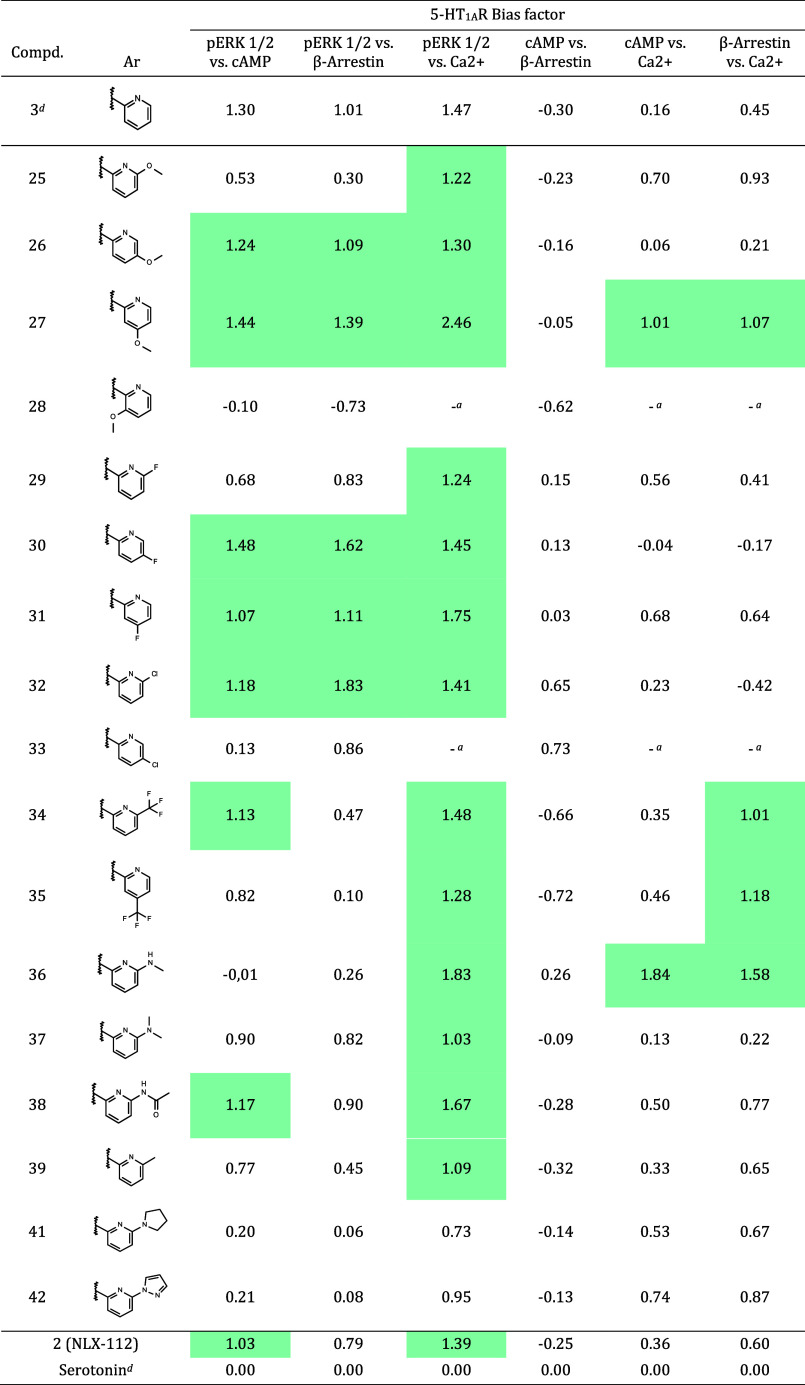
Bias Factors at 5-HT_1A_R

a - no bias result due to lack of data for Ca^2+^. Compounds showing significant bias (over 1) are marked in green (positive values).

b Data for serotonin and compound **3** on ERK, cAMP, and β-arrestin were taken from the previous paper.^22^

**Figure 2:**
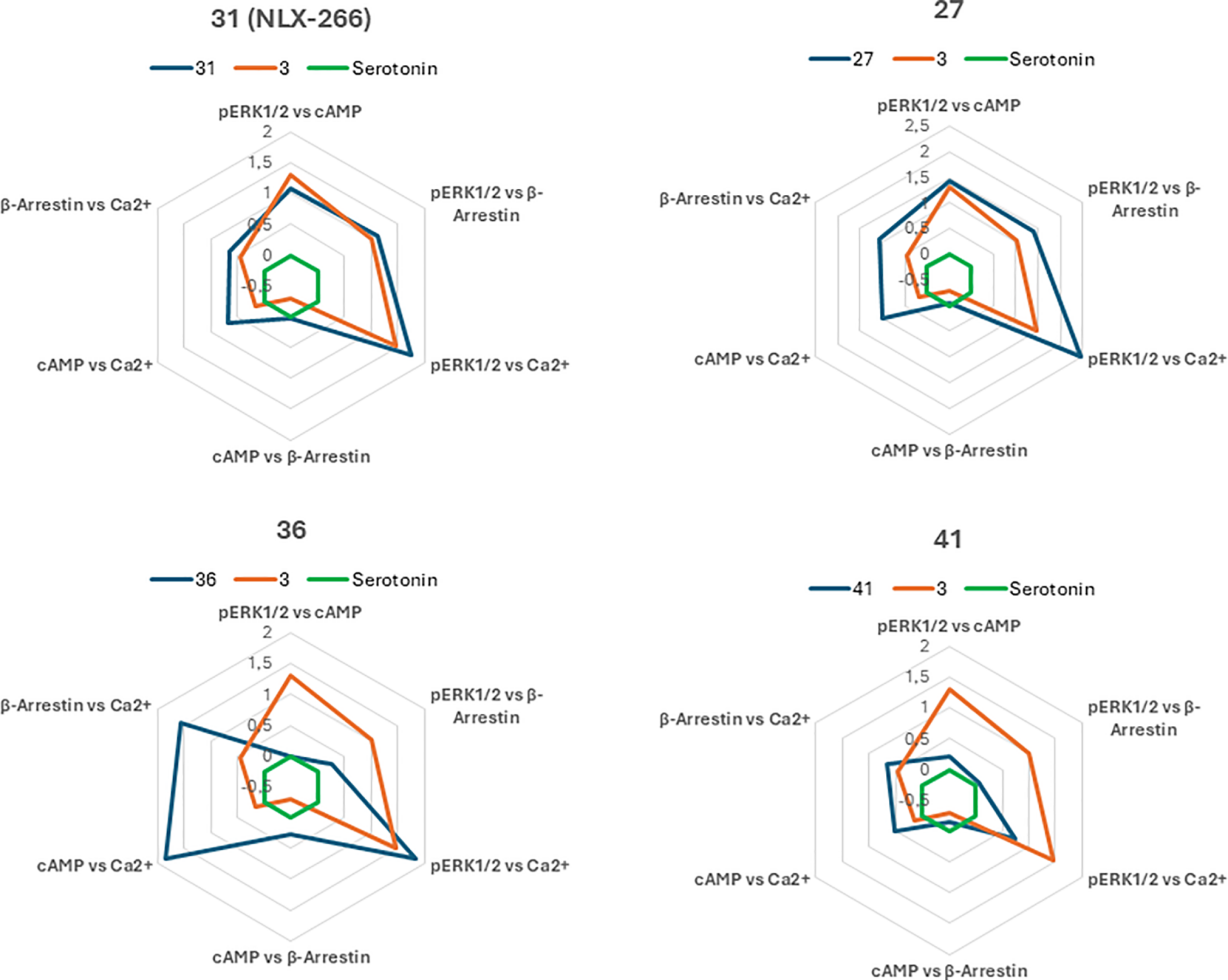
Radar charts of the bias factors for selected compounds representing different signaling profiles. The bias factors plots of novel compounds (dark blue) were superimposed on the graph for the starting compound **3** (NLX-204) (orange) and for serotonin (green), as a natural reference ligand (unbiased).

It is worth noting that despite the general tendency toward stronger activation of ERK1/2 phosphorylation, the new series included compounds with diverse functional profiles. Among others, also compounds that do not show a preference for pERK1/2 vs cAMP or β-arrestin (but only vs Ca^2+^, e.g., compound **36**), as well as those that do not show a clear preference for any of the signaling pathways (all bias factors <1, e.g., compound **41**) were found. This was additionally visualized using radar charts (Figure 2). Thus, it can be seen that different substituents at the pyridine ring have different effects on the functional profile. These differences are less pronounced than in the previously described series of phenoxyethanamine derivatives but still present.

As for possible interactions that affect the general tendency of this series to prefer ERK1/2 phosphorylation and thus relatively reduce the ability to diversify the functional profile in this series, a possible explanation is the presence of a nitrogen atom in the 2-position of the pyridine moiety in all compounds. The presence of an HBA in this place, as a substituent or heteroatom, was identified by us in the previous work, within the structure–functional relationships, as limiting the tendency to activate β-arrestin and thus relatively emphasizing the preference for ERK1/2 phosphorylation.

Based on the results discussed above, the compounds characterized by the highest bias for ERK1/2 phosphorylation were considered for further studies. Taking into account the presence of a potentially reactive fragment in the structure of **32** (6-chloropyridine) and the relatively lower affinity and potency of **26** and **30**, compounds **27** and **31** were selected for preliminary in vitro ADME tests.

### 2.5 In Vitro ADME and In Vivo PK Studies

Both **27** and **31** showed favorable preliminary in vitro ADME properties (Table 4). The Caco-2 permeability in both the A-B and B-A ways was high (>10^–5^ cm/s) and comparable to the positive control, propranolol (24.4 × 10^–6^ cm/s). No significant differences in B-A vs A-B permeability were noted, indicating no risk of efflux. Plasma protein binding was relatively high but not extreme (90–95%) and comparable to that of compound **3**, which was proven to show high in vivo activity. Both compounds also showed promising metabolic stability on rat liver microsomes resulting in *T*_0,5_ of 62.2 and 89.9 min for **27** and **31**, respectively. Considering the above, both compounds underwent preliminary in vivo PK studies in rat.

**Table 4:** In Vitro ADME Data and Physiochemical Properties for Compounds **27** and **31**

assay		compound **27** (10 μM^a^)	compound **31** (10 μM^a^)
permeability (Caco-2)^b^^31^	A–B [10^–6^ cm/s]	30.2	28.6
	B–A [10^–6^ cm/s]	20.4	13.8
Efflux ratio^c^		0.68	0.45
metabolic stability (RLM)^32^	intrinsic clearance (CL_int_) [μL/min/mg]	111.6	77.9
	half-Life (*T*_1/2_) [min]	62.2	89.9
metabolic stability (HLM)^32^	intrinsic clearance (CL_int_) [μL/min/mg]	263.6	<57.8
	half-Life (*T*_1/2_) [min]	26.5	>120
protein binding^d^^33^		93%	91%
p*K*_a_		7.24	7.29
Log *P*		3.22	3.14
Solubility^34^	intrinsic aqueous solubility (log *S*_0_)	–3.23	–3.24

a Metabolic stability study was conducted at the concentration of 0.1 μM.

b Caco-2 at pH 6.5/7.4.

c The efflux ratio is expressed as (*P*_app_ B to A)/(*P*_app_ A to B).

d Human plasma.

Compounds **27** and **31** were administered orally (p.o.) to rats at a dose of 2.5 mg/kg (Table 5). Both were characterized by relatively quick absorption after oral administration and rapid penetration into the brain, which was reflected in the low values of the time to reach the maximum concentration (*T*_max_). In the case of **31**, *T*_max_ was 15 min for both serum and brain, while in the case of **27**, these values varied, being 5 min for serum and 30 min for brain. The maximum concentration (*C*_max_) of **27** was twice as high as that of **31** in the case of serum (100.24 vs 50.4 ng/mL), while in the case of the brain these values were almost equal (60.39 and 63.70 ng/g for **27** and **31**, respectively). The maximum total concentrations of compounds **27** and **31** in serum, expressed in molar concentration, are 227 and 118 nM, respectively, and taking into account the free fraction, 15.9 and 10.6 nM, respectively. This confirms that these concentrations cover the range of active concentrations determined in vitro, and thus support likely target engagement. **31** had a higher *V*_z_ volume of distribution (*V*_z_/F) during terminal phase, equaling 48.31 L/kg, compared to 34.77 L/kg for **27**, which may be related to its higher lipophilicity (*c* log *P* 2.95 vs 2.65). **31** also had a significantly longer half-life ( 367.57 min vs 96.65 min for **27**) and clearly (2–3 times) higher total exposure values (AUC_0–∞_). In the case of **31**, they reached 27441.21 and 26461.35 ng·min/mL for serum and brain, respectively, while for **27** they equaled 10024.70 and 13222.75 ng·min/mL, respectively. Taking into account the significantly higher total exposure and significantly longer half-life, as well as more uniform times of maximum exposure, **31** was considered to have a more favorable PK profile, and thus, it was selected for in vivo pharmacodynamics studies. It should be noted that these parameters of **31** are also superior to the lead compound **3**, which was characterized by significantly shorter half-life ( = 89.3 min) and lower exposure (AUC_0–∞_ = 8962.3 ng·min/L)^22^ Noteworthy, the favorable profile of **31** is further supported by its high metabolic stability determined using human liver microsomes (HLM, Table 4).

**Table 5:** In Vivo PK Results for Compounds **27** and **31** after Oral Administration of a Dose of 2.5 mg/kg to Rats^a^

PK data (2.5 mg/kg, p.o.)	**27**	**31**
*T*_max_ [min]	5	15
*T*_max_brain [min]	30	15
*C*_max_ [ng/mL]	100.24	50.4
*C*_max_brain [ng/g]	60.39	63.7
AUC_0-∞_ [ng·min/mL]	10024.7	27441.21
AUC_0-∞_brain [ng·min/g]	13222.75	26461.35
[min]	96.65	367.57
brain [min]	151.81	276.07
*V*_*z*_/*F* [L/kg]	34.77	48.31

a Pharmacokinetic parameters of compounds **27** and **31** in serum and brain after p.o. dosing of 2.5 mg/kg to rats were assessed using the noncompartmental approach (*n* = 5–6/group).

### 2.6 Antidepressant and Antiparkinsonian-like Activity in Rat

**Chart 1:**
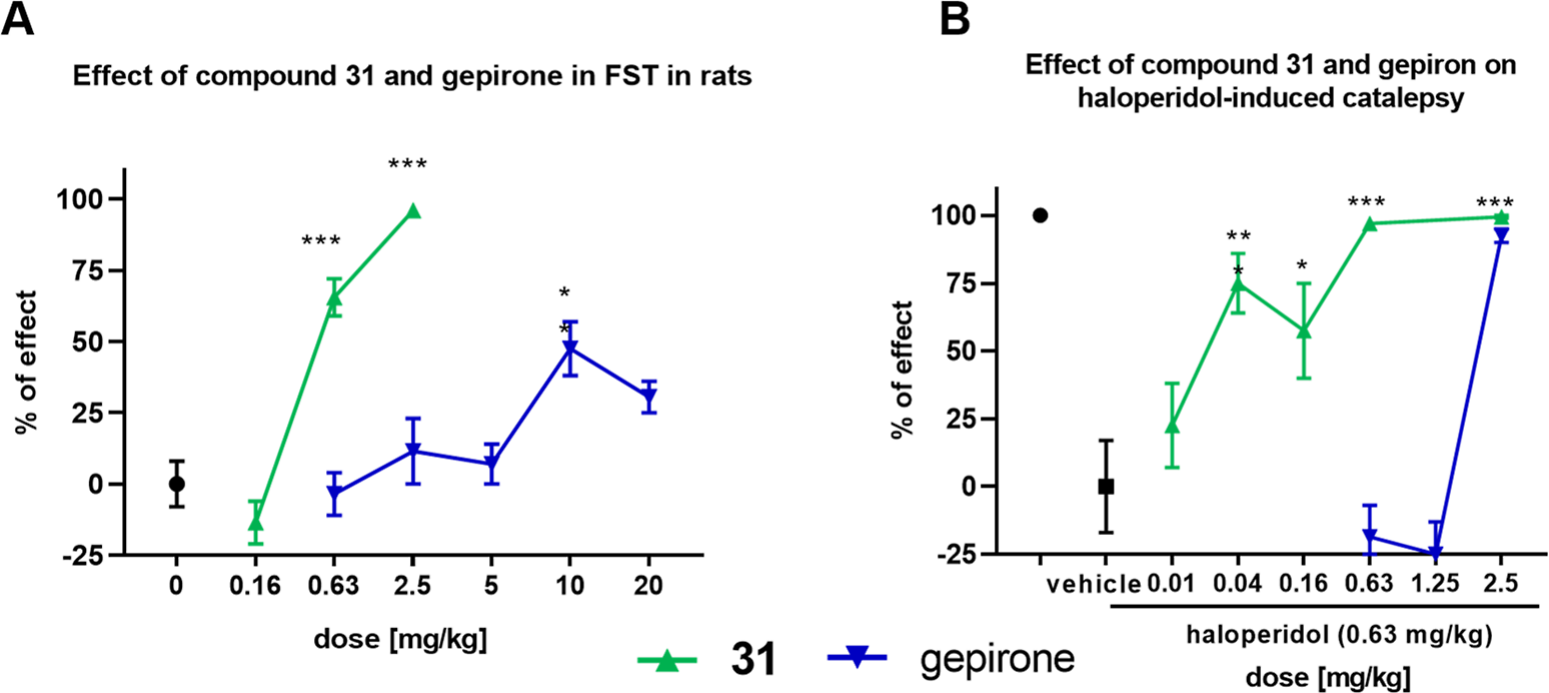
Antidepressant-like and antiparkinsonian-like activity of compound **31** (in green) and gepirone (in blue)a

The selected compound **31** was tested for antidepressant activity in the Porsolt forced swim test (FST) in rats. It showed robust activity, causing a significant reduction of immobility at a dose of 0.63 mg/kg po (Chart 1A). Most importantly, its activity was dose-dependent, and at a dose of 2.5 mg/kg p.o., an almost complete reduction of immobility was achieved, consistent with efficacious antidepressant-like activity. It is also worth emphasizing that the effect obtained was fully specific because it was reversed by the administration of a selective 5-HT_1A_R agonist, WAY-100635. Additionally, at the active dose, the compound did not increase locomotor activity (Tables S2 and S6). The antidepressant activity of **31** was similar to that of previously tested 1-(1-benzoylpiperidin-4-yl)methanamine derivatives, compounds NLX-101 (F15599) and **3**, which is mediated by activation of postsynaptic 5-HT_1A_ receptors in the cortex.^8,35^ It was also clearly higher than the activity of reference drugs: buspirone and gepirone.^36^ Indeed, buspirone was inactive in this test, while gepirone was effective only at a dose of 10 mg/kg po and did not achieve the maximum reduction of immobility (reached only about half of the maximum effect). Moreover, at a higher dose of 20 mg/kg, some of the anti-immobility effects of gepirone were lost (Chart 1A). In any case, the present data confirm the superior antidepressant-like activity of NLX-266 in this test over an antidepressant that was recently approved by the FDA.

a (**A**) Antidepressant-like activity in the FST in rats. **31** or gepirone was given po 60 min before the test. Results are expressed as mean ± SEM of the immobility time during the 5 min test session, compared to the respective vehicle group (one-way ANOVA followed by Bonferroni’s post hoc test); *N* = 7–8. (**B**) Antiparkinsonian-like activity of **31** and gepirone in the haloperidol-induced catalepsy (cross-leg position (CPL) model of catalepsy). **31** or gepirone was given p.o. while haloperidol s.c. 60 min before the test. Results are expressed as mean ± SEM of the crossed-legs position (CLP) compared to the respective vehicle group (one-way ANOVA followed by Bonferroni’s post hoc test); N = 7–8; *p < 0.05; **p < 0.01, ***p < 0.001; Detailed data available in Supporting Information Tables S1, S3–S5.

The potential antiparkinsonian-like activity of **31** was also assessed. For this purpose, it was tested for its capacity to reduce haloperidol-induced catalepsy in rats. **31** showed outstanding activity in this test, significantly reducing haloperidol-induced catalepsy (a model of parkinsonian rigidity)^37^ at a dose as low as 0.04 mg/kg p.o. and achieving almost total catalepsy reversal at a dose of 0.63 mg/kg p.o. (Chart 1B). The activity of **31** in this assay was comparable to that of **2**, which recently completed a successful Phase IIa clinical trial in patients with Parkinson’s disease.^21^ Similarly to the effects seen in the FST (see above), **31** outperformed both buspirone (which was inactive) and gepirone, which showed activity only at a dose of 2.5 mg/kg p.o (Chart 1B).

## 3 Conclusions

We designed and synthesized a series of novel 1-(1-benzoylpiperidin-4-yl)methanamine derivatives, analogs of recently reported lead compound NLX-204 (**3**), variously substituted at a pyridine-2-yloxyetyl moiety. These structural modifications aimed at potential diversification of functional activity and selectivity. All the obtained derivatives showed high affinity (p*K*_i_ > 8) for 5-HT_1A_ receptor as well as high selectivity over the most important off-targets, adrenergic α_1_ and dopaminergic D_2_ receptors (>1000× in most cases), confirming that decoration of the pyridine moiety allows to maintain those previously optimized properties. The novel series showed less diversity in functional profiles compared to the variously substituted phenoxyethyl derivatives,^23^ and a general trend favoring ERK1/2 phosphorylation was observed. Out of the compounds with the highest bias factor, the selected derivatives **27** (4-methoxy) and **31** (4-fluoro) were subjected to preliminary in vitro ADME and early PK studies to show favorable profiles. Compound **31** (NLX-266), was found to be more promising, due to a longer half-life and a higher total exposure after oral administration, comparing to both **27** and the lead compound **3**. In view of these favorable observations, **31** was selected for pharmacodynamic studies. It was found to exert full and dose-dependent activity in the Porsolt test in rats, which was reversed by the selective 5-HT_1A_ receptor antagonist and was not associated with changes in locomotor activity. Most importantly, **31** showed outstanding antiparkinsonian-like activity, being able to reverse haloperidol-induced catalepsy at a dose as low as 0.04 mg/kg p.o. These activities were clearly superior compared to the clinical drugs stimulating the 5-HT_1A_ receptor, including gepirone, recently approved as an antidepressant. Moreover, in view of the substantial comorbidity of depression with Parkinson’s disease,^38^ novel chemical entities that exhibit both antidepressant and antiparkinsonian activity could have considerable therapeutic interest. Overall, considering the above-mentioned favorable pharmacological properties and composition-of-matter patent protection, **31** is worth further investigation in more advanced models of antidepressant and antiparkinsonian activity as well as studies aiming to characterize its developability as a drug candidate.

## 4 Experimental Section

### 4.1 Chemistry

#### 4.1.1 General Chemistry Information

All procedures related to synthesis and analysis were performed according to the previously described methodology.^22,23^ Briefly, all the reagents were purchased from commercial suppliers. Purification was carried out via flash chromatography on silica gel columns (silica gel 60, particle size 40–63 or 20–40 μm). Preparative high-performance liquid chromatography (HPLC) was performed using Jasco LC-400 system with a Phenomenex Luna C8 column (5 μm, 15 mm × 21.2 mm). The purity (>95%) and molecular weight of all final compounds and the most important substrates was determined using UPLC/MS system (Waters ACQUITY UPLC (gradient elution: 5% to 95% acetonitrile/water + 0.1% v/v of formic acid, 0.3 mL/min, 10 min), UPLC BEH C18 column (1.7 μm, 2.1 × 100 mm, 40 °C), Waters eλ PDA detector (200–700 nm), Waters TQD mass spectrometer (ESI with tandem quadrupole). NMR spectra (^1^H, ^13^C, and ^19^F) were recorded on a JEOL 500 MHz or a Varian Mercury 300 MHz spectrometer. Marvin molecule editor was used for drawing, displaying, and characterizing chemical structures, substructures, and reactions (Marvin 21.15.11.53, 2021, ChemAxon, http://www.chemaxon.com).

#### 4.1.2 Synthetic Procedures

Compounds that were previously described in the literature or are commercially available: 2-(1-(3-Chloro-4-fluorobenzoyl)-4-fluoropiperidin-4-yl)-2-hydroxyacetonitrile (**7**),^22^ (3-Chloro-4-fluorophenyl)(4-(((2-((5-methylpyridin-2-yl)oxy)ethyl)amino)methyl)-4-fluoropiperidin-1-yl)methanone (**40**).^22^

##### 4.1.2.1 General Procedures for the Preparation of Pyridine-2-yloxy Derivatives of 1-(1-Benzoylpiperidin-4-yl)methanamine Derivatives (**25–42**)

The cyanohydrin **7**(22) (1.0 equiv), DABCO (2.0–12.6 equiv), and the appropriate amine (**8–24**) (1.0–1.6 equiv) were subsequently dissolved in methanol. Then sodium cyanoborohydride (1.6 or 7.8 equiv), iron sulfate heptahydrate (FeSO_4_×7H_2_O) (1.1 equiv), and molecular sieves 4 Å were added. The obtained slurry mixture was intensively stirred at ambient temperature until cyanohydrin had been fully reacted (24–72 h). Then the insoluble components were filtered off on Celite, and the filtrate was concentrated in vacuo. The obtained residue was then mixed with brine and extracted several times with EtOAc. After extraction, the combined organic phases were treated with anhydrous MgSO_4_ to remove residual moisture, then subjected to filtration and solvent evaporation under reduced pressure. The resulting crude material was subsequently purified via flash chromatography.

##### 4.1.2.1.1 (3-Chloro-4-fluorophenyl)(4-fluoro-4-(((2-((6-methoxypyridin-2-yl)oxy)ethyl)amino)methyl)piperidin-1-yl)methanone (**25**)

Compound **25** was synthesized using cyanohydrin **7** (0.120 g, 0.38 mmol), 2-((6-methoxypyridin-2-yl)oxy)ethanamine (**8**) (0.090 g, 0.54 mmol), DABCO (0.535 g, 4.78 mmol), NaCNBH_3_ (0.178 g, 2.98 mmol), FeSO_4_·7H_2_O (0.117 g, 0.42 mmol), and molecular sieves (0.900 g) in methanol (5 mL). Purification: DCM/methanol/NH_3(aq)_ (9.5/0.5/0.02, v/v/v), and then *n*-hexane/EtOAc/methanol/NH_3(aq)_ (6/3.5/0.5/0.02, v/v/v/v). Yield: 69%; colorless oil. ^1^H NMR (300 MHz, CDCl_3_): δ 7.52–7.43 (m, 2H), 7.33–7.23 (m, 1H), 7.22–7.12 (m, 1H), 6.29 (dd, *J* = 1.5, 7.9 Hz, 2H), 4.49 (br s, 1H), 4.41–4.32 (m, 2H), 3.88 (s, 3H), 3.67–3.53 (m, 1H), 3.46–3.10 (m, 2H), 3.02 (t, *J* = 5.3 Hz, 2H), 2.90–2.76 (m, 2H), 1.99 (br s, 2H), 1.83–1.56 (m, 3H). ^13^C NMR (75 MHz, CDCl_3_): δ 168.1, 163.0, 162.6, 158.8 (d, *J* = 253 Hz), 141.0, 132.9 (d, *J* = 4.6 Hz), 129.7, 127.1 (d, *J* = 8.1 Hz), 121.5 (d, *J* = 18.4 Hz), 116.8 (d, *J* = 22 Hz), 101.3, 101.2, 94.3 (d, *J* = 172 Hz), 65.2, 57.3 (d, *J* = 22 Hz), 53.4, 49.2, 43.5, 38.3, 33.6, 32.8. Formula: C_21_H_24_ClF_2_N_3_O_3_; MS (ESI^+^) *m*/*z*: 440 [M + H^+^].

##### 4.1.2.1.2 (3-Chloro-4-fluorophenyl)(4-fluoro-4-(((2-((5-methoxypyridin-2-yl)oxy)ethyl)amino)methyl)piperidin-1-yl)methanone Formate Salt (**26**)

Compound **26** was synthesized using cyanohydrin **7** (0.120 g, 0.38 mmol), 2-((5-methoxypyridin-2-yl)oxy)ethanamine (**9**) (0.083 g, 0.50 mmol), DABCO (0.535 g, 4.78 mmol), NaCNBH_3_ (0.178 g, 2.98 mmol), FeSO_4_·7H_2_O (0.117 g, 0.42 mmol), and molecular sieves (0.900 g) in methanol (5 mL). Purification: *n*-hexane/Et_2_O/DCM/methanol/NH_3(aq)_ (6/2/1.5/0.5/0.02, v/v/v/v/v) and preparative HPLC (10–60% MeCN in Water with 0,05% HCOOH).Yield: 38%; yellow transparent oil. ^1^H NMR (500 MHz, CDCl_3_): δ 8.25 (s, 1H), 7.73 (d, *J* = 3.2 Hz, 1H), 7.47 (dd, *J* = 2.0, 6.9 Hz, 1H), 7.32–7.12 (m, 3H), 6.69 (d, *J* = 9.2 Hz, 1H), 6.53 (br s, 2H), 4.59–4.43 (m, 1H), 4.37 (t, *J* = 4.9 Hz, 2H), 3.79 (s, 3H), 3.69–3.53 (m, 1H), 3.46–3.27 (m, 1H), 3.23–3.05 (m, 3H), 3.01–2.91 (m, 2H), 2.01 (br s, 2H), 1.67 (br s, 2H); ^13^C NMR (126 MHz, CDCl_3_): δ 168.22, 166.31, 157.95, 158.92 (d, *J* = 252.7 Hz), 151.49, 132.78 (d, *J* = 4.2 Hz), 130.91, 129.87 (d, *J* = 2.2 Hz), 127.21 (d, *J* = 7.3 Hz), 126.98, 121.66 (d, *J* = 18.1 Hz), 116.92 (d, *J* = 21.4 Hz), 111.49, 93.86 (d, *J* = 172.9 Hz), 64.76, 56.32, 56.36 (br d, *J* = 21.7 Hz), 49.20, 43.54, 38.18, 33.07; Formula: C_21_H_24_ClF_2_N_3_O_3_·C_1_H_2_O_2_; MS (ESI^+^) *m*/*z*: 440 [M + H^+^].

##### 4.1.2.1.3 (3-Chloro-4-fluorophenyl)(4-fluoro-4-(((2-((4-methoxypyridin-2-yl)oxy)ethyl)amino)methyl)piperidin-1-yl)methanone (**27**)

Compound **27** was synthesized using cyanohydrin **7** (0.120 g, 0.38 mmol), 2-((4-methoxypyridin-2-yl)oxy)ethanamine (**10**) (0.090 g, 0.54 mmol), DABCO (0.535 g, 4.78 mmol), NaCNBH_3_ (0.178 g, 2.98 mmol), FeSO_4_·7H_2_O (0.117 g, 0.42 mmol), and molecular sieves (0.900 g) in methanol (5 mL). Purification: *n*-hexane/EtOAc/DCM/methanol/NH_3(aq)_ (1/1/7.5/0.5/0.02, v/v/v/v/v). Yield: 46%; colorless oil. ^1^H NMR (500 MHz, CDCl_3_): δ 7.93 (d, *J* = 6.0 Hz, 1H), 7.47 (dd, *J* = 2.0, 6.9 Hz, 1H), 7.29 (ddd, *J* = 2.0, 4.6, 8.3 Hz, 1H), 7.20–7.14 (m, 1H), 6.47 (dd, *J* = 2.1, 5.9 Hz, 1H), 6.20 (d, *J* = 2.0 Hz, 1H), 4.52 (br s, 1H), 4.37 (t, *J* = 5.3 Hz, 2H), 3.80 (s, 3H), 3.58 (br s, 1H), 3.37 (br s, 1H), 3.16 (br s, 1H), 3.01 (t, *J* = 5.2 Hz, 2H), 2.83 (br d, *J* = 19.5 Hz, 2H), 2.02 (br s, 2H), 1.66 (br s, 3H). ^13^C NMR (126 MHz, CDCl_3_): δ 168.15, 167.99, 165.65, 158.87 (d, *J* = 252.4 Hz), 147.48, 133.00 (d, *J* = 4.2 Hz), 129.81, 127.17 (d, *J* = 7.6 Hz), 121.62 (d, *J* = 18.1 Hz), 116.88 (d, *J* = 21.6 Hz), 106.38, 94.19, 94.48 (d, *J* = 172.0 Hz), 65.37, 57.35 (d, *J* = 22.2 Hz), 55.28, 49.38, 43.75 (br s), 38.29 (br s), 33.71 (br s), 32.83 (br s). Formula: C_21_H_24_ClF_2_N_3_O_3_; MS (ESI^+^) *m*/*z*: 440 [M + H^+^].

##### 4.1.2.1.4 (3-Chloro-4-fluorophenyl)(4-fluoro-4-(((2-((3-methoxypyridin-2-yl)oxy)ethyl)amino)methyl)piperidin-1-yl)methanone (**28**)

Compound **28** was synthesized using cyanohydrin **7** (0.134 g, 0.43 mmol), 2-[(3-methoxypyridin-2-yl)oxy]ethanamine (**11**) (0.115 g, 0.69 mmol), DABCO (0.600 g, 5.35 mmol), NaCNBH_3_ (0.210 g, 3.34 mmol), and molecular sieves (0.888 g) in methanol (4 mL). (Note that in this procedure, the FeSO_4_ × 7H_2_O was not used). Purification: EtOAc/methanol (9.5/0.5, v/v). Yield: 28%; yellow crystallizing oil. ^1^H NMR (300 MHz, CDCl_3_): δ 7.70 (dd, *J* = 1.4, 5.0 Hz, 1H), 7.47 (dd, *J* = 2.1, 6.9 Hz, 1H), 7.32–7.26 (m, 1H), 7.22–7.13 (m, 1H), 7.05 (dd, *J* = 1.4, 7.8 Hz, 1H), 6.84 (dd, *J* = 5.1, 7.7 Hz, 1H), 4.48 (t, *J* = 5.5 Hz, 3H), 3.85 (s, 3H), 3.60 (br s, 1H), 3.44–3.15 (m, 2H), 3.08 (t, *J* = 5.4 Hz, 2H), 2.92–2.77 (m, 2H), 1.98 (br s, 2H), 1.66 (br s, 3H). ^19^F NMR (282 MHz, CDCl_3_): δ – 112.7 (s, 1F), −166.4 (s, 1F). ^13^C NMR (75 MHz, CDCl_3_): δ 168.1, 158.8(d, *J* = 254 Hz), 153.9, 144.1, 136.9, 132.9 (d, *J* = 4.4 Hz), 129.7, 127.1 (d, *J* = 6.9 Hz), 121.5 (d, *J* = 18.4 Hz), 17.4, 117.0, 116.8 (d, *J* = 22 Hz), 94.4 (d, *J* = 172 Hz), 65.4, 57.2 (d, *J* = 22 Hz), 55.6, 49.1, 43.7, 38.3, 33.5, 32.8. Formula: C_21_H_24_ClF_2_N_3_O_3_; MS (ESI^+^) *m*/*z*: 440 [M + H^+^].

##### 4.1.2.1.5 (3-Chloro-4-fluorophenyl)(4-fluoro-4-(((2-((6-fluoropyridin-2-yl)oxy)ethyl)amino)methyl)piperidin-1-yl)methanone (**29**)

Compound **29** was synthesized using cyanohydrin **7** (0.050 g, 0.16 mmol), 2-[(6-fluoropyridin-2-yl)oxy]ethanamine hydrochloride (**12**) (0.034 g, 0.18 mmol), DABCO (0.178 g, 1.59 mmol), NaCNBH_3_ (0.078 g, 1.24 mmol), FeSO_4_·7H_2_O (0.049 g, 0.18 mmol), and molecular sieves (0.500 g) in methanol (3 mL). Purification: *n*-hexane/EtOAc/methanol/NH_3(aq)_ (6/3.5/0.5/0.02, v/v/v/v) and then *n*-hexane/DCM/methanol//NH_3(aq)_ (6/3.5/0.5/0.02, v/v/v/v). Yield: 44%; beige oil. ^1^H NMR (500 MHz, CDCl_3_): δ 7.65 (q, *J* = 8.1 Hz, 1H), 7.47 (dd, *J* = 2.0, 7.0 Hz, 1H), 7.28 (ddd, *J* = 2.0, 4.5, 8.3 Hz, 1H), 7.20–7.13 (m, 1H), 6.61 (dd, *J* = 1.0, 8.0 Hz, 1H), 6.47 (dd, *J* = 2.4, 7.7 Hz, 1H), 4.58–4.45 (m, 1H), 4.40 (t, *J* = 5.2 Hz, 2H), 3.60 (br s, 1H), 3.46–3.12 (m, 2H), 3.08 (t, *J* = 5.2 Hz, 2H), 2.94 (br s, 1H), 2.93–2.87 (m, 2H), 2.02 (br s, 2H), 1.84–1.52 (m, 2H). ^13^C NMR (126 MHz, CDCl_3_): δ 168.2, 162.6 (d, *J* = 13.3 Hz), 162.2 (d, *J* = 241.1 Hz), 158.9 (d, *J* = 252.7 Hz), 142.9 (d, *J* = 8.1 Hz), 132.8 (d, *J* = 4.3 Hz), 129.9, 127.2 (d, *J* = 7.6 Hz), 121.7 (d, *J* = 18.3 Hz), 116.9 (d, *J* = 21.6 Hz), 107.3 (d, *J* = 5.1 Hz), 100.5 (d, *J* = 35.2 Hz), 94.1 (d, *J* = 172.6 Hz), 65.3, 56.7 (d, *J* = 21.9 Hz), 48.8, 44.0–43.1 (m), 38.61–37.6 (m), 33.9–33.2 (m), 33.1–32.3 (m). Formula: C_20_H_21_ClF_3_N_3_O_2_; MS (ESI^+^) *m*/*z*: 428 [M + H^+^].

##### 4.1.2.1.6 (3-Chloro-4-fluorophenyl)(4-fluoro-4-(((2-((5-fluoropyridin-2-yl)oxy)ethyl)amino)methyl)piperidin-1-yl)methanone (**30**)

Compound **30** was synthesized using cyanohydrin **7** (0.100 g, 0.32 mmol), 2-((5-fluoropyridin-2-yl)oxy)ethanamine (**13**) (0.065 g, 0.41 mmol), DABCO (0.444 g, 3.97 mmol), NaCNBH_3_ (0.155 g, 2.48 mmol), FeSO_4_·7H_2_O (0.097 g, 0.35 mmol), and molecular sieves (0.900 g) in methanol (5 mL). Purification: *n*-hexane/Et_2_O/DCM/methanol/NH_3(aq)_ (6/2/1.5/0.5/0.02, v/v/v/v/v). Yield: 52%; colorless oil. ^1^H NMR (300 MHz, CDCl_3_): δ 7.96 (d, *J* = 2.9 Hz, 1H), 7.47 (dd, *J* = 2.1, 6.7 Hz, 1H), 7.38–7.26 (m, 2H), 7.22–7.11 (m, 1H), 6.70 (dd, *J* = 3.2, 9.1 Hz, 1H), 4.50 (br s, 1H), 4.39–4.28 (m, 2H), 3.74–3.48 (m, 1H), 3.46–3.06 (m, 2H), 3.00 (t, *J* = 5.3 Hz, 2H), 2.90–2.74 (m, 2H), 2.01 (br s, 2H), 1.62 (br s, 3H). ^19^F NMR (282 MHz, CDCl_3_): δ – 112.6 (s, 1F), −139.2 (s, 1F), −166.6 (s, 1F). ^13^C NMR (75 MHz, CDCl_3_): δ 168.1, 159.8, 158.8 (d, *J* = 252 Hz), 155.4 (d, *J* = 246 Hz), 133.3, 132.9 (d, *J* = 4.6 Hz), 129.7, 127.1 (d, *J* = 8.1 Hz), 126.6 (d, *J* = 22 Hz), 121.5 (d, *J* = 17.3 Hz), 116.8 (d, *J* = 22 Hz), 111.5 (d, *J* = 3.5 Hz), 94.4 (d, *J* = 172 Hz), 65.7, 57.3 (d, *J* = 22 Hz), 49.1, 43.5, 38.1, 33.8, 32.7. Formula: C_20_H_21_ClF_3_N_3_O_2_; MS (ESI^+^) *m*/*z*: 428 [M + H^+^].

##### 4.1.2.1.7 (3-Chloro-4-fluorophenyl)(4-fluoro-4-(((2-((4-fluoropyridin-2-yl)oxy)ethyl)amino)methyl)piperidin-1-yl)methanone (**31**)

Compound **31** was synthesized using cyanohydrin **7** (0.100 g, 0.32 mmol), 2-((4-fluoropyridin-2-yl)oxy)ethanamine hydrochloride (**14**) (0.080 g, 0.41 mmol), DABCO (0.444 g, 3.97 mmol), NaCNBH_3_ (0.155 g, 2.48 mmol), FeSO_4_ × 7H_2_O (0.097 g, 0.35 mmol), and molecular sieves (0.900 g) in methanol (5 mL). Purification: *n*-hexane/Et_2_O/DCM/methanol/NH_3(aq)_ (6/2/1.5/0.5/0.02, v/v/v/v/v). Yield: 43%; colorless oil. ^1^H NMR (300 MHz, CDCl_3_): δ 8.08 (dd, *J* = 5.9, 8.8 Hz, 1H), 7.47 (dd, *J* = 1.8, 7.0 Hz, 1H), 7.33–7.27 (m, 1H), 7.23–7.11 (m, 1H), 6.65 (ddd, *J* = 2.3, 5.7, 7.8 Hz, 1H), 6.43 (dd, *J* = 2.3, 10.0 Hz, 1H), 4.51 (br s, 1H), 4.40 (t, *J* = 5.3 Hz, 2H), 3.58 (br s, 1H), 3.47–3.07 (m, 2H), 3.01 (t, *J* = 5.3 Hz, 2H), 2.83 (d, *J* = 19.9 Hz, 2H), 2.01 (br s, 2H), 1.58 (br s, 3H). ^19^F NMR (282 MHz, CDCl_3_): δ – 101.7 (s, 1F), −112.6 (s, 1F), and −166 6 (s, 1F). ^13^C NMR (75 MHz, CDCl_3_): δ 170.2 (d, *J* = 259 Hz), 168.0, 165.7 (d, *J* = 12.7 Hz), 158.8 (d, *J* = 253 Hz), 148.8 (d, *J* = 8.1 Hz), 132.9 (d, *J* = 4.6 Hz), 129.7, 127.1 (d, *J* = 6.9 Hz), 121.5 (d, *J* = 18.4 Hz), 116.8 (d, *J* = 22 Hz), 106.2 (d, *J* = 18.4 Hz), 97.8 (d, *J* = 20.8 Hz), 94.4 (d, *J* = 172 Hz), 65.9, 57.3 (d, *J* = 22 Hz), 49.1, 43.7, 38.3, 34.0, 32.6. Formula: C_20_H_21_ClF_3_N_3_O_2_; MS (ESI^+^) *m*/*z*: 428 [M + H^+^].

##### 4.1.2.1.8 (3-Chloro-4-fluorophenyl)(4-(((2-((6-chloropyridin-2-yl)oxy)ethyl)amino)methyl)-4-fluoropiperidin-1-yl)methanone (**32**)

Compound **32** was synthesized using cyanohydrin **7** (0.120 g, 0.38 mmol), 2-((6-chloropyridin-2-yl)oxy)ethanamine (**15**) (0.092 g, 0.54 mmol), DABCO (0.535 g, 4.78 mmol), NaCNBH_3_ (0.178 g, 2.98 mmol), FeSO_4_·7H_2_O (0.117 g, 0.42 mmol), and molecular sieves (0.900 g) in methanol (5 mL). Purification: *n*-hexane/Et_2_O/DCM/methanol/NH_3(aq)_ (6/2/1.5/0.5/0.02, v/v/v/v/v). Yield: 47%; orange oil. ^1^H NMR (300 MHz, CDCl_3_): δ 7.55–7.41 (m, 2H), 7.33–7.25 (m, 1H), 7.21–7.12 (m, 1H), 6.88 (d, *J* = 7.6 Hz, 1H), 6.64 (d, *J* = 8.2 Hz, 1H), 4.50 (br s, 1H), 4.38 (t, *J* = 5.3, 2H), 3.60 (d, *J* = 19.9 Hz, 1H), 3.46–3.09 (m, 2H), 3.00 (t, *J* = 5.3, 2H), 2.89–2.73 (d, *J* = 20.5, 2H), 1.99 (br s, 2H), 1.82–1.49 (m, 3H). ^13^C NMR (75 MHz, CDCl_3_): δ 168.0, 163.3, 158.7 (d, *J* = 254 Hz), 148.3, 140.7, 132.9 (d, *J* = 3.5 Hz), 129.7, 127.1 (d, *J* = 8.1 Hz), 121.5 (d, *J* = 18.4 Hz), 116.8 (d, *J* = 22 Hz), 116.5, 109.1, 94.4 (d, *J* = 172 Hz), 65.8, 57.1 (d, *J* = 22 Hz), 49.0, 43.7, 38.3, 33.7, 32.8. Formula: C_20_H_21_Cl_2_F_2_N_3_O_2_; MS (ESI^+^) *m*/*z*: 444 [M + H^+^].

##### 4.1.2.1.9 (3-Chloro-4-fluorophenyl)(4-(((2-((5-chloropyridin-2-yl)oxy)ethyl)amino)methyl)-4-fluoropiperidin-1-yl)methanone Formate Salt

Compound **33** was synthesized using cyanohydrin **7** (0.300 g, 0.96 mmol), 2-[(5-chloropyridin-2-yl)oxy]ethanamine (**16**) (0.230 g, 1.34 mmol), DABCO (1.34 g, 12.00 mmol), NaCNBH_3_ (0.467 g, 7.45 mmol), and molecular sieves (2.080 g) in methanol (9.5 mL). (Note that in this procedure the FeSO_4_·7H_2_O was not used). Purification: EtOAc/methanol (9.8/0.2, v/v) and *n*-hexane/EtOAc/methanol/NH_3(aq)_ (3/6.5/0.5/0.02, v/v/v/v) and preparative HPLC (10–60% MeCN in Water with 0,05% HCOOH). Yield: 21%; colorless oil. ^1^H NMR (500 MHz, CDCl_3_): δ 8.20 (s, 1H), 8.03 (d, *J* = 2.7 Hz, 1H), 7.52 (dd, *J* = 2.6, 8.8 Hz, 1H), 7.45 (dd, *J* = 2.1, 6.9 Hz, 1H), 7.28–7.25 (m, 1H), 7.17–7.12 (m, 1H), 6.69 (d, *J* = 8.7 Hz, 1H), 5.43–5.39 (m, 2H), 4.50 (br s, 1H), 4.42–4.39 (m, 2H), 3.69–3.50 (m, 1H), 3.41–3.22 (m, 1H), 3.20–3.05 (m, 3H), 3.00–2.92 (m, 2H), 2.00 (br d, *J* = 7.2 Hz, 2H), 1.84–1.50 (m, 2H); ^13^C NMR (126 MHz, CDCl_3_): δ 168.3, 166.3, 161.8, 158.9 (d, *J* = 253 Hz), 145.1, 139.1, 132.7 (d, *J* = 4.2 Hz), 129.8, 127.2 (d, *J* = 7.6 Hz), 124.8, 121.7 (d, *J* = 18.1 Hz), 116.9 (d, *J* = 21.4 Hz), 112.3, 93.7 (d, *J* = 173.3 Hz), 64.5, 56.1 (d, *J* = 21.7 Hz), 48.7, 43.5, 38.0, 33.4, 32.7. Formula: C_20_H_21_Cl_2_F_2_N_3_O_2_ C_1_H_2_O_2_; MS (ESI^+^) *m*/*z*: 444 [M + H^+^].

##### 4.1.2.1.10 (3-Chloro-4-fluorophenyl)(4-fluoro-4-(((2-((6-(trifluoromethyl)pyridin-2-yl)oxy)ethyl)amino)methyl)piperidin-1-yl)methanone (**34**)

Compound **34** was synthesized using cyanohydrin **7** (0.120 g, 0.38 mmol), 2-((6-(trifluoromethyl)pyridin-2-yl)oxy)ethanamine (**17**) (0.110 g, 0.54 mmol), DABCO (0.535 g, 4.78 mmol), NaCNBH_3_ (0.178 g, 2.98 mmol), FeSO_4_·7H_2_O (0.117 g, 0.42 mmol), and molecular sieves (0.900 g) in methanol (5 mL). Purification: *n*-hexane/EtOAc/methanol/NH_3(aq)_ (6/3.5/0.5/0.02, v/v/v/v). Yield: 45%; colorless transparent oil. ^1^H NMR (500 MHz, CDCl_3_): δ 7.70 (t, *J* = 7.9 Hz, 1H), 7.47 (dd, *J* = 2.0, 6.9 Hz, 1H), 7.28 (ddd, *J* = 1.9, 4.6, 8.3 Hz, 1H), 7.26–7.24 (m, 1H), 7.19–7.13 (m, 1H), 6.92 (d, *J* = 8.3 Hz, 1H), 4.53 (br s, 1H), 4.49 (t, *J* = 5.2 Hz, 2H), 3.58 (br s, 1H), 3.45–3.12 (m, 2H), 3.08 (br t, *J* = 5.1 Hz, 2H), 3.00–2.82 (m, 3H), 2.00 (br s, 2H), 1.63 (br s, 2H). ^13^C NMR (126 MHz, CDCl_3_): δ 168.2, 163.6, 158.9 (d, *J* = 252.5 Hz), 145.5 (q, *J* = 34.8 Hz), 139.7, 132.9 (d, *J* = 4.3 Hz), 129.8, 127.2 (d, *J* = 7.6 Hz), 121.6 (d, *J* = 18.1 Hz), 121.4 (q, *J* = 273.9 Hz), 116.9 (d, *J* = 21.4 Hz), 114.7 (d, *J* = 0.9 Hz), 113.6 (q, *J* = 3.0 Hz), 94.2 (d, *J* = 172.3 Hz), 65.3, 56.9 (d, *J* = 21.9 Hz), 48.9, 43.6, 38.2, 33.5, 32.8. Formula: C_21_H_21_ClF_5_N_3_O_2_; MS (ESI^+^) *m*/*z*: 478 [M + H^+^].

##### 4.1.2.1.11 (3-Chloro-4-fluorophenyl)(4-fluoro-4-(((2-((4-(trifluoromethyl)pyridin-2-yl)oxy)ethyl)amino)methyl)piperidin-1-yl)methanone (**35**)

Compound **35** was synthesized using cyanohydrin **7** (0.300 g, 0.96 mmol), 2-{[4-(trifluoromethyl)pyridin-2-yl]oxy}ethanamine (**18**) (0.276 g, 1.34 mmol), DABCO (1.34 g, 12.00 mmol), NaCNBH_3_ (0.467 g, 7.45 mmol), and molecular sieves (2.080 g) in methanol (9.5 mL). Purification: EtOAc/methanol (9.8/0.2, v/v) and then *n*-hexane/EtOAc/methanol/NH_3(aq)_ (6/3.5/0.5/0.02, v/v/v/v). Yield: 17%; colorless oil. ^1^H NMR (500 MHz, CDCl_3_): δ 8.27 (d, *J* = 5.3 Hz, 1H), 7.47 (dd, *J* = 1.9, 6.9 Hz, 1H), 7.31–7.26 (m, 1H), 7.19–7.14 (m, 1H), 7.08 (d, *J* = 5.2 Hz, 1H), 6.98 (s, 1H), 4.58–4.48 (m, 1H), 4.46 (t, *J* = 5.1 Hz, 2H), 3.70–3.53 (m, 1H), 3.46–3.29 (m, 1H), 3.23–3.11 (m, 1H), 3.08 (t, *J* = 5.1 Hz, 2H), 2.88 (br d, *J* = 19.9 Hz, 2H), 2.02 (br s, 2H), 1.66 (br s, 2H), N*H* proton not detected. ^13^C NMR (126 MHz, CDCl_3_): δ 168.20, 164.02, 158.91 (d, *J* = 252.5 Hz), 148.32, 141.16 (q, *J* = 33.0 Hz), 132.88 (d, *J* = 4.2 Hz), 129.84, 127.20 (d, *J* = 7.6 Hz), 122.24 (q, *J* = 273.0 Hz), 121.65 (d, *J* = 18.1 Hz), 122.64 (q, *J* = 273.0 Hz), 116.91 (d, *J* = 21.6 Hz), 112.66 (q, *J* = 3.2 Hz), 107.94 (q, *J* = 4.1 Hz), 94.23 (d, *J* = 172.3 Hz), 65.77, 57.05 (d, *J* = 22.0 Hz), 49.04, 43.70, 38.24, 33.58, 32.92. Formula: C_21_H_21_ClF_5_N_3_O_2_; MS (ESI^+^) *m*/*z*: 478 [M + H^+^].

##### 4.1.2.1.12 (3-Chloro-4-fluorophenyl)(4-fluoro-4-(((2-((6-(methylamino)pyridin-2-yl)oxy)ethyl)amino)methyl)piperidin-1-yl)methanone (**36**)

Compound **36** was synthesized using cyanohydrin **7** (0.139 g, 0.44 mmol), 6-(2-aminoethoxy)-*N*-methylpyridin-2-amine (**19**) (0.090 g, 0.53 mmol), DABCO (0.099 g, 0.89 mmol), NaCNBH_3_ (0.044 g, 0.71 mmol), FeSO_4_ × 7H_2_O (0.136 g, 0.49 mmol), and molecular sieves (0.900 g) in methanol (5 mL). Purification: *n*-hexane/Et_2_O/DCM/methanol/NH_3(aq)_ (6/2/1.5/0.5/0.02, v/v/v/v/v). Yield: 40%; orange oil. ^1^H NMR (500 MHz, CDCl_3_): δ 7.48 (dd, *J* = 2.0, 6.9 Hz, 1H), 7.36 (t, *J* = 7.9 Hz, 1H), 7.29 (ddd, *J* = 1.9, 4.6, 8.5 Hz, 1H), 7.20–7.15 (m, 1H), 6.01 (d, *J* = 8.0 Hz, 1H), 5.94 (d, *J* = 7.7 Hz, 1H), 4.51 (br s, 1H), 4.41 (br s, 1H), 4.30 (t, *J* = 5.3 Hz, 2H), 3.59 (br s, 1H), 3.38 (br s, 1H), 3.16 (br s, 1H), 3.00 (br t, *J* = 5.2 Hz, 2H), 2.87 (br s, 3H), 2.83 (br d, *J* = 20.0 Hz, 2H), 2.00 (br s, 2H), 1.76 (br s, 3H). ^19^F NMR (282 MHz, CDCl_3_): δ – 112.7 (s, 1F), −166.3 (s, 1F). ^13^C NMR (126 MHz, CDCl_3_): δ 168.1, 163.2, 158.7, 158.9 (d, *J* = 252 Hz), 140.2, 133.0 (d, *J* = 4.2 Hz), 129.8, 127.2 (d, *J* = 7.6 Hz), 121.6 (d, *J* = 18.1 Hz), 116.9 (d, *J* = 21.4 Hz), 97.4, 97.35, 94.5 (d, *J* = 172 Hz), 64.9, 57.4 (d, *J* = 22.3 Hz), 49.5, 43.8 (br s), 38.3 (br d, *J* = 5.1 Hz), 33.6 (br d, *J* = 27.3 Hz), 32.8 (br d, *J* = 19.2 Hz), 29.3. Formula: C_21_H_25_ClF_2_N_4_O_2_; MS (ESI^+^) *m*/*z*: 439 [M + H^+^].

##### 4.1.2.1.13 (3-Chloro-4-fluorophenyl)(4-(((2-((6-(dimethylamino)pyridin-2-yl)oxy)ethyl)amino)methyl)-4-fluoropiperidin-1-yl)methanone (**37**)

Compound **37** was synthesized using cyanohydrin **7** (0.120 g, 0.38 mmol), 6-(2-aminoethoxy)-*N*,*N*-dimethylpyridin-2-amine (**20**) (0.090 g, 0.50 mmol), DABCO (0.535 g, 4.78 mmol), NaCNBH_3_ (0.178 g, 2.98 mmol), FeSO_4_ × 7H_2_O (0.117 g, 0.42 mmol), and molecular sieves (0.900 g) in methanol (5 mL). Purification: *n*-hexane/Et_2_O/DCM/methanol/NH_3(aq)_ (6/2/1.5/0.5/0.02, v/v/v/v/v). Yield: 51%; brown oil. ^1^H NMR (300 MHz, CDCl_3_): δ 7.47 (dd, *J* = 1.8, 7.0 Hz, 1H), 7.40–7.32 (m, 1H), 7.31–7.26 (m, 1H), 7.20–7.11 (m, 1H), 5.99 (dd, *J* = 7.6, 15.2 Hz, 2H), 4.49 (br s, 1H), 4.40–4.32 (m, 2H), 3.58 (br s, 1H), 3.46–3.06 (m, 2H), 3.05–2.94 (m, 8H), 2.83 (d, *J* = 19.9 Hz, 2H), 2.00 (br s, 2H), 1.72 (br s, 3H). ^19^F NMR (282 MHz, CDCl_3_): δ – 112.7 (s, 1F), −166.3 (s, 1F). ^13^C NMR (75 MHz, CDCl_3_): δ 168.0, 162.5, 158.3, 158.7 (d, *J* = 254 Hz), 139.8, 132.9 (d, *J* = 3.5 Hz), 129.7, 127.1 (d, *J* = 8.1 Hz), 121.5 (d, *J* = 18.4 Hz), 116.8 (d, *J* = 22 Hz), 97.2, 96.1, 94.3 (d, *J* = 172 Hz), 64.6, 57.3 (d, *J* = 22 Hz), 49.4, 43.7, 38.3, 37.9 (2C), 33.6, 32.8. Formula: C_22_H_27_ClF_2_N_4_O_2_; MS (ESI^+^) *m*/*z*: 453 [M + H^+^].

##### 4.1.2.1.14 *N*-(6-(2-(((1-(3-Chloro-4-fluorobenzoyl)-4-fluoropiperidin-4-yl)methyl)amino)ethoxy)pyridin-2-yl)acetamide Formate Salt (**38**)

Compound **38** was synthesized using cyanohydrin **7** (0.100 g, 0.32 mmol), *N*-(6-fluoropyridin-2-yl)acetamide (**21**) (0.081 g, 0.41 mmol), DABCO (0.444 g, 3.97 mmol), NaCNBH_3_ (0.155 g, 2.48 mmol), FeSO_4_·7H_2_O (0.097 g, 0.35 mmol), and molecular sieves (0.800 g) in methanol (7 mL). Purification: DCM/methanol/NH_3(aq)_ (9.5/0.5/0.02, v/v/v) and preparative HPLC (10–60% MeCN in Water with 0,05% HCOOH). Yield: 60%; orange oil. ^1^H NMR (500 MHz, CDCl_3_): δ 8.42 (br s, 1H), 7.75 (br d, *J* = 7.6 Hz, 1H), 7.59 (t, *J* = 7.9 Hz, 1H), 7.46 (dd, *J* = 2.0, 6.9 Hz, 1H), 7.27 (ddd, *J* = 2.0, 4.5, 8.4 Hz, 1H), 7.20–7.13 (m, 1H), 6.46 (d, *J* = 8.0 Hz, 1H), 4.49 (br s, 1H), 4.43–4.20 (m, 5H), 3.71–3.50 (m, 1H), 3.45–3.25 (m, 1H), 3.22–3.03 (m, 3H), 2.94 (br d, *J* = 19.9 Hz, 2H), 2.19 (s, 3H), 2.03 (br s, 2H), 1.67 (br s, 2H). ^19^F NMR (282 MHz, CDCl_3_): δ – 112.5 (s, 1F), −166.4 (s, 1F). ^13^C NMR (126 MHz, CDCl_3_): δ 169.0, 168.2, 167.0, 162.1, 159.0 (d, *J* = 253 Hz), 149.3, 141.6, 132.7 (d, *J* = 4.3 Hz), 129.9, 127.2 (d, *J* = 7.6 Hz), 121.7 (d, *J* = 18.1 Hz), 117.0 (d, *J* = 21.4 Hz), 106.4, 105.9, 93.8 (d, *J* = 173.2 Hz), 64.7, 56.6 (d, *J* = 21.6 Hz), 49.6, 43.5, 38.1, 33.4, 33.0, 24.7. Formula: C_22_H_25_ClF_2_N_4_O_3_·C_1_H_2_O_2_; MS (ESI^+^): *m*/*z* 467 [M + H^+^].

##### 4.1.2.1.15 (3-Chloro-4-fluorophenyl)(4-(((2-((6-methylpyridin-2-yl)oxy)ethyl)amino)methyl)-4-fluoropiperidin-1-yl)methanone (**39**)

Compound **39** was synthesized using cyanohydrin **7** (0.120 g, 0.38 mmol), 2-((6-methylpyridin-2-yl)oxy)ethanamine (**22**) (0.081 g, 0.54 mmol), DABCO (0.535 g, 4.78 mmol), NaCNBH_3_ (0.178 g, 2.98 mmol), FeSO_4_·7H_2_O (0.117 g, 0.42 mmol), and molecular sieves (0.900 g) in methanol (5 mL). Purification: *n*-hexane/Et_2_O/DCM/methanol/NH_3(aq)_ (3/2/4.5/0.5/0.02, v/v/v/v/v). Yield: 76%; colorless oil. ^1^H NMR (300 MHz, CDCl_3_): δ 7.51–7.40 (m, 2H), 7.32–7.26 (m, 1H), 7.17 (t, *J* = 8.5 Hz, 1H), 6.71 (d, *J* = 7.0 Hz, 1H), 6.51 (d, *J* = 8.2 Hz, 1H), 4.48 (br s, 1H), 4.41–4.33 (m, 2H), 3.58 (br s, 1H), 3.47–3.06 (m, 3H), 3.01 (t, *J* = 5.3 Hz, 2H), 2.83 (d, *J* = 20.5 Hz, 2H), 2.42 (s, 3H), 2.00 (br s, 2H), 1.80 (br s, 3H). ^13^C NMR (75 MHz, CDCl_3_): δ 168.0, 163.2, 158.8 (d, *J* = 252 Hz), 156.3, 138.9, 132.9 (d, *J* = 4.6 Hz), 129.7, 127.1 (d, *J* = 6.9 Hz), 121.5 (d, *J* = 17.5 Hz), 116.8 (d, *J* = 21 Hz), 115.9, 107.0, 94.4 (d, *J* = 173 Hz), 65.0, 57.2 (d, *J* = 22 Hz), 49.4, 43.7, 38.2, 33.6, 32.9, 24.1. Formula: C_21_H_24_ClF_2_N_3_O_2_; MS (ESI^+^) *m*/*z*: 424 [M + H^+^].

##### 4.1.2.1.16 (3-Chloro-4-fluorophenyl)(4-(((2-((5-methylpyridin-2-yl)oxy)ethyl)amino)methyl)-4-fluoropiperidin-1-yl)methanone (**40**)

Compound **40** was reported previously by Sniecikowska et al.^22^

##### 4.1.2.1.17 (3-Chloro-4-fluorophenyl)(4-fluoro-4-(((2-((6-(pyrrolidin-1-yl)pyridin-2-yl)oxy)ethyl)amino)methyl)piperidin-1-yl)methanone Formate Salt

Compound **41** was synthesized using cyanohydrin **7** (0.106 g, 0.34 mmol), 2-{[6-(pyrrolidin-1-yl)pyridin-2-yl]oxy}ethanamine hydrochloride (**23**) (0.105 g, 0.51 mmol), DABCO (0.473 g, 4.23 mmol), NaCNBH_3_ (0.165 g, 2.64 mmol), FeSO_4_ × 7H_2_O (0.103 g, 0.37 mmol), and molecular sieves (1.043 g) in methanol (5 mL). Purification: *n*-hexane/EtOAc/methanol/NH_3(aq)_ (6/3.5/0.5/0.02, v/v/v/v) and preparative HPLC (10–60% MeCN in Water with 0,05% HCOOH). Yield: 58%; yellow oil. ^1^H NMR (500 MHz, CDCl_3_): δ 8.24 (s, 1H), 7.49 (dd, *J* = 1.9, 6.9 Hz, 1H), 7.36 (t, *J* = 7.9 Hz, 1H), 7.29 (ddd, *J* = 2.0, 4.5, 8.3 Hz, 1H), 7.21–7.16 (m, 1H), 6.86 (br s, 2H), 5.96 (d, *J* = 7.7 Hz, 1H), 5.92 (d, *J* = 8.0 Hz, 1H), 4.57 (br s, 1H), 4.50 (br t, *J* = 4.6 Hz, 2H), 3.73–3.54 (m, 1H), 3.41 (br t, *J* = 6.4 Hz, 5H), 3.26 (br s, 3H), 3.09 (br d, *J* = 19.8 Hz, 2H), 2.07–1.94 (m, 6H), 1.71 (br s, 2H). ^13^C NMR (126 MHz, CDCl_3_): δ 168.28, 166.05, 162.32, 158.97 (d, *J* = 252.8 Hz), 156.31, 140.05, 132.61 (d, *J* = 4.3 Hz), 129.90, 127.23 (d, *J* = 7.6 Hz), 121.71 (d, *J* = 18.1 Hz), 116.95 (d, *J* = 21.6 Hz), 98.66, 95.54, 93.17 (d, *J* = 174.1 Hz), 62.60, 55.63 (d, *J* = 21.6 Hz), 48.90, 46.76 (2C), 43.36, 38.05, 33.17, 32.87, 25.53 (2C). Formula: C_24_H_29_ClF_2_N_4_O_2_·C_1_H_2_O_2_; MS (ESI^+^) *m*/*z*: 479 [M + H^+^].

##### 4.1.2.1.18 (4-(((2-((6-(1*H*-Pyrazol-1-yl)pyridin-2-yl)oxy)ethyl)amino)methyl)-4-fluoropiperidin-1-yl)(3-chloro-4-fluorophenyl)methanone Formate Salt (**42**)

Compound **42** was synthesized using cyanohydrin **7** (0.094 g, 0.30 mmol), 2-{[6-(1*H*-pyrazol-1-yl)pyridin-2-yl]oxy}ethanamine (**24**) (0.100 g, 0.42 mmol), DABCO (0.417 g, 3.73 mmol), NaCNBH_3_ (0.146 g, 2.32 mmol), FeSO_4_·7H_2_O (0.091 g, 0.33 mmol), and molecular sieves (0.700 g) in methanol (4 mL). Purification: *n*-hexane/EtOAc/methanol/NH_3(aq)_ (6/3.5/0.5/0.02, v/v/v/v) and preparative HPLC (10–60% MeCN in Water with 0,05% HCOOH). Yield: 96%; beige oil. ^1^H NMR (500 MHz, CDCl_3_): δ 8.44 (d, *J* = 2.6 Hz, 1H), 8.15 (br s, 1H), 7.73–7.68 (m, 2H), 7.50 (d, *J* = 7.9 Hz, 1H), 7.47 (dd, *J* = 1.9, 6.9 Hz, 1H), 7.27 (ddd, *J* = 1.9, 4.5, 8.2 Hz, 1H), 7.19–7.13 (m, 1H), 6.63 (d, *J* = 8.0 Hz, 1H), 6.44–6.41 (m, 1H), 5.35 (br s, 2H), 4.54 (br t, *J* = 4.7 Hz, 2H), 4.52–4.45 (m, 1H), 3.72–3.50 (m, 1H), 3.44–3.26 (m, 1H), 3.21 (br t, *J* = 4.6 Hz, 2H), 3.13 (br s, 1H), 3.00 (br d, *J* = 19.9 Hz, 2H), 2.02 (br s, 2H), 1.69 (br s, 2H)·Formula: C_23_H_24_ClF_2_N_5_O_2_·C_1_H_2_O_2_; MS (ESI^+^) *m*/*z*: 476 [M + H^+^].

### 4.2 Developability Studies (In Silico)

The SwissADME server was used to assess PAINS, toxicophore alerts (Brenk), and the fraction of sp^3^ atoms. Structure database management and prediction of additional physicochemical parameters were carried out using Instant JChem 15.12.14.0, 2015, ChemAxon (http://www.chemaxon.com).

### 4.3 Determination of Compounds’ Physicochemical Properties and Their Solubility

The physicochemical properties of compounds **27** and **31** and their solubilities were determined using a Sirius T3 instrument (Pion Inc., Forest Row, UK), according to the provided manufacturer’s instructions.

The acidity (p*K*_**a**_ parameter) was obtained by automated pH-metric titrations using the methanol cosolvent. Three independent measurements of the apparent p*K*_a_ (psKa) were conducted for each compound, with the weight % content of the methanol 50%, 40% and 30%, respectively. The obtained psKa values were next extrapolated to the 0% content of methanol (pure water), using the Yasuda-Shedlovsky extrapolation plot.

The lipophilicity of tested compounds (log P parameter) was also measured by the automated potentiometric method based on the previously obtained p*K*_a_ value. The aqueous pH-metric titrations were carried out in the presence of *n*-octanol - a water-immiscible partition solvent. Under those circumstances, a lipophilic sample partitioned into the octanol layer and caused a shift to the aqueous phase equilibria, resulting in the appearance of the apparent p*K*_a_ (poKa). The log *P* parameter was determined based on the differences between the apparent poKa and aqueous p*K*_a_ values, in three independent titrations conducted with diverse volume ratios of the aqueous and *n*-octanol phases.

The aqueous intrinsic solubility measurements (log *S*_0_) were also conducted based on the obtained p*K*_a_ values, using acid–base titration automated methods.^34^ The samples were fully dissolved in their ionized states at the beginning of the assays and titrated with base toward their p*K*_a_ values. As the pH of the solutions approached the p*K*_a_ values, the tested compounds converted into the neutral state. With increasing concentrations of the neutral forms, the compounds reached their solubility and then precipitated until equilibrium was established. The pH at which the equilibrium solubility Bjerrum curve (mean molecular charge vs pH) deviated from the in-solution p*K*_a_ Bjerrum curve was used to calculate the intrinsic solubility. Due to the samples’ fast precipitation kinetics, the compounds were found to be nonchasers (i.e., not supersaturating before the precipitation), and subsequently, the curve-Fitting solubility method was applied for log *S*_0_ solubility parameters measurements.

### 4.4 In Vitro Studies

Before the in vitro studies, the SwissADME tool^39^ was used to check for pan-assay-interference compounds (PAINS). No PAINS were detected.

#### 4.4.1 Preparation of Solutions of Test and Reference Compounds

Tested and reference compounds were initially dissolved in dimethyl sulfoxide (DMSO) to obtain 10 mM stock solutions. Using an automated pipetting workstation (epMotion 5070, Eppendorf), serial dilutions were performed in 96-well plates with assay buffer, maintaining a final DMSO content of 0.1%. Each compound was evaluated across 8 to 12 concentrations.

#### 4.4.2 Competition Binding Methodology for 5-HT_1A_R, α_1_R, D_2_R

The comprehensive methodology has been described in our previous publications, which provide a detailed explanation of the experimental conditions.^22,23^ Briefly, binding assays were performed using either membranes from CHO-K1 cells expressing human 5-HT_1A_ or D_2_ receptors (PerkinElmer) or rat cortex tissue for α_1_ receptor studies. Tissue homogenization (α_1_) involved centrifugation at 20,000*g*, resuspension, and final dilution (10 mg/mL). Assays were conducted in duplicate with 50 μL of test compounds, 50 μL of radioligand [^3^H]-8-OH-DPAT (1 nM, 5-HT_1A_), [^3^H]-prazosin (0.2 nM, α_1_), or [^3^H]-methylspiperone (0.4 nM, D_2_), and 150 μL of membrane/tissue suspension in assay buffer. The following assay buffers were used for the analysis: 50 mM Tris, pH 7.4, 10 mM MgSO_4_, 0.5 mM EDTA, 0.1% ascorbic acid for 5-HT_1A_; 50 mM Tris-HCl buffer, pH 7.6 for α_1_; 50 mM Tris, pH 7.4, 50 mM *N*-(2-hydroxyethyl)piperazine-*N*′-ethanesulfonic acid, 50 mM NaCl, 5 mM MgCl_2_, and 0.5 mM EDTA for D_2_. Nonspecific binding was determined using serotonin (10 μM, 5-HT_1A_), phentolamine (10 μM, α_1_), or haloperidol (10 μM, D_2_).

After incubation, reactions were terminated by rapid filtration through GF/C or GF/B filter mates pretreated with polyethyleneimine. Filters were washed (5 to 10 x, ice-cold Tris buffer), dried (37 °C), and coated with MeltiLex scintillator (90 °C, 4–5 min). Radioactivity was measured by using a MicroBeta2 scintillation counter (PerkinElmer). Data were analyzed using Prism 6 (GraphPad), and *K*_i_ values were calculated via the Cheng–Prusoff equation.

#### 4.4.3 Functional Assays for the 5-HT_1A_ Receptor

##### 4.4.3.1 Results Analysis

The *E*_MAX_ values were calculated by expressing the ligand response as a percentage of the maximum effect induced by 5-HT, with calculations made using nonlinear regression in GraphPad Prism 6.0 software. The pEC_50_ values represent the ligand concentration required to reach 50% of the maximal response.

##### 4.4.3.2 ERK1/2 Phosphorylation Assay

The CHO-5HT_1A_ receptor cells were used to determine agonist-induced ERK1/2 phosphorylation using the SureFire ERK1/2-phosphorylation AlphaLISA assay kit, following the manufacturer’s guidelines (PerkinElmer). The detailed methodology has been outlined in our previous publication, where the experimental conditions are thoroughly explained.^22,23^ Briefly, cells were cultured in Advanced Dulbecco’s modified Eagle’s medium/F12 medium with 1% dialyzed saline (FBS), 400 μg/mL G-418, and 4 mM l-glutamine. Next 5 × 10^4^ cells were seeded per well in a 96-well plate and incubated for few hours at 37 °C with 5% CO2. After incubation, the cells were starved for 12 h in medium with 0.1% bovine serum albumin. Compounds were serially diluted and added to the cells, followed by a 15 min incubation. Subsequently, the medium was removed, and lysis buffer was added. The next day, after thawing, the 10 μL of the lysate was transferred to assay plates, and the AlphaLISA SureFire Ultra reaction mix (PerkinElmer) was added and plates were incubated. Following incubation, the assay plate was read by using an EnVision plate reader (PerkinElmer Life Science).

##### 4.4.3.3 cAMP Inhibition Assay

The functional assay was performed with CHO-K1 cells transfected with a plasmid containing the 5-HT_1A_ human serotonin receptor coding sequence. The comprehensive methodology is provided in our previous publication, where the experimental conditions are extensively described.^22,23^ In brief, thawed cells were resuspended in stimulation buffer at a concentration of 2 × 10^5^ cells/mL. Equal volumes of cell suspension were mixed to tested compounds and forskolin. After 40 min of incubation at room temperature, the reagent LANCE Ultra cAMP kit (PerkinElmer, USA) was added. 1 h later, the cAMP levels were measured with the EnVision microplate reader (PerkinElmer, USA).

##### 4.4.3.4 β-Arrestin Recruitment Assay

The HTR1A-bla U2OS receptor cells, containing human5-HT_1A_ receptor were tested for agonist-induced activity using the Tango LiveBLAzer assay kit (Life Technologies).^22,23^ In summary, the cells were cultured in an appropriate medium with 10% dialyzed FBS and other supplements. For the experiment, 10,000 cells were plated in a 384-well black plate and incubated for 12 h in an incubator at 37 °C with 5% CO2. Serially diluted compounds were added, followed by a 5 h incubation. After addition of 8 μL of reaction mix, the plate was incubated for 2 h at RT. The assay was analyzed using a FLUOstar Optima plate reader (BMG Labtech).

##### 4.4.3.5 Calcium Mobilization Assay

A cellular aequorin-based functional assay was used. The detailed methodology can be found in the publication.^23^ Briefly, after thawing, the cells were resuspended in assay buffer and incubated with coelenterazine h for 16 h at 16 °C. The cell suspension was diluted to 100,000 cells/mL. Following this, 50 μL of the cell suspension was added to the compounds-preloaded assay microplates, calcium-induced light emission was measured for 30 s using MicroBeta2 LumiJET plate counter (PerkinElmer, USA).

### 4.5 In Vitro ADME Studies

*Eurofins Pharma Discovery Services* performed permeability, metabolic stability, and plasma protein binding assays using well-established methodologies. We did not conduct any studies involving human-derived samples obtained directly from human subjects. The plasma protein binding and metabolic stability studies were performed using commercially available human plasma and human liver microsomes by Eurofins Discovery, and all procedures were carried out according to the company’s established protocols and approvals. Additional details can be found on the company’s Web site (www.eurofinsdiscoveryservices.com) and in relevant publications.^31−33^

### 4.6 In Vivo Pharmacokinetic Studies

#### 4.6.1 Application to a Pharmacokinetic Study in Rats

Male Wistar rats (180 g, 210 g) obtained from a certified animal facility at Jagiellonian University Medical College (Poland) were used. Animals were housed in groups of four under controlled conditions (21 ± 2 °C, 50–60% humidity, 12 h light/dark cycle starting at 08:00), with unrestricted access to standard food (LSM-B) and filtered water. The animals were deprived of food overnight before the drug administration but had unrestricted access to water. Compounds **27** and **31** (25 mg) were mixed with 20 mL of a 0.5% aqueous Tween-80 solution containing 6.75 mg acetic acid, sonicated at room temperature until complete dissolution (approximately 30 min), and then given orally (p.o.) at a single screening dose of 2.5 mg/kg. The animals were exterminated by decapitation at 5, 15, 30, 60, and 120 min following compound administration (*n* = 5–6 per time point), and blood samples (approximately 5–6 mL) were collected in tubes. Blood was permitted to clot for 15 to 20 min at room temperature, followed by centrifugation for 10 min at a speed of 3000 rpm (Bionovo LMC-3000 centrifuge). Additionally, brains were extracted from skulls and rinsed with 0.9% sodium chloride. The collected serum and brains were stored at −80 °C until analysis. The studies received approval from the Local Ethical Committee for Experiments on Animals at Jagiellonian University in Krakow, Poland, no. 371/2020.

#### 4.6.2 Methods Used in Pharmacokinetic Studies

##### 4.6.2.1 Instruments

##### Quantitative UPLC-MS/MS Analysis

A quantitative UPLC-MS/MS method was developed for analysis of the compounds under investigation. The UPLC-MS/MS system consisted of a Waters Acquity Premier coupled with a Waters Xevo TQ-S Cronos mass spectrometer (ESI-tandem quadrupole), UPLC BEH C_18_ column (2.1 × 100 mm, and 1.7 μm, 40 °C), and C_18_ VanGuard precolumn (2.1 × 5 mm, and 1.7 μm, 40 °C). The elution was carried out using the following conditions: isocratic elution with 95% of water + 0.1% v/v of formic acid over 0.5 min, afterward linear gradient elution from 95% to 0% of eluent A over 3.5 min, and 100% of acetonitrile +0.1% v/v of formic acid over 1.5 min; 0.3 mL/min. Samples were tested in triplicate.

Waters Xevo TQ-S Cronos mass spectrometer was calibrated for quantitative analysis using compounds **27** and **31** solutions in concentrations 50 μg mL^–1^ at a flow of 20 μL min^–1^ and a mixture of water + 0.1% v/v formic acid and acetonitrile + 0.1% v/v formic acid in ratio 1:1 (v/v) at a flow 0.28 mL min^–1^. Traces of the analyzed compounds were analyzed using the MRM (Multiple Reaction Monitoring) method under optimized conditions. Key parameters: source temp 150 °C, desolvation temp 250 °C, desolvation gas 600 L/h, cone gas 50 L/h, capillary 0.50 kV, collision cell pressure 2.7 × 10^–3^ mBar, N_2_ as nebulizing/drying gas, Ar as collision gas, cone potential and collision energy were individually optimized for each transition (Supporting Information Table S7). MassLynx V4.2 (Waters) was used for the data processing. Calibration curves were linear from 0.5 to 200 ng/(mL) in serum and brain homogenates. Precision and accuracy (intra/inter-day) remained within 15%. Lower limit of quantification was 0.025 ng/(mL or g).

##### 4.6.2.2 Sample Preparations

##### Preparation of Serum and Brain Samples before Solid-Phase Extraction (SPE)

Before conducting the analysis, brain samples were thawed and weighed (100–150 mg), subsequently transferred into 3 mL plastic tubes containing three volumes (w/v) of phosphate-buffered saline (PBS, pH 7.4) for individual homogenization utilizing a MICCRA D-1 homogenizer (ART Prozess & Labortechnik GmbH & Co., Germany). All brain homogenates were extracted following the same protocol used for the serum samples. Samples (100 μL) of serum or brain homogenate containing compounds **27** or **31** were mixed in Eppendorf tubes with 10 μL of internal standard solution (compound **3**—NLX-204; 100 ng/mL for serum, 150 ng/g for brain) and 200 μL of 2% formic acid in methanol. The samples underwent mixing and centrifugation for 15 min at 14,000 rpm. Next, 200 μL of supernatant was collected in new Eppendorf tubes and evaporated in a concentrating centrifuge (Eppendorf, Concentrator plus, Eppendorf, Poland) at 30 °C for 1 h.

##### 4.6.2.3 SPE-Based Extraction Procedure

SPE was performed using an Oasis PRiME MCX (Waters, USA) cartridge containing 60 mg sorbent per cartridge and a Waters SPE Extraction Manifold, 20-position (Waters, USA). Before use, each cartridge was conditioned with 2 mL of methanol, then 2 mL of water. To the dry residue prepared before SPE, 100 μL of water–acetonitrile mixture (1:1; v/v) and 200 μL of 4% H_3_PO_4_ in water were added, the whole was mixed and applied (approximately 300 μL) to the SPE column. The cartridge was then washed twice, first with 2 mL of 100 mM ammonium formate in 2% formic acid and then with 2 mL of methanol. Finally, the column was eluted with 4 mL (2 × 2 mL) of 5% NH_3_ in methanol. The eluate (approximately 4 mL) was evaporated to dryness in a vacuum centrifuge at 30 °C, and the dry residue was dissolved in 200 μL of a water–acetonitrile mixture (1:1; v/v). Extracts were analyzed directly on the same day by UPLC-MS/MS.

##### 4.6.2.4 Data Analysis

The serum and brain concentration–time profiles were analyzed using the noncompartmental method within the Phoenix WinNonlin v. 8.4 software (Pharsight Corp., Mountain View, CA, USA). Cmax and *T*_max_ were directly derived from individual concentration versus time profiles. λ_*z*_ was evaluated through linear regression, and the t_0.5λz_ was determined as ln 2/λ_*z*_. AUC_0–∞_ was computed by utilizing the linear trapezoidal rule. The extrapolated terminal area is defined as *C*_n_/λ_*z*_, in which *C*_n_ represents the final data point. CL/F was computed as *D*/AUC_0–∞_. *V*_z_/*F* was estimated as *D*/(λ_z_ × AUC_0–∞_), with *F* indicating the fraction of the dose absorbed.

### 4.7 In Vivo Pharmacodynamic Studies

#### 4.7.1 Animals

Male Wistar rats (180–210 g) obtained from a certified animal facility at Jagiellonian University Medical College (Poland) were used. The conditions of animal housing were the same as in vivo pharmacokinetic studies, but standard laboratory food (LSM-B) and an enrichment environment were freely available during all procedures. All procedures were carried out between 9:00 and 14:00 by two independent observers blinded to the treatment conditions. Each animal, assigned randomly to a separate group, was tested only once. All experimental procedures involving animals were conducted in accordance with Guidance on the operation of the Animals (Scientific Procedures) Act 1986 and associated guidelines, EU Directive 2010/63 for the protection of animals used for scientific purposes, and Polish legislation acts concerning animal experimentation and approved by the II Local Ethics Committee for Experiments on Animals in Cracow, Poland (approval number: 108/2016 and 371/2020). All animal experiments comply with ARRIVE guidelines.^40^ All efforts were made to minimize suffering and in accordance with 3R’s rules. 6–8 animals per group were used.

#### 4.7.2 Drugs

The weighted sample of each tested compound: **31** and gepirone, were mixed with an appropriate amount of aqueous solution of Tween-80 (R) (0.5%) (e.g., 20 mL) with an addition of 2 eq of acetic acid and sonicated at room temperature until completely dissolved (c.a. Thirty min.). WAY100635 (Tocris, UK) was dissolved, while haloperidol (Haloperidol WZF 5 mg/mL) was diluted in distilled water. All compounds were prepared immediately before administration in a volume of 2 mL/kg. The investigated compounds (**31** and gepirone) were given orally (p.o.) 60 min before tests, while WAY100635 and haloperidol were administered subcutaneously (s.c.) 75 and 60 min, respectively, before testing. Control animals received vehicles according to the same schedule.

#### 4.7.3 Forced Swim Test

The experiment was carried out according to the method of Porsolt et al.,^41^ adapted in our laboratory and described by Sniecikowska et al., 2020.^23^ Briefly, rats were placed twice in water-filled plexiglass cylinders (17 cm of water, 23–25 °C): first for 15 min (pretest) and then for 5 min (test). Immobility time was recorded during the second session. After testing, the animals were dried under a 60 W lamp. Immobility was defined as minimal movement required to keep the head above water.^42^ Fresh water was used for each rat.

#### 4.7.4 CLP Test for Catalepsy

Rats were placed on the stainless-steel floor, abdomen toward the floor, and the hind paws brought forward and the front paws backward so that the ipsilateral hind paws could hold onto the top of the front paws, and the time the rat stayed in this position recorded up to 30 s.^43,44^ Mean time in seconds from 3 trials (every 3 min 60 min after tested compounds and haloperidol administration) was reported for each rat, and then the mean of 8 rats per dose was compared to a vehicle using one-way ANOVA. Animals were put back in their home cage after each set of tests.

#### 4.7.5 Locomotor Activity—Open Field Test

The experiment took place in a darkened room using the Motor Monitor System (Campden Instruments, Ltd.) consisting of four Smart Frame Open Field stations (40 × 40 × 38 cm) with 16 × 16 beams, located in sound attenuating chambers and connected to PC software by control chassis. After injection of the investigated compounds, the animals were gently placed in the center of the station. Ambulation (in *X* and *Y* axis) and total distance covered by a rat for 5 min were recorded.

#### 4.7.6 Statistical Analysis

Behavioral data were analyzed using one-way ANOVA (one drug) or two-way ANOVA (two drugs jointly), depending on treatment design, followed by Bonferroni’s post hoc test (*p* < 0.05).

## Supporting Information Available

Dose–response curves for functional activity; results of the in vivo pharmacodynamic studies of compound **31** and gepirone; detailed procedures for preparation of the amine intermediates **8–24**; spectra of the target compounds (^1^H NMR, ^19^F NMR, ^13^C NMR, LC–MS); and HPLC traces of the final compounds (PDF)
Molecular formula strings (CSV)

## Abbreviations

5-HT_1A_/5-HT_1A_R: serotonin 1A receptor
5-MeO–DMT: 5-methoxy-*N*,*N*-dimethyltryptamine
8-OH-DPAT: 8-hydroxy-2-(di-*n*-propylamino)tetralin
*A*UC_0-∞_: area under the serum concentration–time curve from time zero to infinity
CHO-K1: Chinese hamster ovary cells
*C*_max_: maximum concentration
CMS: Chronic Mild Stress
CNS MPO: central nervous system multiparameter optimization
CPL: cross-leg position
EtOAc: ethyl acetate
Fsp^3^: fraction of sp3 carbon atoms
FST: forced swimming test (Porsolt test)
HLM: human liver microsomes
LLE: Ligand-lipophilicity efficiency
MeOH: methanol
NLX-101: aka F15599, 1-{[1-(3-chloro-4-fluorobenzoyl)-4-fluoropiperidin-4-yl]methyl}[(5-methylpyrimidin-2-yl)methyl]amine
NLX-112: aka F13640, 2-(3-chloro-4-fluorophenyl)(4-fluoro-4-(((2-((6-fluoropyridin-2-yl)oxy)ethyl)amino)methyl)piperidin-1-yl)methanone
NLX-266: 31-(3-Chloro-4-fluorophenyl)(4-fluoro-4-(((2-((4-fluoropyridin-2-yl)oxy)ethyl)amino)methyl)piperidin-1-yl)methanone
PAINS: pan-assay-interference compounds
RLM: rat liver microsomes
half-life in the elimination phase
*T*_max_: time to reach the maximum concentration
*V*_z_/F: volume of distribution at the elimination phase
WAY-100635: *N*-[2-[4-(2-methoxyphenyl)piperazin-1-yl]ethyl]-*N*-pyridin-2-ylcyclohexanecarboxamide

## References

[ref1] Pedigo N. W.; Yamamura H. I.; Nelson D. L. Discrimination of Multiple [3H]5-Hydroxytryptamine Binding Sites by the Neuroleptic Spiperone in Rat Brain. J. Neurochem. 1981, 36 (1), 220–226. 10.1111/j.1471-4159.1981.tb02397.x. 7463047

[ref2] Fargin A.; Raymond J. R.; Lohse M. J.; Kobilka B. K.; Caron M. G.; Lefkowitz R. J. The Genomic Clone G-21 Which Resembles a β-Adrenergic Receptor Sequence Encodes the 5-HT1A Receptor. Nature 1988, 335 (6188), 358–360. 10.1038/335358a0. 3138543

[ref3] Newman-Tancredi A.; Depoortère R.; Kleven M.; Kołaczkowski M.; Zimmer L. Translating Biased Agonists from Molecules to Medications: Serotonin 5-HT1A Receptor Functional Selectivity for CNS Disorders. Pharmacol. Ther. 2022, 229, 107937 10.1016/j.pharmthera.2021.107937. 34174274

[ref4] Giorgioni G.; Bonifazi A.; Botticelli L.; Cifani C.; Matteucci F.; Micioni Di Bonaventura E.; Micioni Di Bonaventura M. V.; Giannella M.; Piergentili A.; Piergentili A.; Quaglia W.; Del Bello F. Advances in Drug Design and Therapeutic Potential of Selective or Multitarget 5-HT1A Receptor Ligands. Med. Res. Rev. 2024, 44, 2640–2706. 10.1002/med.22049. 38808959

[ref5] Sniecikowska J.; Newman-Tancredi A.; Kolaczkowski M. From Receptor Selectivity to Functional Selectivity: The Rise of Biased Agonism in 5-HT1A Receptor Drug Discovery. Curr. Top. Med. Chem. 2019, 19 (26), 2393–2420. 10.2174/1568026619666190911122040. 31544717

[ref6] Głuch-Lutwin M.; Sałaciak K.; Gawalska A.; Jamrozik M.; Sniecikowska J.; Newman-Tancredi A.; Kołaczkowski M.; Pytka K. The Selective 5-HT1A Receptor Biased Agonists, F15599 and F13714, Show Antidepressant-like Properties after a Single Administration in the Mouse Model of Unpredictable Chronic Mild Stress. Psychopharmacology 2021, 238 (8), 2249–2260. 10.1007/s00213-021-05849-0. 33973045 PMC8292235

[ref7] Aguiar R. P.; Soares L. M.; Varney M.; Newman-Tancredi A A.; Milani H.; Prickaerts J.; de Oliveira R. M. W. NLX-101, a 5-HT1A Receptor-Biased Agonist, Improves Pattern Separation and Stimulates Neuroplasticity in Aged Rats. Neurobiol. Aging 2023, 124, 52–59. 10.1016/j.neurobiolaging.2022.12.013. 36739621

[ref8] Papp M.; Gruca P.; Litwa E.; Lason M.; Newman-Tancredi A.; Depoortère R. The 5-HT1A Receptor Biased Agonists, NLX-204 and NLX-101, like Ketamine, Elicit Rapid-Acting Antidepressant Activity in the Rat Chronic Mild Stress Model via Cortical Mechanisms. J. Psychopharmacol. Oxf. Engl. 2024, 38 (7), 661–671. 10.1177/02698811241254832. 38825869

[ref9] Papp M.; Gruca P.; Lason M.; Litwa E.; Newman-Tancredi A.; Depoortère R. The 5-HT1A Receptor Biased Agonists, NLX-204 and NLX-101, Display Ketamine-like RAAD and Anti-TRD Activities in Rat CMS Models. Psychopharmacology 2023, 240 (11), 2419–2433. 10.1007/s00213-023-06389-5. 37310446 PMC10593613

[ref10] Cabanu S.; Pilar-Cuéllar F.; Zubakina P.; Florensa-Zanuy E.; Senserrich J.; Newman-Tancredi A.; Adell A. Molecular Signaling Mechanisms for the Antidepressant Effects of NLX-101, a Selective Cortical 5-HT1A Receptor Biased Agonist. Pharmaceuticals 2022, 15 (3), 337 10.3390/ph15030337. 35337135 PMC8954942

[ref11] Bardin L.; Tarayre J. P.; Malfetes N.; Koek W.; Colpaert F. C. Profound Non-Opioid Analgesia Produced by the High-Efficacy 5-HT(1A) Agonist F 13640 in the Formalin Model of Tonic Nociceptive Pain. Pharmacology 2003, 67 (4), 182–194. 10.1159/000068404. 12595749

[ref12] Deseure K.; Bréand S.; Colpaert F. C. Curative-like Analgesia in a Neuropathic Pain Model: Parametric Analysis of the Dose and the Duration of Treatment with a High-Efficacy 5-HT(1A) Receptor Agonist. Eur. J. Pharmacol. 2007, 568 (1–3), 134–141. 10.1016/j.ejphar.2007.04.022. 17512927

[ref13] Courault P.; Lancelot S.; Costes N.; Colom M.; Le Bars D.; Redoute J.; Gobert F.; Dailler F.; Isal S.; Iecker T.; Newman-Tancredi A.; Merida I.; Zimmer L. [18F]F13640: A Selective Agonist PET Radiopharmaceutical for Imaging Functional 5-HT1A Receptors in Humans. Eur. J. Nucl. Med. Mol. Imaging 2023, 50 (6), 1651–1664. 10.1007/s00259-022-06103-1. 36656363 PMC10119077

[ref14] Colom M.; Vidal B.; Fieux S.; Redoute J.; Costes N.; Lavenne F.; Mérida I.; Irace Z.; Iecker T.; Bouillot C.; Billard T.; Newman-Tancredi A.; Zimmer L. [18F]F13640, a 5-HT1A Receptor Radiopharmaceutical Sensitive to Brain Serotonin Fluctuations. Front. Neurosci. 2021, 15, 622423 10.3389/fnins.2021.622423. 33762906 PMC7982540

[ref15] Study Details | Study to Assess the Safety, Tolerability and Preliminary Efficacy of NLX-112 Versus Placebo in L-dopa-induced Dyskinesia | ClinicalTrials.gov. https://www.clinicaltrials.gov/study/NCT05148884 (accessed Sep 18, 2024).

[ref16] News E. I. N.; Cerutti P. Neurolixis Announces Positive Ph2A Proof-of-Concept on NLX-112 in Levodopa-Induced Dyskinesia in Parkinson’s Disease. EIN News. https://www.einnews.com/pr_news/622375270/neurolixis-announces-positive-ph2a-proof-of-concept-on-nlx-112-in-levodopa-induced-dyskinesia-in-parkinson-s-disease (accessed Sep 18, 2024).

[ref17] Researchers hopeful of treatment of Parkinson’s by 2030 with dual efficacy drug. Independent. https://www.independent.co.uk/news/health/parkinsons-disease-drug-treatment-trial-b2370993.html (accessed Sep 18, 2024).

[ref18] Fisher R.; Hikima A.; Morris R.; Jackson M. J.; Rose S.; Varney M. A.; Depoortere R.; Newman-Tancredi A. The Selective 5-HT1A Receptor Agonist, NLX-112, Exerts Anti-Dyskinetic and Anti-Parkinsonian-like Effects in MPTP-Treated Marmosets. Neuropharmacology 2020, 167, 107997 10.1016/j.neuropharm.2020.107997. 32057799 PMC7103782

[ref19] Depoortere R.; Johnston T. H.; Fox S. H.; Brotchie J. M.; Newman-Tancredi A. The Selective 5-HT1A Receptor Agonist, NLX-112, Exerts Anti-Dyskinetic Effects in MPTP-Treated Macaques. Parkinsonism Relat. Disord. 2020, 78, 151–157. 10.1016/j.parkreldis.2020.08.009. 32846366

[ref20] McCreary A. C.; Varney M. A.; Newman-Tancredi A. The Novel 5-HT1A Receptor Agonist NLX-112 Reduces l-DOPA-Induced Abnormal Involuntary Movements in Rat: A Chronic Administration Study with Microdialysis Measurements. Neuropharmacology 2016, 105, 651–660. 10.1016/j.neuropharm.2016.01.013. 26777281

[ref21] Iderberg H.; McCreary A. C.; Varney M. A.; Kleven M. S.; Koek W.; Bardin L.; Depoortère R.; Cenci M. A.; Newman-Tancredi A. NLX-112, a Novel 5-HT1A Receptor Agonist for the Treatment of L-DOPA-Induced Dyskinesia: Behavioral and Neurochemical Profile in Rat. Exp. Neurol. 2015, 271, 335–350. 10.1016/j.expneurol.2015.05.021. 26037043

[ref22] Sniecikowska J.; Gluch-Lutwin M.; Bucki A.; Więckowska A.; Siwek A.; Jastrzebska-Wiesek M.; Partyka A.; Wilczyńska D.; Pytka K.; Pociecha K.; Cios A.; Wyska E.; Wesołowska A.; Pawłowski M.; Varney M. A.; Newman-Tancredi A.; Kolaczkowski M. Novel Aryloxyethyl Derivatives of 1-(1-Benzoylpiperidin-4-Yl)Methanamine as the Extracellular Regulated Kinases 1/2 (ERK1/2) Phosphorylation-Preferring Serotonin 5-HT1A Receptor-Biased Agonists with Robust Antidepressant-like Activity. J. Med. Chem. 2019, 62 (5), 2750–2771. 10.1021/acs.jmedchem.9b00062. 30721053

[ref23] Sniecikowska J.; Gluch-Lutwin M.; Bucki A.; Więckowska A.; Siwek A.; Jastrzebska-Wiesek M.; Partyka A.; Wilczyńska D.; Pytka K.; Latacz G.; Przejczowska-Pomierny K.; Wyska E.; Wesołowska A.; Pawłowski M.; Newman-Tancredi A.; Kolaczkowski M. Discovery of Novel pERK1/2- or β-Arrestin-Preferring 5-HT1A Receptor-Biased Agonists: Diversified Therapeutic-like versus Side Effect Profile. J. Med. Chem. 2020, 63 (19), 10946–10971. 10.1021/acs.jmedchem.0c00814. 32883072 PMC7586344

[ref24] Sniecikowska J., Bucki A., Newman-Tancredi A., Varney M. A. Compounds for Treating Disorders Sensitive to Serotoninergic Regulation Controlled by the 5-Ht1a Receptors. US Patent 2,019,194,132 A1 June 27, 2019 https://worldwide.espacenet.com/publicationDetails/biblio?FT=D&date=20190627&DB=&locale=en_EP&CC=US&NR=2019194132A1&KC=A1&ND=4/accessed/2024-08-23.

[ref25] Zhou Q.; Yang D.; Wu M.; Guo Y.; Guo W.; Zhong L.; Cai X.; Dai A.; Jang W.; Shakhnovich E. I.; Liu Z.-J.; Stevens R. C.; Lambert N. A.; Babu M. M.; Wang M.-W.; Zhao S. Common Activation Mechanism of Class A GPCRs. eLife 2019, 8, e50279 10.7554/eLife.50279. 31855179 PMC6954041

[ref26] Newman-Tancredi A.; Martel J.-C.; Assié M.-B.; Buritova J.; Lauressergues E.; Cosi C.; Heusler P.; Bruins Slot L.; Colpaert F. C.; Vacher B.; Cussac D. Signal Transduction and Functional Selectivity of F15599, a Preferential Post-Synaptic 5-HT1A Receptor Agonist. Br. J. Pharmacol. 2009, 156 (2), 338–353. 10.1111/j.1476-5381.2008.00001.x. 19154445 PMC2697830

[ref27] Warren A. L.; Lankri D.; Cunningham M. J.; Serrano I. C.; Parise L. F.; Kruegel A. C.; Duggan P.; Zilberg G.; Capper M. J.; Havel V.; Russo S. J.; Sames D.; Wacker D. Structural Pharmacology and Therapeutic Potential of 5-Methoxytryptamines. Nature 2024, 630 (8015), 237–246. 10.1038/s41586-024-07403-2. 38720072 PMC11152992

[ref28] Vacher B.; Maurel J. L.; Brunel S. Method for Preparing (3-Chloro-4-Fluorophenyl)-(4-Fluoro-4-{[(5methyl-Pyrimidin-2-Ylmethyl)-Amino]-Methyl}-Piperidin-1-Yl)-Methanone and Novel Intermediate Pyrimidine Derivatives, September 22, EP 1928857 B1, 2006. https://patents.google.com/patent/EP1928857A1/en (accessed Feb 15 2018).

[ref29] Tasker A.; Zhang D.; Cao G.-C.; Chakrabarti P.; Falsey J.; Herberich B.; Hungate R.; Pettus L.; Reed A.; Rzasa R.; Sham K.; Thaman T.; Xu S. Phthalazine., Aza- and Diaza-Phthalazine Compounds and Methods of Use, March 2, US Patent 20,060,199,817 A1, 2006. https://patents.google.com/patent/US20060199817A1/en (accessed 2018–02–16).

[ref30] Vacher B.; Bonnaud B.; Funes P.; Jubault N.; Koek W.; Assié M. B.; Cosi C. Design and Synthesis of a Series of 6-Substituted-2-Pyridinylmethylamine Derivatives as Novel, High-Affinity, Selective Agonists at 5-HT1A Receptors. J. Med. Chem. 1998, 41 (25), 5070–5083. 10.1021/jm9804329. 9836623

[ref31] Hidalgo I. J.; Raub T. J.; Borchardt R. T. Characterization of the Human Colon Carcinoma Cell Line (Caco-2) as a Model System for Intestinal Epithelial Permeability. Gastroenterology 1989, 96 (3), 736–749. 10.1016/0016-5085(89)90897-4. 2914637

[ref32] Obach R. S.; Baxter J. G.; Liston T. E.; Silber B. M.; Jones B. C.; MacIntyre F.; Rance D. J.; Wastall P. The Prediction of Human Pharmacokinetic Parameters from Preclinical and in Vitro Metabolism Data. J. Pharmacol. Exp. Ther. 1997, 283 (1), 46–58. 10.1016/S0022-3565(24)36999-X. 9336307

[ref33] Banker M. J.; Clark T. H.; Williams J. A. Development and Validation of a 96-Well Equilibrium Dialysis Apparatus for Measuring Plasma Protein Binding. J. Pharm. Sci. 2003, 92 (5), 967–974. 10.1002/jps.10332. 12712416

[ref34] Box K.; Comer J. E.; Gravestock T.; Stuart M. New Ideas about the Solubility of Drugs. Chem. Biodivers. 2009, 6 (11), 1767–1788. 10.1002/cbdv.200900164. 19937815

[ref35] Depoortère R.; Auclair A. L.; Newman-Tancredi A. NLX-101, a Highly Selective 5-HT1A Receptor Biased Agonist, Mediates Antidepressant-like Activity in Rats via Prefrontal Cortex 5-HT1A Receptors. Behav. Brain Res. 2021, 401, 113082 10.1016/j.bbr.2020.113082. 33358917

[ref36] Jastrzębska-Więsek M.; Partyka A.; Rychtyk J.; Śniecikowska J.; Kołaczkowski M.; Wesołowska A.; Varney M. A.; Newman-Tancredi A. Activity of Serotonin 5-HT1A Receptor Biased Agonists in Rat: Anxiolytic and Antidepressant-like Properties. ACS Chem. Neurosci. 2018, 9 (5), 1040–1050. 10.1021/acschemneuro.7b00443. 29266914

[ref37] Dovonou A.; Bolduc C.; Soto Linan V.; Gora C.; Peralta Iii M. R.; Lévesque M. Animal Models of Parkinson’s Disease: Bridging the Gap between Disease Hallmarks and Research Questions. Transl. Neurodegener. 2023, 12 (1), 36 10.1186/s40035-023-00368-8. 37468944 PMC10354932

[ref38] Cong S.; Xiang C.; Zhang S.; Zhang T.; Wang H.; Cong S. Prevalence and Clinical Aspects of Depression in Parkinson’s Disease: A Systematic Review and Meta-analysis of 129 Studies. Neurosci. Biobehav. Rev. 2022, 141, 104749 10.1016/j.neubiorev.2022.104749. 35750224

[ref39] Daina A.; Michielin O.; Zoete V. SwissADME: A Free Web Tool to Evaluate Pharmacokinetics, Drug-Likeness and Medicinal Chemistry Friendliness of Small Molecules. Sci. Rep. 2017, 7, 42717 10.1038/srep42717. 28256516 PMC5335600

[ref40] Kilkenny C.; Browne W.; Cuthill I. C.; Emerson M.; Altman D. G. Animal Research: Reporting in Vivo Experiments: The ARRIVE Guidelines. Br. J. Pharmacol. 2010, 160 (7), 1577–1579. 10.1111/j.1476-5381.2010.00872.x. 20649561 PMC2936830

[ref41] Porsolt R. D.; Anton G.; Blavet N.; Jalfre M. Behavioural Despair in Rats: A New Model Sensitive to Antidepressant Treatments. Eur. J. Pharmacol. 1978, 47 (4), 379–391. 10.1016/0014-2999(78)90118-8. 204499

[ref42] Detke M. J.; Rickels M.; Lucki I. Active Behaviors in the Rat Forced Swimming Test Differentially Produced by Serotonergic and Noradrenergic Antidepressants. Psychopharmacology 1995, 121 (1), 66–72. 10.1007/BF02245592. 8539342

[ref43] Prinssen E. P.; Kleven M. S.; Koek W. The Cataleptogenic Effects of the Neuroleptic Nemonapride Are Attenuated by Its 5-HT1A Receptor Agonist Properties. Eur. J. Pharmacol. 1998, 356 (2–3), 189–192. 10.1016/S0014-2999(98)00536-6. 9774248

[ref44] Depoortère R.; Bardin L.; Auclair A. L.; Kleven M. S.; Prinssen E.; Colpaert F.; Vacher B.; Newman-Tancredi A. F15063, a Compound with D2/D3 Antagonist, 5-HT1A Agonist and D4 Partial Agonist Properties: (II) Activity in Models of Positive Symptoms of Schizophrenia. Br. J. Pharmacol. 2007, 151 (2), 253–265. 10.1038/sj.bjp.0707159. 17375086 PMC2013947

