# Supplementary material for: Discovery of NLX-266, an Orally Available and Metabolically Stable ERK1/2-Biased 5-HT1AR Agonist with Superior Antidepressant and Antiparkinsonian Activity
Source: J Med Chem. 2025 Apr 23;68(9):9706–22. doi: 10.1021/acs.jmedchem.5c00484 (PMC12067441; doi:10.1021/acs.jmedchem.5c00484)
Supplement: Supplementary file 1 — jm5c00484_si_001.pdf [file jm5c00484_si_001.pdf]

Discovery of NLX-266, an orally available and metabolically stable ERK1/2-biased 5-HT_{1A}R agonist with superior antidepressant and antiparkinsonian activity

Joanna Sniecikowska [a], Monika Gluch-Lutwin [a], Adam Bucki [a], Beata Gryzlo [a], Krzysztof Wieckowski [a], Justyna Godyn [a], Anna Wieckowska [a], Agata Siwek [a], Magdalena Jastrzebska-Wiesek [a], Anna Partyka [a], Agnieszka Cios [a], Anna Wesolowska [a], Adrian Newman-Tancredi [b], Marcin Kolaczkowski [a]*

[a] Jagiellonian University Medical College, 9 Medyczna St., 30-688 Kraków, Poland;

[b] Neurolixis SAS, 81290 Labruguière, Castres, France

* Email: marcin.kolaczkowski@uj.edu.pl

Contents

1. Dose-response curves for functional activity. 2

2. Results of the in vivo pharmacodynamic studies 4

3. Detailed procedures for preparation of amine intermediates 8–24........ 7

4.1. General procedure for preparation of amines 8–11, 13, 15–18, and 22........ 7

4.2. Synthesis of amine 12 (2-((6-fluoropyridin-2-yl)oxy)ethanamine hydrochloride)........ 10

4.3. Synthesis of amine 14 (2-((4-fluoropyridin-2-yl)oxy)ethanamine hydrochloride)........ 11

4.4. General procedure for preparation of amines 19–21. 12

4.5. Synthesis of amine 23 (2-((6-(pyrrolidin-1-yl)pyridin-2-yl)oxy)ethanamine hydrochloride. 14

4.6. Synthesis of amine 24 (2-((6-(1H-pyrazol-1-yl)pyridin-2-yl)oxy)ethanamine). 14

4. Spectra of the target compounds (^{1}H NMR, ^{19}F NMR, ^{13}C NMR, LC-MS; Figures S1–S56)........ 16

5. HPLC traces of the final compounds........ 45

6. Optimized settings for quantitative analysis of the investigated compounds in the *In Vivo* Pharmacokinetic Studies 51

7. References 51

1. Dose-response curves for functional activity.

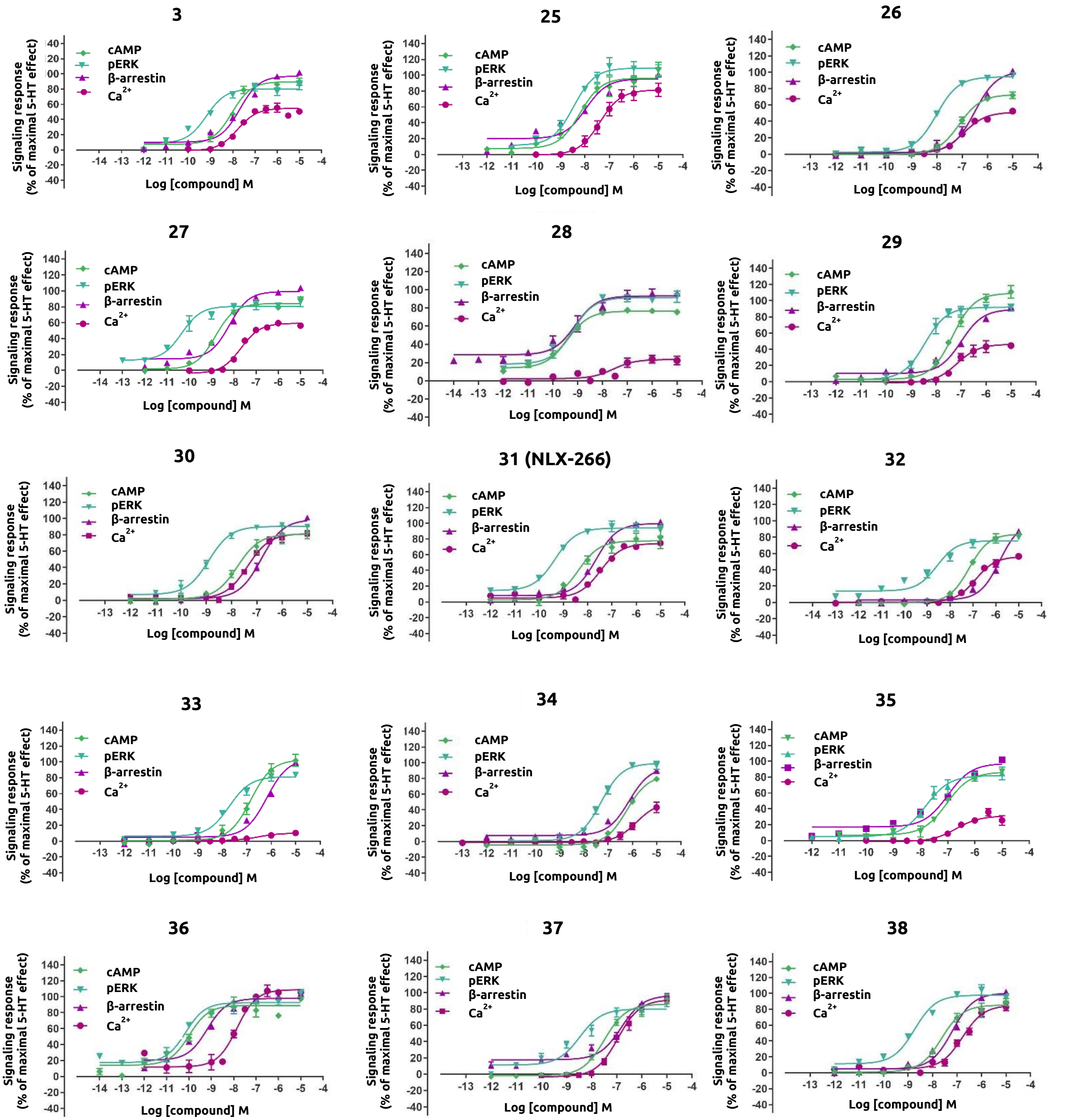

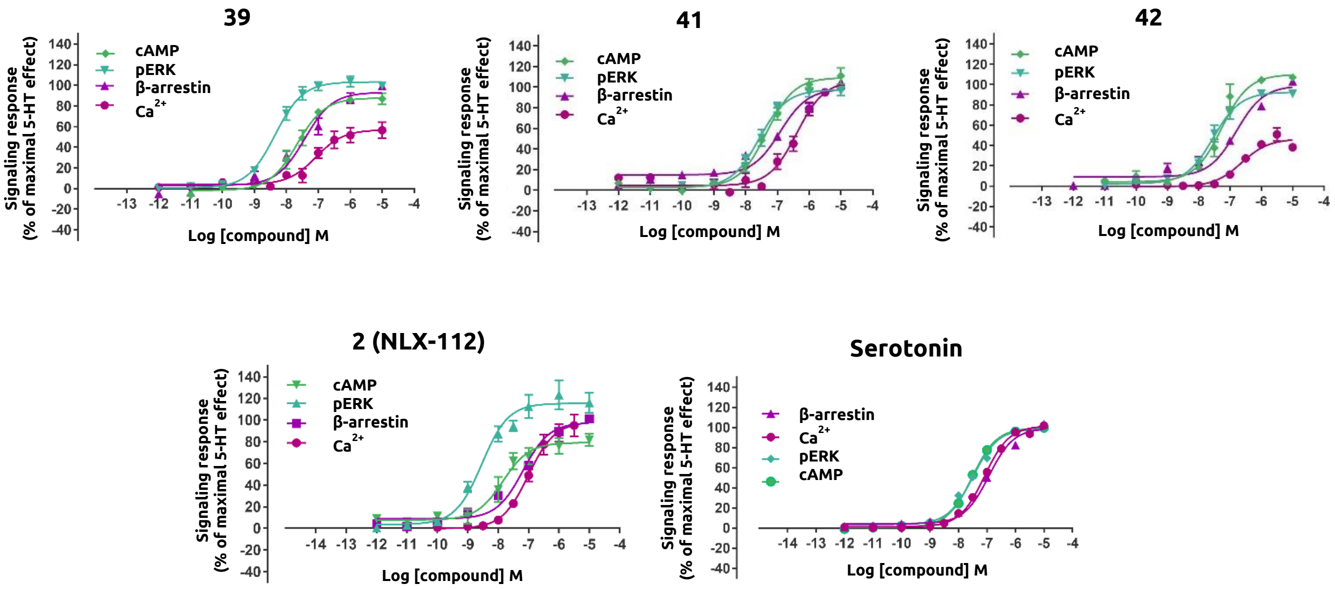
39
41
42
2 (NLX-112)
Serotonin
cAMP
pERK
β-arrestin
Ca2+
Signaling response (% of maximal 5-HT effect)
Log [compound] M

2. Results of the in vivo pharmacodynamic studies

Table S1. Effect of 31 in the forced swim test in rats.

compound	Dose (mg/kg)	Immobility time (s)
Vehicle (water)	0	202.50 ± 15.61
**31**	0.16	229.38 ± 15.05 ns vs veh
	0.63	69.14 ± 13.36; $p<0.0001$ vs veh
	2.5	8.00 ± 4.46; $p<0.00001$ vs veh<br>$F(3,26)=62.687$; $p<0.00001$

31 was given p.o. 60 min before the test. Values represent the mean ± SEM of the immobility time during 5-min test session compared to the respective vehicle group (one-way ANOVA followed by Bonferroni's post hoc test); N=7-8

Table S2. Effect of 31 and WAY100635 in the forced swim test in rats.

Compound	Dose (mg/kg)	Immobility time (s)
Vehicle (water) + vehicle	0 + 0	208.50 ± 7.14
**31** + vehicle	0.63 + 0	78.57 ± 14.07;<br>$p<0.00001$ vs veh; $p<0.00001$ vs WAY
WAY100635 + vehicle	0.63 + 0	260.43± 8.65;<br>$p<0.05$ vs veh; $p<0.00001$ vs NLX
WAY100635 + 31	0.63 + 0.63	187.37 ± 13.; p<42;<br>ns vs veh; $p<0.001$ vs WAY; $p<0.0001$ vs NLX<br>$F(1,26)=6.4323$; $p<0.05$

31 was given p.o. 60 min, while WAY100635 was injected s.c. 75 min before the test. Values represent the mean ± SEM of the immobility time during 5-min test session compared to the respective vehicle group (two-way ANOVA followed by Bonferroni's post hoc test); N=7-8

Table S3. Effect of Gepirone in the forced swim test in rats.

compound	Dose (mg/kg)	Immobility time (s)
Vehicle (water)	0	186.00 ± 10.18
gepirone	0.63	192.50 ± 14.27 ns vs veh
	2.5	164.00 ± 21.69; ns vs veh
	5.0	172.62 ± 12.48; ns vs veh<br>$F(3,28)=0.7093$; NS
Vehicle (water)	0	166.00 ± 17.90
gepirone	10	86.51 ± 15.78; $p<0.01$ vs veh
	20	115.17 ± 9.14; ns vs veh<br>$F(2,17)=7.2682$; $p<0.01$

Gepirone was given p.o. 60 min before the test. Values represent the mean ± SEM of the immobility time during 5-min test session compared to the respective vehicle group (one-way ANOVA followed by Bonferroni's post hoc test); N=8

Table S4. The ability of 31 to reduce haloperidol-induced catalepsy.

Compound	Dose (mg/kg)	Catalepsy (CLP)
Vehicle + vehicle	0	0
Haloperidol (0.63 mg/kg) + vehicle	0	14.50 ± 2.44; p<0.00001 vs veh
Haloperidol (0.63 mg/kg) + **31**	0.01	11.24 ± 2.27; p<0.01 vs veh; ns vs halo
	0.04	3.63 ± 1.61; ns vs veh; p<0.001 vs halo
	0.16	6.17 ± 2.53; ns vs veh; p<0.05 vs halo
	0.63	0.46 ± 0.30; ns vs veh; p<0.000001 vs halo
	2.5	0.08 ± 0.08; ns vs veh; p<0.00001 vs halo
		F(6,47)=11.502; p<0.00001

31 was given p.o. while haloperidol s.c. 60 min before the test. Values represent the mean ± SEM of the cross-leg position (CLP) compared to the vehicle group (one-way ANOVA followed by Bonferroni's post hoc test); N=8

Table S5. The ability of Gepirone to reduce haloperidol-induced catalepsy.

Compound	Dose (mg/kg)	CLP
Vehicle + vehicle	0 + 0	0
Haloperidol (0.63 mg/kg) + vehicle	0.63 + 0	12.50 ± 2.82; p<0.001 vs veh
Haloperidol (0.63 mg/kg) + gepirone	0.63 + 0.63	14.83 ± 1.47; p<0.00001 vs veh; ns vs halo
	0.63 + 1.25	16.38 ± 2.26; p<0.00001 vs veh; ns vs halo
	0.63 + 2.5	0.52 ± 0.52; ns vs veh; p<0.001 vs halo
		F(4,32)=29.825; p<0.0001

Gepirone was given p.o. while haloperidol s.c. 60 min before the test. Values represent the mean ± SEM of the cross-leg position (CLP) compared to the vehicle group (one-way ANOVA followed by Bonferroni's post hoc test); N=7-8

Table S6. Effect of 31 and gepirone in OF test.

Compound	Dose (mg/kg)	Total distance (cm)	X ambulation	Y ambulation	Fine movements
Vehicle	0	992 ± 49	260.17 ± 34.67	13.17 ± 10.02	640.83 ± 52.63
31	0.63	718 ± 167; p<0.05<br>F(1,10)=5.4169; p<0.05	131.50 ± 14.62; p<0.01<br>F(1,10)=11.692; p<0.01	78.50 ± 8.28; p<0.01<br>F(1,10)=15.794; p<0.01	263.83 ± 45.83; p<0.01<br>F(1,10)=29.444; p<0.01
Vehicle	0	992 ± 49	260.17 ± 34.67	13.17 ± 10.02	640.83 ± 52.63
gepirone	10	83 ± 24; p<0.00001<br>F(1,10)=276.27; p<0.00001	5.50 ± 1.19; p<0.00001<br>F(1,10)=53.785; p<0.00001	0.67 ± 0.67;<br>p<0.00001<br>(F1,10)=166.14; p<0.00001	23.00 ± 6.32;<br>p<0.00001<br>F(1,10)=135.83

Compounds were investigated only in the active in the FST doses according to the 3R rules. Gepirone and **31** were given p.o. 60 min before the test. Values represent the mean ± SEM of the locomotor activity parameters during 5-min test session compared to the respective vehicle group (one-way ANOVA followed by Bonferroni's post hoc test); N=6

3. Detailed procedures for preparation of amine intermediates 8–24

The following compounds were previously reported or are commercially available:

2-[(6-methoxypyridin-2-yl)oxy]ethanamine (**8**) [1,2]

2-[(5-methoxypyridin-2-yl)oxy]ethanamine (**9**) [2]

2-[(4-methoxypyridin-2-yl)oxy]ethanamine (**10**)[2]

2-[(3-methoxypyridin-2-yl)oxy]ethanamine (**11**) [3]

2-[(6-fluoropyridin-2-yl)oxy]ethanamine (**12**) [2]

2-[(5-fluoropyridin-2-yl)oxy]ethanamine (**13**) [4]

2-[(4-fluoropyridin-2-yl)oxy]ethanamine (**14**) [4]

2-[(6-chloropyridin-2-yl)oxy]ethanamine (**15**)[5]

2-[(5-chloropyridin-2-yl)oxy]ethanamine (**16**)[6]

2-[[6-(Trifluoromethyl)-2-pyridinyl]oxy]ethanamine (**17**) [2,7]

2-[[5-(Trifluoromethyl)-2-pyridinyl]oxy]ethanamine (**18**)[1,8]

2-[(6-Methyl-2-pyridinyl)oxy]ethanamine (**22**) [6]

4.1. General procedure for preparation of amines 8–11, 13, 15–18, and 22.

To a solution of 2-aminoethanol (1.0–2.0 equiv) in dry 1,4-dioxane, NaH (1.0–2.0 equiv, 60% dispersion in mineral oil) was added and the reaction mixture was stirred at room temperature for 15 minutes. Then, the solution of the appropriate halogen derivative (1.0 equiv) in 1,4-dioxane was added dropwise. The mixture was stirred for 24–72 hours at room temperature or warmed to 80 °C, if necessary. After that time, the mixture was quenched with brine and extracted with EtOAc. The combined organic layers were dried over magnesium sulfate, filtered, and concentrated in vacuo to yield a crude product. Purification (if necessary) by flash chromatography yielded a pure product.

2-((6-Methoxypyridin-2-yl)oxy)ethanamine (8)

The title compound was prepared using 2-aminoethanol (2.0 equiv, 0.288 g, 4.72 mmol), NaH (2.0 equiv, 0.189 g, 4.72 mmol, 60% dispersion in mineral oil) and 2-fluoro-6-methoxypyridine (0.300 g, 2.36 mmol) in dry 1,4-dioxane (7 mL) at r.t. Time: 24 h. Purification: DCM/methanol/$NH_{3(aq)}$ (9.5/0.5/0.02, v/v/v). Yield: 31%; yellow oil. ^{1}H NMR (300 MHz, $CDCl_3$): δ 7.48 (t, *J* = 7.9 Hz, 1H), 6.30 (dd, *J* = 2.3, 7.6 Hz, 2H), 4.31 (t, *J* = 5.3 Hz, 2H), 3.88 (s, 3H), 3.07 (t, *J* = 5.3 Hz, 2H), 1.94 (br s, 2H). Formula: $C_8H_{12}N_2O_2$; MS (ESI$^+$): *m/z* 169 [M+H$^+$].

2-((5-Methoxypyridin-2-yl)oxy)ethanamine (9)

The title compound was prepared using 2-aminoethanol (2.0 equiv, 0.240 g, 3.94 mmol), NaH (2.0 equiv, 0.157 g, 3.94 mmol, 60% dispersion in mineral oil) and 2-fluoro-5-methoxypyridine (0.250 g, 1.97 mmol) in dry 1,4-dioxane (7 mL) at 80 °C. Time: 72 h. Purification: DCM/methanol/$NH_{3(aq)}$ (9/1/0.02, v/v/v). Yield: 31%; yellow transparent oil. ^{1}H NMR (300 MHz, $CDCl_3$): δ 7.77 (d, *J* = 2.9 Hz, 1H), 7.20 (dd, *J* = 3.2, 9.1 Hz, 1H), 6.69 (d, *J* = 8.8 Hz, 1H), 4.25 (t, *J* = 5.3 Hz, 2H), 3.80 (s, 3H), 3.05 (t, *J* = 5.3 Hz, 2H), 2.00 (s, 2H). Formula: $C_8H_{12}N_2O_2$; MS (ESI$^+$): *m/z* 169 [M+H$^+$].

2-((4-Methoxypyridin-2-yl)oxy)ethanamine (10)

The title compound was prepared using 2-aminoethanol (2.0 equiv, 0.288 g, 4.72 mmol), NaH (2.0 equiv, 0.189 g, 4.72 mmol, 60% dispersion in mineral oil) and 2-fluoro-4-methoxypyridine (0.300 g, 2.36 mmol) in dry 1,4-dioxane (7 mL) at r.t. Time: 24 h. Purification: DCM/methanol/$NH_{3(aq)}$ (9.5/0.5/0.02, v/v/v). Yield: 73%; beige solid. ^{1}H NMR (300 MHz, CD_3OD): δ 7.90 (d, *J* = 5.9 Hz, 1H), 6.58 (dd, *J* = 2.3, 5.9 Hz, 1H), 6.36 (d, *J* = 2.3 Hz, 1H), 4.30 (t, *J* = 5.3 Hz, 2H), 3.83 (s, 3H), 3.10–3.03 (m, 2H), NH_2 protons not detected. Formula: $C_8H_{12}N_2O_2$; MS (ESI$^+$): *m/z* 169 [M+H$^+$].

2-((3-Methoxypyridin-2-yl)oxy)ethanamine (11)

The title compound was prepared using 2-aminoethanol (1.0 equiv, 0.290 g, 4.76 mmol), NaH (1.0 equiv, 0.190 g, 4.76 mmol, 60% dispersion in mineral oil) and 2-bromo-3-methoxypyridine (0.890 g, 4.76 mmol) in dry 1,4-dioxane (10 mL). Temperature: 80 °C. Time: 72 h. Purification: EtOAc/methanol/$NH_{3(aq)}$ (8.5/1.5/0.02, v/v/v). Yield: 40%; orange crystallizing oil. ^{1}H NMR (300 MHz, $CDCl_3$) δ 7.69 (dd, *J* = 1.5, 5.1 Hz, 1H), 7.06-7.00 (m, 1H), 6.86-6.78 (m, 1H), 4.43-4.38 (m, 2H), 3.85 (s, 3H), 3.11 (t, *J* = 5.5 Hz, 2H), 1.56 (br s, 2H). Formula: $C_8H_{12}N_2O_2$; MS (ESI$^+$): *m/z* 169 [M+H$^+$].

2-((5-Fluoropyridin-2-yl)oxy)ethanamine (13)

The title compound was prepared using 2-aminoethanol (1.0 equiv, 0.265 g, 4.35 mmol), NaH (1.0 equiv, 0.174 g, 4.35 mmol, 60% dispersion in mineral oil) and 2,5-difluoropyridine (0.500 g, 4.35 mmol) in dry 1,4-dioxane (4 mL) at 80 °C. Time: 72 h. Purification: DCM/methanol/$NH_{3(aq)}$ (9/1/0.02, v/v/v). Yield: 87%; yellow solid. ^{1}H NMR (300 MHz, $CDCl_3$): δ 7.95 (d, *J* = 2.9 Hz, 1H), 7.32 (ddd, *J* = 2.9, 7.5, 8.9 Hz, 1H), 6.71 (dd, *J* = 3.5, 8.8 Hz, 1H), 4.30-4.21 (m, 2H), 3.08-3.00 (m, 2H), 1.59 (br s, 2H). Formula: $C_7H_9FN_2O$; MS (ESI$^+$): *m/z* 157 [M+H$^+$].

2-((6-Chloropyridin-2-yl)oxy)ethanamine (15)

The title compound was prepared using 2-aminoethanol (1.0 equiv, 0.124 g, 2.04 mmol), NaH (1.0 equiv, 0.082 g, 2.04 mmol, 60% dispersion in mineral oil) and 2,6-dichloropyridine (0.300 g, 2.04 mmol) in dry 1,4-dioxane (4 mL) at 80 °C. Time: 72 h. Purification: DCM/methanol/$NH_{3(aq)}$ (9.5/0.5/0.02, v/v/v). Yield: 30%; yellow crystallizing oil. ^{1}H NMR (300 MHz, $CDCl_3$): δ 7.52 (t, *J* = 7.9 Hz, 1H), 6.90 (d, *J* = 7.6 Hz, 1H), 6.67 (d, *J* = 8.2 Hz, 1H), 4.36-4.29 (m, 2H), 3.07 (t, *J* = 5.3 Hz, 2H), 2.03-1.99 (m, 2H). Formula: $C_7H_9ClN_2O$; MS (ESI$^+$): *m/z* 173 [M+H$^+$].

2-((5-Chloropyridin-2-yl)oxy)ethanamine (16)

The title compound was prepared using 2-aminoethanol (2.0 equiv, 0.664 g, 10.88 mmol), NaH (2.0 equiv, 0.435 g, 10.88 mmol, 60% dispersion in mineral oil) and 2,5-dichloropyridine (0.800 g, 5.44 mmol) in dry 1,4-dioxane (9 mL) at 80 °C. Time: 24 h. The compound was used in the next step without further purification. Yield: 78%; pale yellow solid. ^{1}H NMR (300 MHz, $CDCl_3$): δ 8.08 (d, *J* = 3.3 Hz, 1H), 7.52 (dd, *J* = 2.7, 8.8 Hz, 1H), 6.71 (d, *J* = 9.0 Hz, 1H), 4.33-4.26 (m, 2H), 3.10-3.03 (m, 2H), 1.42 (s, 2H). Formula: $C_7H_9ClN_2O$; MS (ESI$^+$): *m/z* 173 [M+H$^+$].

2-((6-(Trifluoromethyl)pyridin-2-yl)oxy)ethanamine (17)

The title compound was prepared using 2-aminoethanol (2.0 equiv, 0.222 g, 3.64 mmol), NaH (2.0 equiv, 0.145 g, 3.64 mmol, 60% dispersion in mineral oil) and 2-fluoro-6-(trifluoromethyl)pyridine (0.300 g, 1.82 mmol) in dry 1,4-dioxane (7 mL) at r.t. Time: 24 h. The compound was used in the next step without further purification. Yield: 76%; colorless oil. ^{1}H NMR (300 MHz, $CDCl_3$): δ 7.70 (t, *J* = 7.9 Hz, 1H), 7.29-7.21 (m, 1H), 6.93 (d, *J* = 8.2 Hz, 1H), 4.42-4.34 (m, 2H), 3.11-3.05 (m, 2H), 1.51 (s, 2H). Formula: $C_8H_9F_3N_2O$; MS (ESI$^+$): *m/z* 207 [M+H$^+$].

2-((4-(Trifluoromethyl)pyridin-2-yl)oxy)ethanamine (18)

The title compound was prepared using 2-aminoethanol (2.0 equiv, 0.419 g, 6.88 mmol), NaH (2.0 equiv, 0.275 g, 6.88 mmol, 60% dispersion in mineral oil) and 2-bromo-4-(trifluoromethyl)pyridine (0.770 g, 3.44 mmol) in dry 1,4-dioxane (6 mL) at 80 °C. Time: 24 h. The compound was used in the next step without further purification. Yield: 89%; yellow solid. ^{1}H NMR (300 MHz, $CDCl_3$): δ 8.29 (d, *J* = 5.4 Hz, 1H), 7.07 (d, *J* = 5.1 Hz, 1H), 6.99 (s, 1H), 4.41-4.34 (m, 2H), 3.09 (t, *J* = 5.4 Hz, 2H), 1.48 (s, 2H). Formula: $C_8H_9F_3N_2O$; MS (ESI$^+$): *m/z* 207 [M+H$^+$].

2-(6-Methylpyridin-2-yloxy)ethanamine (22)

The title compound was prepared using 2-aminoethanol (2.0 equiv, 0.330 g, 5.41 mmol), NaH (2.0 equiv, 0.216 g, 5.41 mmol, 60% dispersion in mineral oil) and 2-fluoro-6-methylpyridine (0.300 g, 2.70 mmol) in dry 1,4-dioxane (8 mL) at r.t. Time: 24 hs. The compound was used in the next step without further purification. Yield: 77%; white crystallizing oil. ^{1}H NMR (300 MHz, $CDCl_3$) δ 7.49-7.39 (m, 1H), 6.70 (d, *J* = 7.6 Hz, 1H), 6.52 (d, *J* = 8.2 Hz, 1H), 4.28 (t, *J* = 5.3 Hz, 2H), 3.08-3.01 (m, 2H), 2.41 (s, 3H), 1.64 (s, 2H). Formula: $C_8H_{12}N_2O$; MS (ESI$^+$): *m/z* 154 [M+H$^+$].

4.2. Synthesis of amine 12 (2-((6-fluoropyridin-2-yl)oxy)ethanamine hydrochloride)

Amine **12** was prepared in two-step synthesis according to the following procedure:

Step 1. *tert*-Butyl (2-((6-fluoropyridin-2-yl)oxy)ethyl)carbamate (**I 12**). To an ice-cooled solution of *tert*-butyl-2-hydroxyethyl carbamate (0.9 equiv, 3.780 g, 23.48 mmol) in THF (40 mL), NaH (0.9 equiv, 0.938 g, 23.48 mmol, 60% dispersion in mineral oil) was added. The resulting mixture was stirred at room temperature for 15 minutes and next added portionwise to an ice-cooled solution of 2,6-difluoropirydine (1.0 equiv, 3.000 g, 26.09 mmol) in THF (10 mL). Stirring was continued at room temperature for 3 h. Then, the mixture was cooled to 0 °C, quenched with a saturated aqueous solution of ammonium chloride, and extracted with EtOAc. The combined organics were dried over magnesium sulfate, filtered, and concentrated in vacuo to yield the crude product in 76% yield, as a white crystallizing oil (**I 12**). The product was used in the next step without further purification. ^{1}H NMR (300 MHz, $CDCl_3$): δ 7.65 (q, *J* = 8.2 Hz, 1H), 6.60 (dd, *J* = 1.7, 8.1 Hz, 1H), 6.47 (dd, *J* = 2.4, 7.8 Hz, 1H), 4.92 (br s, 1H), 4.36 - 4.28 (m, 2H), 3.51 (q, *J* = 5.2 Hz, 2H), 1.44 (s, 9 H). Formula: $C_{12}H_{17}FN_2O_3$; MS (ESI$^+$): *m/z* 201 [M - 56 (*tert*-butyl) +H$^+$].

Step 2. **2-((6-Fluoropyridin-2-yl)oxy)ethanamine hydrochloride** (**12**). The obtained crude product **I 12** (1.0 equiv, 0.171 g, 0.67 mmol) was mixed with 1.0 M solution of HCl in EtOAc (15 mL), and stirred at room temperature for 24 h. Then, the mixture was filtered giving the pure product as a hydrochloride salt. The compound was used in the next step without further purification. Yield: 92%; white solid. ^{1}H NMR (300 MHz, DMSO-d_6): δ 8.28 (br s, 3H), 7.96-7.86 (m, 1H), 6.76 (dt, *J* = 2.1, 7.6 Hz, 2H), 4.42-4.35 (m, 2H), 3.18 (t, *J* = 5.4 Hz, 2H). Formula: $C_7H_{10}ClFN_2O$; MS (ESI$^+$): *m/z* 157 [M+H$^+$]; Anal. Calcd for: C: 43.65; H: 5.23; N: 14.54. Found: C: 44.29; H: 4.80; N: 14.58.

4.3. Synthesis of amine 14 (2-((4-fluoropyridin-2-yl)oxy)ethanamine hydrochloride)

Amine **14** was prepared in two-step synthesis according to the following procedure:

Step 1. *tert*-Butyl (2-((4-fluoropyridin-2-yl)oxy)ethyl)carbamate (**I 14**). To the solution of *tert*-butyl-2-hydroxyethyl carbamate (1.0 equiv, 0.840 g, 5.22 mmol) and NaH (1.0 equiv, 0.209 g, 5.22 mmol, 60% dispersion in mineral oil) in dry 1,4-dioxane (4 mL) 2,4-difluoropyridine (1.0 equiv, 0.600 g, 5.22 mmol) was added dropwise. The reaction mixture was heated in a sealed tube at 90 °C for 20 min in the microwave reactor. After the mixture was cooled to room temperature, the solvent was removed. The obtained product was dissolved in brine and extracted with EtOAc. The combined organics were dried over magnesium sulfate, filtered, concentrated in vacuo, and purified by flash chromatography in *n*-hexane/Et_2O (5/5, v/v) to yield a pure product as a yellow crystallizing oil (30%). ^{1}H NMR (300 MHz, $CDCl_3$): δ 8.08 (dd, *J* = 5.9, 8.8 Hz, 1H), 6.66 (ddd, *J* = 2.3, 5.9, 8.2 Hz, 1H), 6.43 (dd, *J* = 1.8, 10.0 Hz, 1H), 5.06-4.88 (m, 1H), 4.37 (t, *J* = 5.3 Hz, 2H), 3.51 (m., 2H), 1.44 (s, 9H). Formula: $C_{12}H_{17}FN_2O_3$; MS (ESI$^+$): *m/z* 257 [M+H$^+$], 201 [M - 56 (*tert*-butyl) +H$^+$].

The reaction led to two isomers: *tert*-butyl (2-((4-fluoropyridin-2-yl)oxy)ethyl)carbamate and *tert*-butyl (2-((2-fluoropyridin-4-yl)oxy)ethyl)carbamate that could be distinguished based on their different retention times on HPLC, 6.11 min and 5.40 min, respectively.

Step 2. **2-((4-Fluoropyridin-2-yl)oxy)ethanamine hydrochloride (14)**. The obtained crude product **I 14** (1.0 equiv, 0.370 g, 1.45 mmol) was mixed with 1.0 M solution of HCl in EtOAc (25 mL) and stirred at room temperature for 24 h. Then, the mixture was filtered giving the pure product as a hydrochloride salt. The compound was used in the next step without further purification. Yield: 83%; white solid. ^{1}H NMR (300 MHz, CD_3OD): δ 8.33 (dd, *J* = 6.2, 7.9 Hz, 1H), 7.17-7.00 (m, 2H), 4.69-4.59 (m, 2H), 3.43 (t, *J* = 4.7 Hz, 2H), NH_3 protons not detected. ^{19}F NMR (282 MHz, CD_3OD): δ -94.1 (s, 1F). Formula:

$C_7H_{10}ClFN_2O$ + 1.5 H_2O; MS (ESI$^+$): *m/z* 157 [M+H$^+$]; Anal. Calcd for: C: 38.24; H: 5.91; N: 12.74. Found: C: 38.06; H: 4.97; N: 12.26.

4.4. General procedure for preparation of amines 19–21.

Amines **15–17** were prepared in two-step synthesis according to the following general procedure:

Step 1A. Nucleophilic aromatic substitution with amines. To 2,6-difluoropyridine (1.0 equiv) cooled to 0 °C in a sealed microwave tube 2.0 M solution of the appropriate amine in THF (1.5 equiv) was added dropwise. The reaction mixture was heated at 150 °C for 20 min in the microwave reactor. After the mixture was cooled to room temperature, it was quenched with water and extracted with EtOAc. The combined extracts were dried over magnesium sulfate, filtered, and concentrated to yield the product that was used in the next step without further purification (**I 19, I 20**).

Step 1B. Nucleophilic aromatic substitution with acetamide. To a solution of acetamide (1.0 equiv) and NaH (1.0 equiv, 60% dispersion in mineral oil) in DMF (3 mL), 2,6--difluoropyridine (1.0 equiv) was added dropwise. The reaction mixture was heated in a sealed tube at 100 °C for 20 min in the microwave reactor. After the mixture was cooled to room temperature, it was quenched with water and extracted with EtOAc. The combined organic layers were dried over magnesium sulfate, filtered, and concentrated in vacuo. The obtained product was purified by flash chromatography (**I 21**).

Step 2. Nucleophilic aromatic substitution with 2-aminoethanol. The obtained appropriate 6-fluoro-pyridin-2-amine derivative (1.0 equiv) was added dropwise to a mixture of 2-aminoethanol (2.0–3.0 equiv) and NaH (2.0–3.0 equiv, 60% dispersion in mineral oil) in dry 1,4-dioxane. The reaction mixture was heated in a sealed tube at 90 °C for 20 min in the microwave reactor. After the mixture was cooled to room temperature the solvent was removed. The obtained product was dissolved in brine and extracted with EtOAc. The combined organics were dried over magnesium sulfate filtered, concentrated in vacuo, and purified by flash chromatography.

6-(2-Aminoethoxy)-*N*-methylpyridin-2-amine (19)

Step 1A. 6-Fluoro-*N*-methylpyridin-2-amine (**I 19**) was prepared using 2,6--difluoropyridine (0.630 g, 5.47 mmol) and 2.0 M solution of methylamine in THF (4.11 mL, 8.21 mmol). The compound was used in the next step without further purification. Yield: 84%; green solid. ^{1}H NMR (300 MHz, $CDCl_3$): δ 7.53-7.42 (m, 1H), 6.18 (dd, *J* = 2.3, 8.2 Hz, 1H), 6.13 (dd, *J* = 2.3, 8.2 Hz, 1H), 4.77 (br s, 1H), 2.90 (d, *J* = 4.1 Hz, 3H). ^{19}F NMR (282 MHz, $CDCl_3$): δ -70.1 (s, 1F). Formula: $C_6H_7FN_2$; MS (ESI$^+$): *m/z* 127 [M+H$^+$].

Step 2. 6-(2-Aminoethoxy)-*N*-methylpyridin-2-amine (**19**) was prepared using 6-fluoro-*N*-methylpyridin-2-amine (**I 19**) (0.100 g, 0.79 mmol), 2-aminoethanol (0.144 g, 2.36 mmol) and NaH (0.094 g, 2.36 mmol) in dry 1,4-dioxane (2.5 mL). Purification: DCM/methanol/$NH_{3(aq)}$ (9/1/0.02, v/v/v). Yield: 81%; grey solid. ^1H NMR (300 MHz, $CDCl_3$): δ 7.40-7.31 (m, 1H), 6.02 (d, *J* = 8.2 Hz, 1H), 5.93 (d, *J* = 7.6 Hz, 1H), 4.41 (br s, 1H), 4.22 (t, *J* = 5.3 Hz, 2H), 3.03 (t, *J* = 5.3 Hz, 2H), 2.86 (br s, 3H), 1.69 (br s, 2H). Formula: $C_8H_{13}N_3O$; MS (ESI^+): *m/z* 168 $[M+H^+]$.

6-(2-Aminoethoxy)-*N*,*N*-dimethylpyridin-2-amine (20)

Step 1A. 6-Fluoro-*N*,*N*-dimethylpyridin-2-amine (**I 20**) was prepared using 2,6-difluoropyridine (0.630 g, 5.47 mmol) and 2.0 M solution of dimethylamine in THF (4.11 mL, 8.21 mmol). The compound was used in the next step without further purification. Yield: 70%; green oil. ^1H NMR (300 MHz, $CDCl_3$): δ 7.54-7.42 (m, 1H), 6.27 (dd, *J* = 2.3, 8.2 Hz, 1H), 6.08 (dd, *J* = 2.9, 7.6 Hz, 1H), 3.06 (s, 6H). ^{19}F NMR (282 MHz, $CDCl_3$): δ -69.0 (s, 1F). Formula: $C_7H_9FN_2$; MS (ESI^+): *m/z* 141 $[M+H^+]$.

Step 2. 6-(2-Aminoethoxy)-*N*,*N*-dimethylpyridin-2-amine (**20**) was prepared using 6-fluoro-*N*,*N*-dimethylpyridin-2-amine (**I 20**) (0.100 g, 0.71 mmol), 2-aminoethanol (0.087 g, 1.42 mmol) and NaH (0.057 g, 1.42 mmol) in dry 1,4-dioxane (2.5 mL). Purification: DCM/methanol/$NH_{3(aq)}$ (9/1/0.02, v/v/v). Yield: 65%; dark yellow oil. ^1H NMR (300 MHz, $CDCl_3$): δ 7.36 (t, *J* = 7.9 Hz, 1H), 6.01 (dd, *J* = 7.9, 9.1 Hz, 2H), 4.33-4.25 (m, 2H), 3.09-2.99 (m, 8H), 1.46 (s, 2H). Formula: $C_9H_{15}N_3O$; MS (ESI^+): *m/z* 182 $[M+H^+]$.

***N*-(6-(2-Aminoethoxy)pyridin-2-yl)acetamide (21)**

Step 1B. *N*-(6-Fluoropyridin-2-yl)acetamide (**I 21**) was prepared using 2,6-difluoropyridine (0.300 g, 2.61 mmol), acetamide (0.154 g, 2.61 mmol) and NaH (0.104 g, 2.61 mmol, 60% dispersion in mineral oil) in DMF (3 mL). Purification: *n*-hexane/DCM/methanol (2/7.8/0.2, v/v/v). Yield: 30%; yellow oil. ^1H NMR (300 MHz, $CDCl_3$): δ 8.06 (d, *J* = 8.2 Hz, 2H), 7.78 (q, *J* = 7.8 Hz, 1H), 6.65 (dd, *J* = 2.3, 7.6 Hz, 1H), 2.20 (s, 3H). Formula: $C_7H_7FN_2O$; MS (ESI^+): *m/z* 155 $[M+H^+]$.

Step 2. *N*-(6-(2-Aminoethoxy)pyridin-2-yl)acetamide (**21**) was prepared using *N*-(6-fluoropyridin-2-yl)acetamide (**I 21**) (0.352 g, 2.29 mmol), 2-aminoethanol (0.349 g, 5.71 mmol) and NaH (0.229 g, 5.71 mmol) in dry 1,4-dioxane (3.5 mL). The compound was used in the next step without further purification. Yield: 60%; pale yellow crystallizing oil. ^1H NMR (300 MHz, $CDCl_3$): δ 7.83 (br s, 1H), 7.72 (d, *J* = 7.0

Hz, 1H), 7.57 (t, J = 7.9 Hz, 1H), 6.47 (d, J = 8.8 Hz, 1H), 4.27-4.16 (m, 2H), 3.09-2.99 (m, 2H), 2.19 (s, 3H), 1.52 (s, 2H). Formula: $C_9H_{13}N_3O_2$; MS (ESI$^+$): *m/z* 196 [M+H$^+$].

4.5. Synthesis of amine 23 (2-((6-(pyrrolidin-1-yl)pyridin-2-yl)oxy)ethanamine hydrochloride.
Amine **23** was prepared in a three-step synthesis according to the following procedure:

Step 1. *tert*-Butyl(2-((6-(pyrrolidin-1-yl)pyridin-2-yl)oxy)ethyl)carbamate (**I 23**). To the obtained *tert*-butyl (2-((6-fluoropyridin-2-yl)oxy)ethyl)carbamate (**I 12** – see section 4.2) (1.0 equiv, 0.300 g, 1.17 mmol) pyrrolidine (4.6 equiv, 0.383 g, 5.39 mmol) was added and stirred at 80 °C for 3 h. Then, the mixture was warmed to room temperature and stirred for 48 h. After that time, the reaction mixture was diluted with a 5% aqueous solution of HCl, aqueous was extracted with EtOAc, organics dried over magnesium sulfate, filtered, and concentrated in vacuo. The crude product was purified by flash chromatography (*n*-hexane/EtOAc, 9/1, v/v) to yield the pure product in 47% yield as a pale yellow crystallizing oil. ^{1}H NMR (300 MHz, CD_3OD): δ 7.34 (t, J = 8.0 Hz, 1H), 5.93 (d, J = 9.7 Hz, 2H), 4.26 (t, J = 5.6 Hz, 2H), 3.44-3.35 (m, 6H), 2.02-1.94 (m, 4H), 1.42 (s, 9H), N*H* proton not detected. Formula: $C_{16}H_{25}N_3O_3$; MS (ESI$^+$): *m/z* 308 [M+H$^+$].

Step 2. **2-((6-(Pyrrolidin-1-yl)pyridin-2-yl)oxy)ethanamine hydrochloride (23).** The obtained product **I 23** (1.0 equiv, 0.170 g, 0.55 mmol) was mixed with a 1.0 M solution of HCl in EtOAc (15 mL) and stirred at room temperature for 24 h. Then, the mixture was filtered giving the pure product as a hydrochloride salt. The compound was used in the next step without further purification. Yield: 93%; white solid. ^{1}H NMR (300 MHz, DMSO-d_6): δ 8.25 (br s, 3H), 7.43 (t, J = 8.0 Hz, 1H), 5.98 (t, J = 8.3 Hz, 2H), 4.38 (t, J = 5.4 Hz, 2H), 3.41-3.29 (m, 4H), 3.20-3.09 (m, 2H), 1.97-1.85 (m, 4H). Formula: $C_{11}H_{18}ClN_3O$; MS (ESI$^+$): *m/z* 208 [M+H$^+$].

4.6. Synthesis of amine 24 (2-((6-(1H-pyrazol-1-yl)pyridin-2-yl)oxy)ethanamine).
Amine **24** was prepared in a three-step synthesis according to the following procedure:

Step 1. *tert*-Butyl (2-((6-(1*H*-pyrazol-1-yl)pyridin-2-yl)oxy)ethyl)carbamate (**I 24**)

To the suspension of NaH (3.0 equiv, 0.047 g, 1.17 mmol, 60% dispersion in mineral oil) in dry DMF (0.5 mL), pyrazole (3.0 equiv, 0.080 g, 1.17 mmol) dissolved in DMF (0.5 mL) was added dropwise and stirred at room temperature for 2 h. Then, the obtained *tert*-butyl (2-((6-fluoropyridin-2-yl)oxy)ethyl)carbamate (**I 12**) (1.0 equiv, 0.100 g, 0.39 mmol) in DMF (0.5 mL) was added dropwise and stirred for 3 h at 80 °C. The mixture was diluted with cold water and extracted with EtOAc. The combined organic

fractions were dried over magnesium sulfate, filtered, and concentrated in vacuo. The crude product was purified by flash chromatography (*n*-hexane/EtOAc, 8/2, v/v) to yield the pure product in 51% yield, as a beige solid. ^{1}H NMR (300 MHz, $CDCl_3$): δ 8.48 (d, *J* = 2.6 Hz, 1H), 7.74-7.68 (m, 1H), 7.62 (d, *J* = 2.1 Hz, 1H), 7.53 (d, *J* = 8.5 Hz, 1H), 6.63 (dd, *J* = 0.6, 8.1 Hz, 1H), 6.44 (dd, *J* = 1.5, 2.6 Hz, 1H), 5.09-4.96 (m, 1H), 4.42 (t, *J* = 5.3 Hz, 2H), 3.57 (q, *J* = 5.5 Hz, 2H), 1.45 (s, 9H). Formula: $C_{15}H_{20}N_4O_3$; MS (ESI^+): *m/z* 305 [$M+H^+$].

Step 2. **2-((6-(1*H*-Pyrazol-1-yl)pyridin-2-yl)oxy)ethanamine (24).** The obtained product **I 24** (1.0 equiv, 0.243 g, 0.80 mmol) was mixed with a 1.0 M solution of HCl in EtOAc (25 mL) and stirred at room temperature for 24 h. Then, the mixture was filtered giving the pure product as a hydrochloride salt. The obtained hydrochloride salt was turned into the free base (using a 10% aqueous solution of sodium carbonate) before purification. Purification: DCM/methanol/$NH_{3(aq)}$ (9/1/0.02, v/v/v). Yield: 68%; white solid. ^{1}H NMR (300 MHz, CD_3OD): δ 8.60 (d, *J* = 2.1 Hz, 1H), 7.87 (t, *J* = 8.0 Hz, 1H), 7.77 (d, *J* = 1.0 Hz, 1H), 7.52 (d, *J* = 7.7 Hz, 1H), 6.83 (d, *J* = 8.2 Hz, 1H), 6.55 (dd, *J* = 1.7, 2.7 Hz, 1H), 4.67-4.62 (m, 2H), 3.45-3.38 (m, 2H), NH_2 protons not detected. Formula: $C_{10}H_{12}N_4O$; MS (ESI^+): *m/z* 205 [$M+H^+$].

4. Spectra of the target compounds (^{1}H NMR, ^{19}F NMR, ^{13}C NMR, LC-MS; Figures S1–S56).

Characterizations of compound 25.

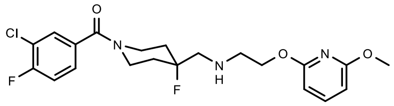

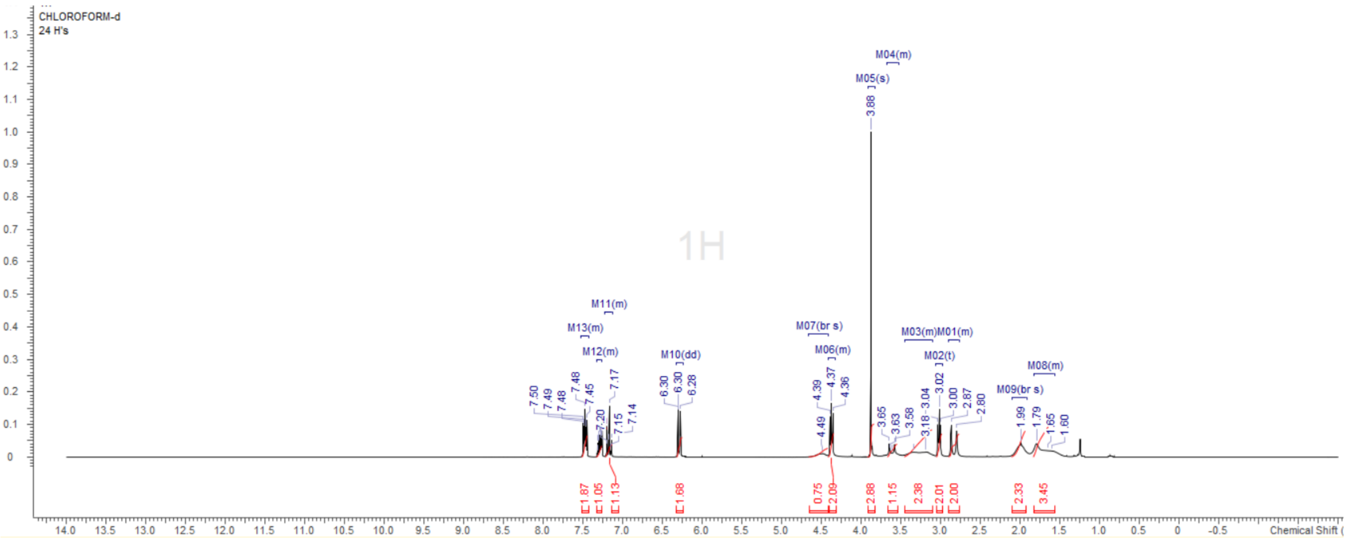

Figure S1. ^{1}H NMR (300 MHz, $CDCl_3$) spectrum of compound 25.

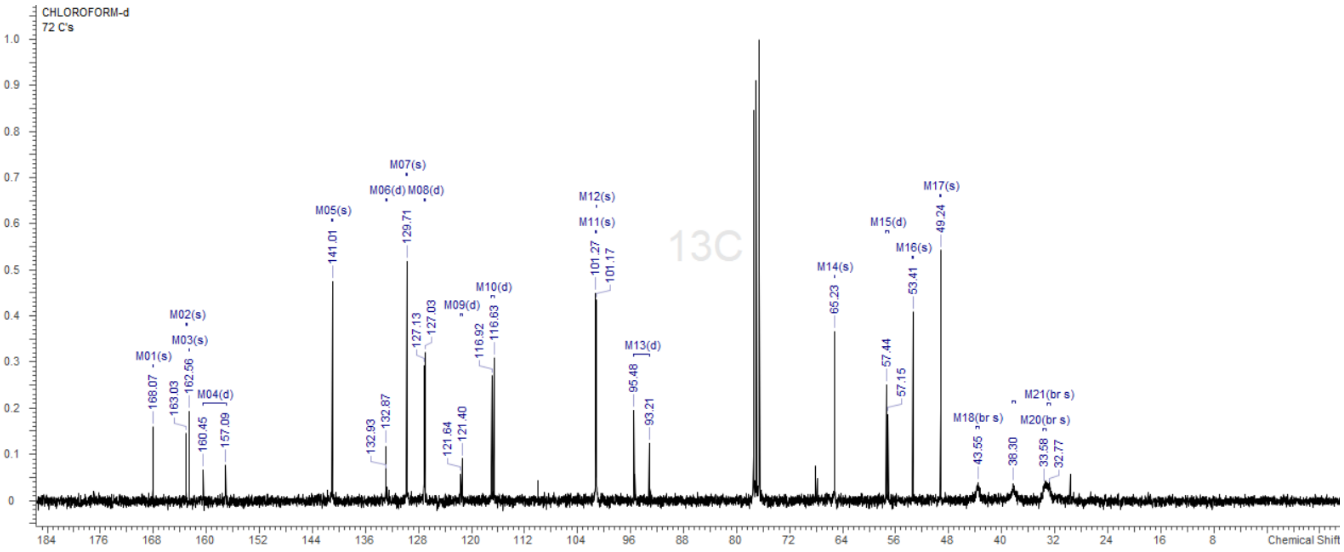

Figure S2. ^{13}C NMR (75 MHz, $CDCl_3$) spectrum of compound 25.

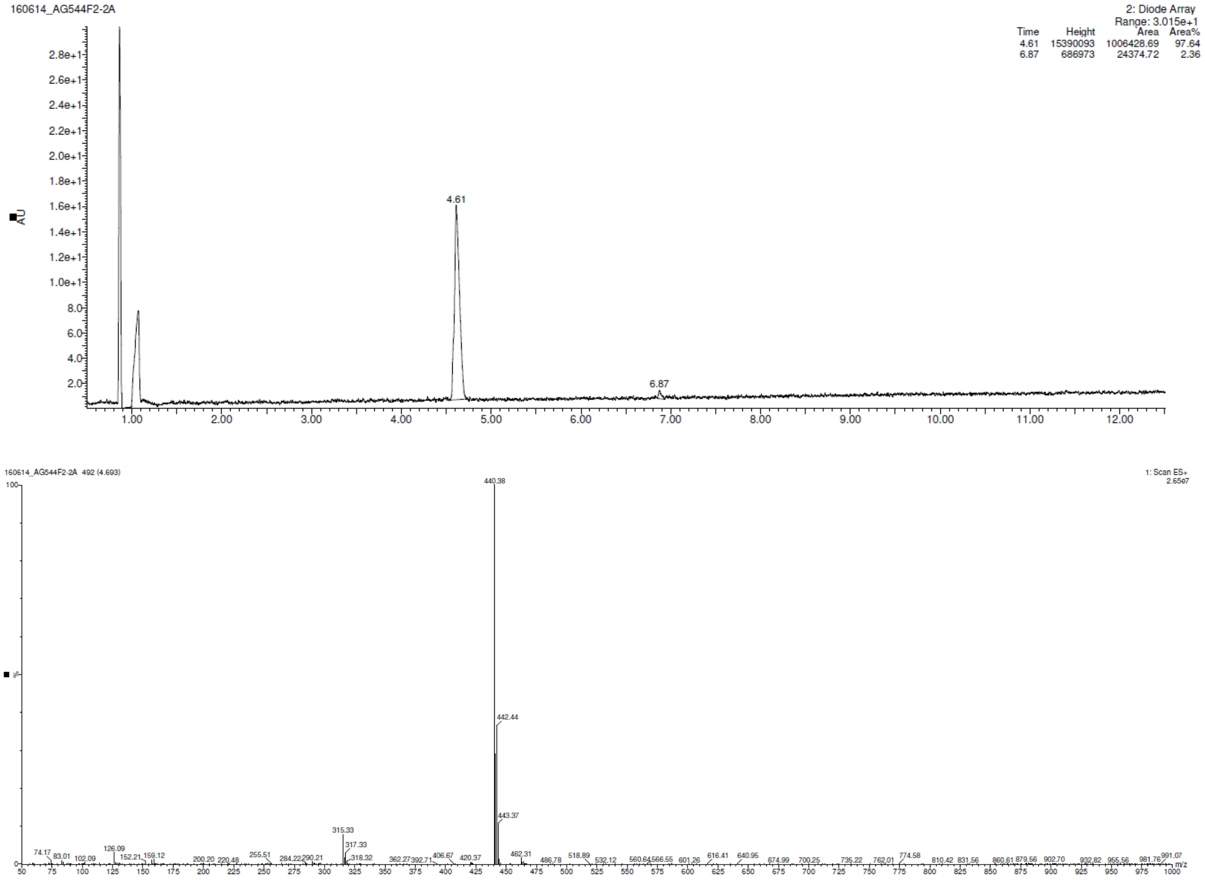

Figure S3. LC-MS chromatograms of compound 25 (97.64%).

Characterizations of compound 26.

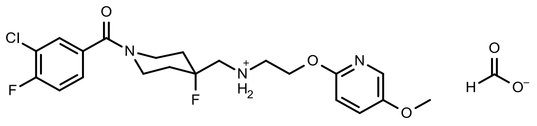

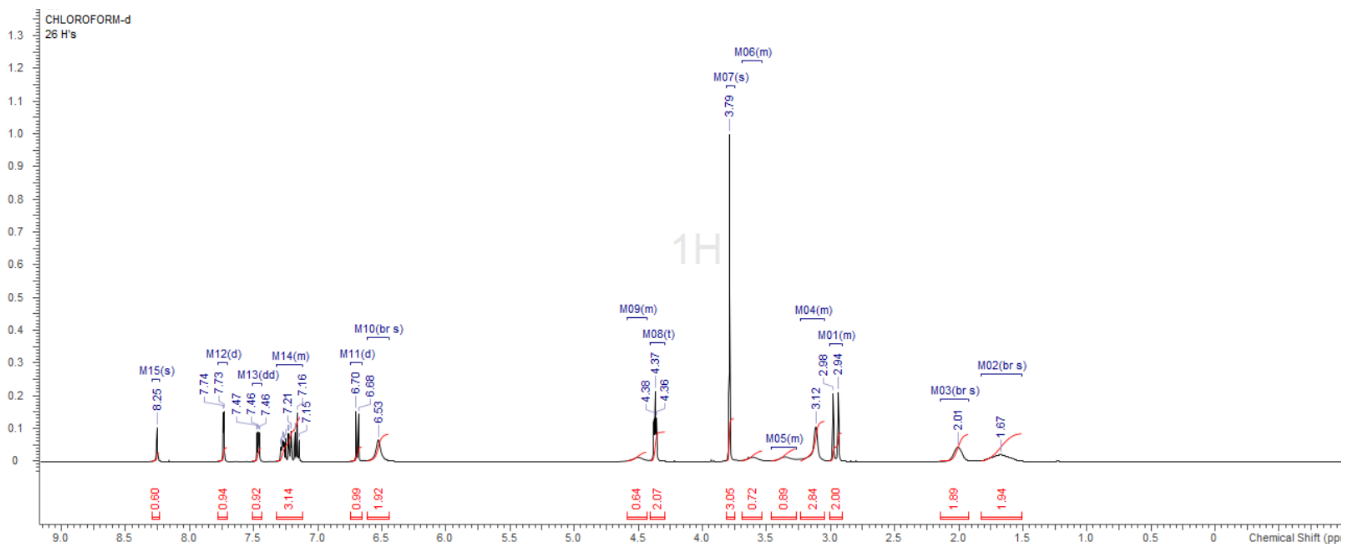

Figure S4. ^{1}H NMR (500 MHz, $CDCl_3$) spectrum of compound 26.

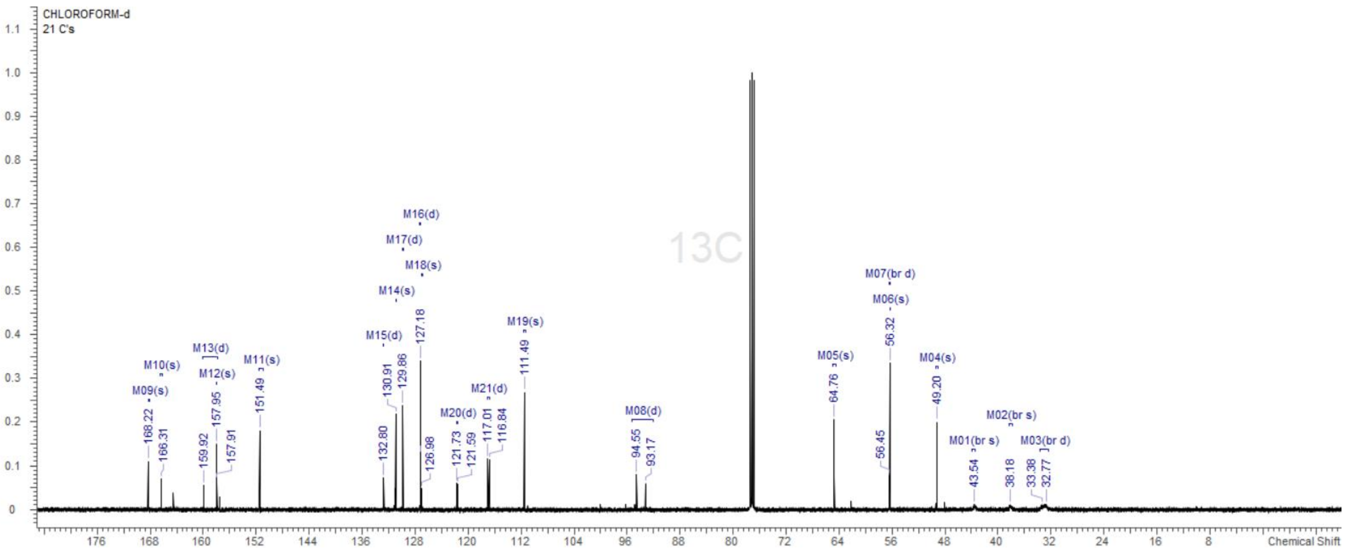

Figure S5. ^{13}C NMR (126 MHz, $CDCl_3$) spectrum of compound 26.

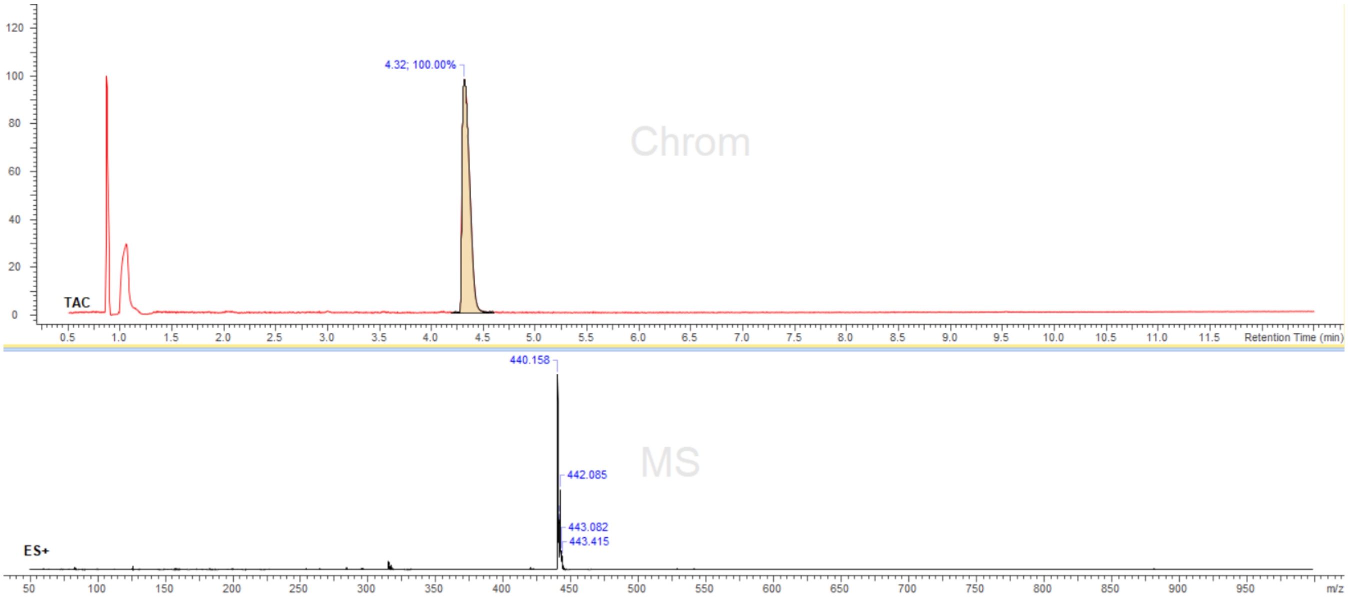

Figure S6. LC-MS chromatograms of compound 26 (100.00%).

Characterizations of compound 27.

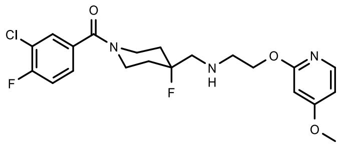

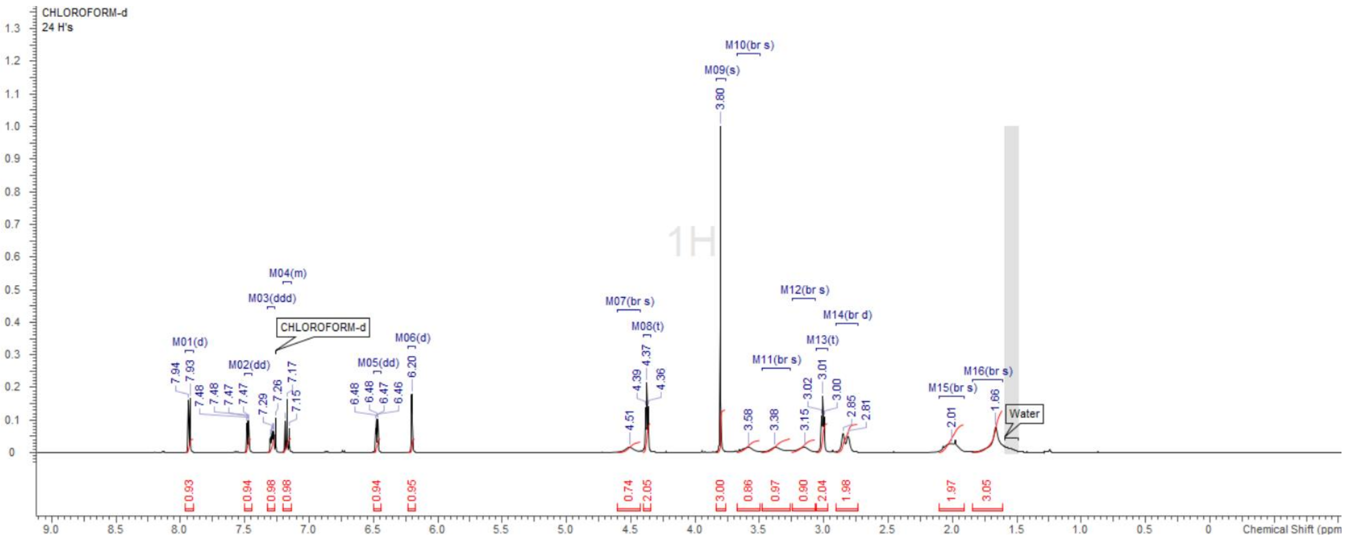

Figure S7. ^{1}H NMR (500 MHz, $CDCl_3$) spectrum of compound 27.

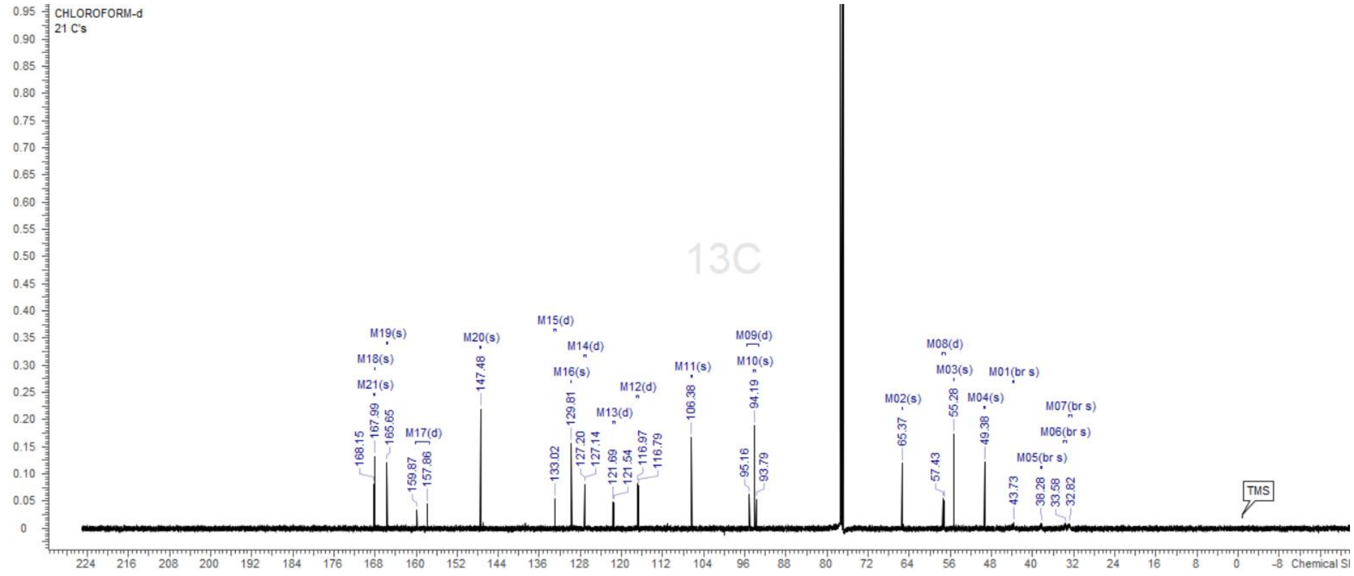

Figure S8. ^{13}C NMR (126 MHz, $CDCl_3$) spectrum of compound 27.

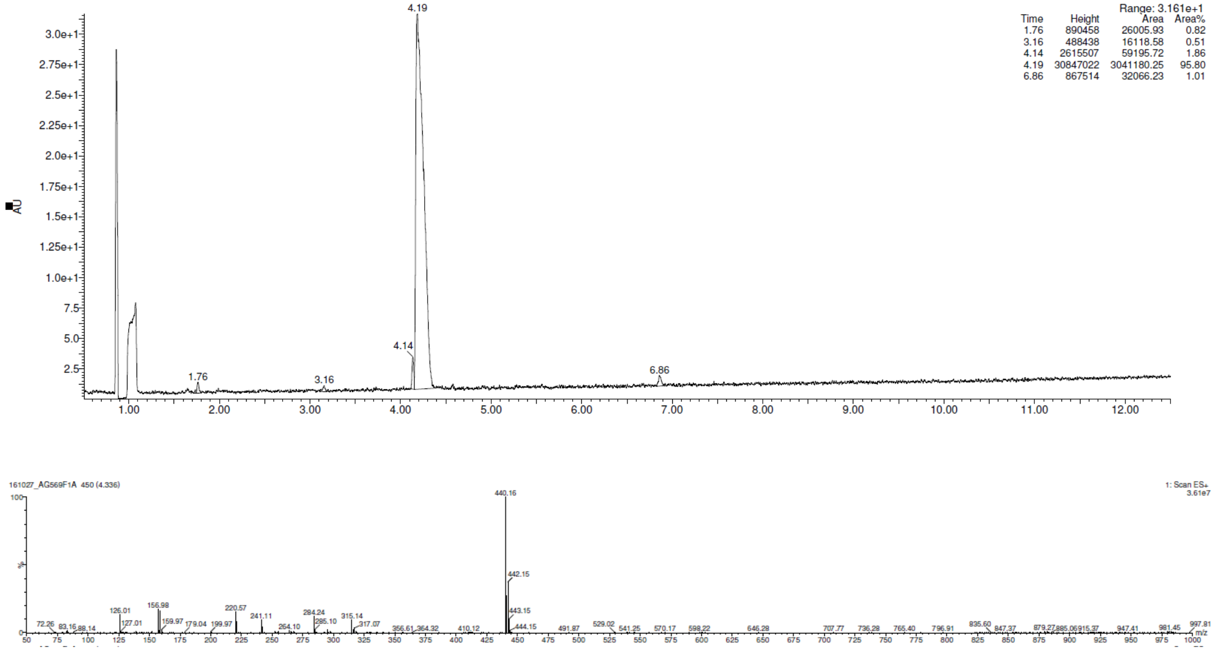

Figure S9. LC-MS chromatograms of compound 27 (95.80%).

Characterizations of compound 28.

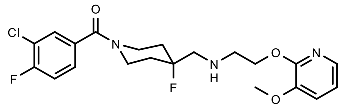

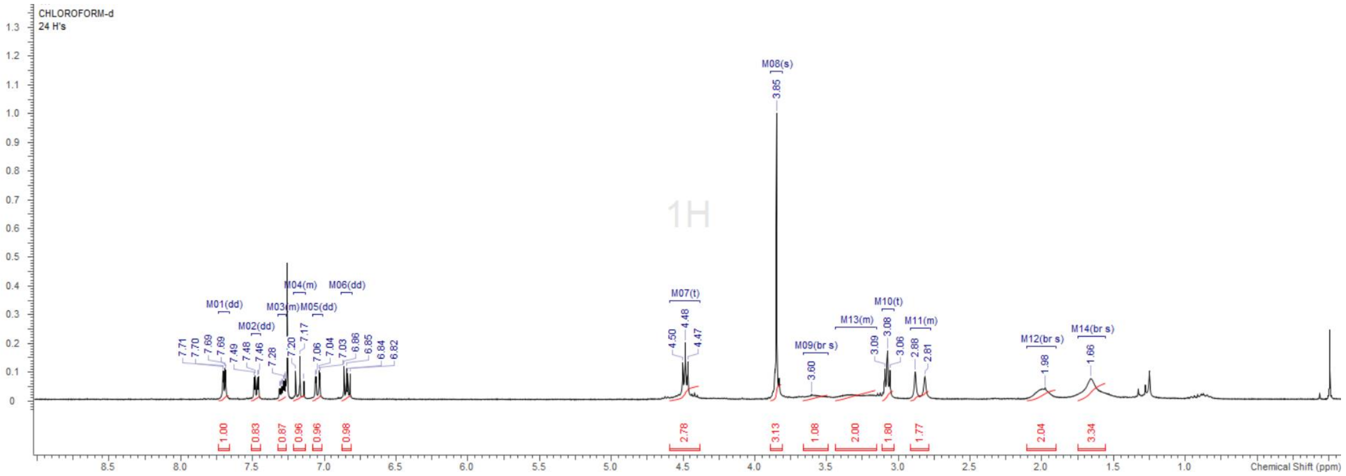

Figure S10. ^{1}H NMR (300 MHz, $CDCl_3$) spectrum of compound 28.

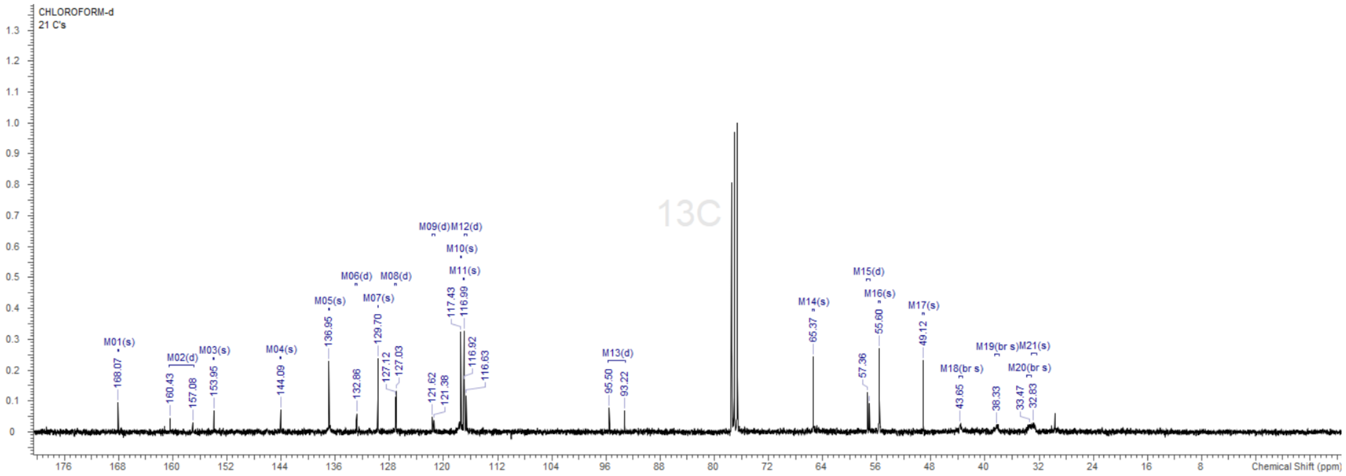

Figure S11. ^{13}C NMR (75 MHz, $CDCl_3$) spectrum of compound 28.

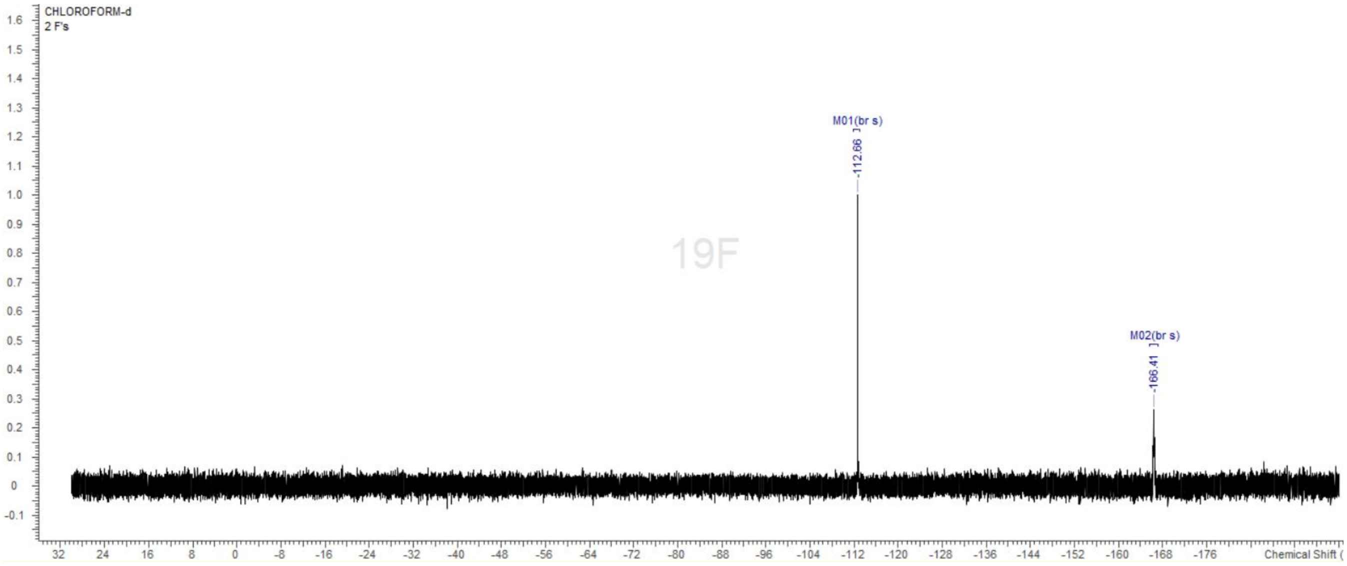

Figure S12. ^{19}F NMR (282 MHz, $CDCl_3$) spectrum of compound 28.

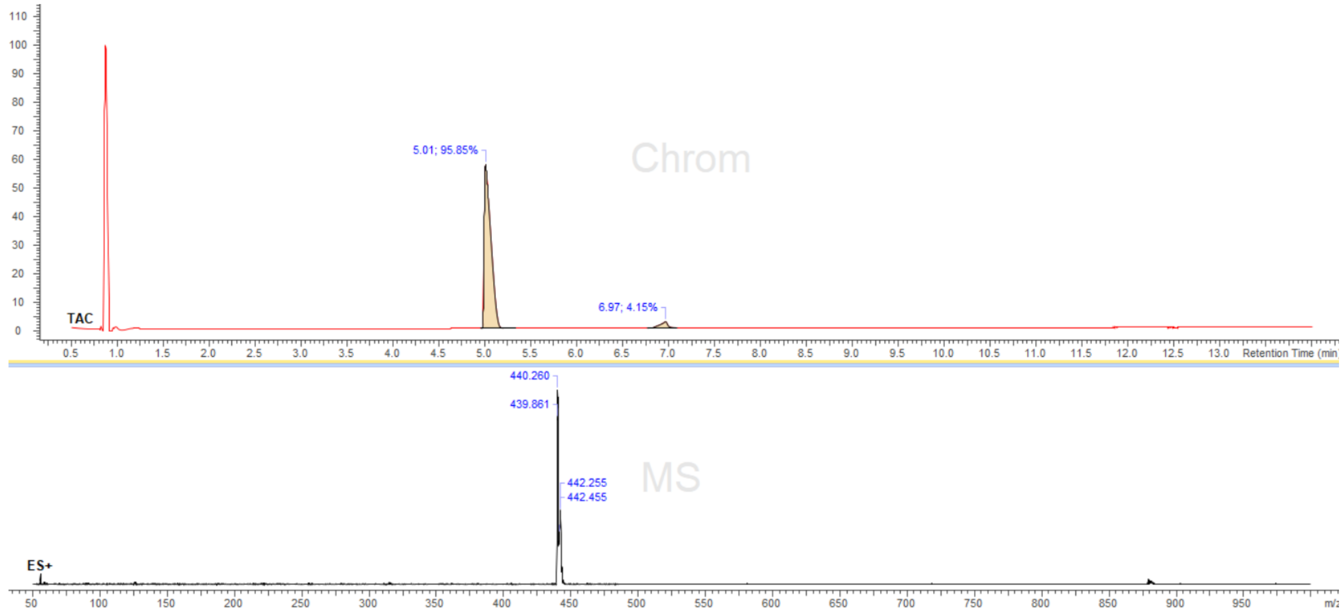

Figure S13. LC-MS chromatograms of compound 28 (95.85%).

Characterizations of compound 29.

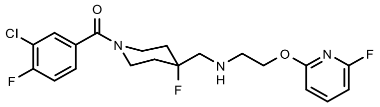

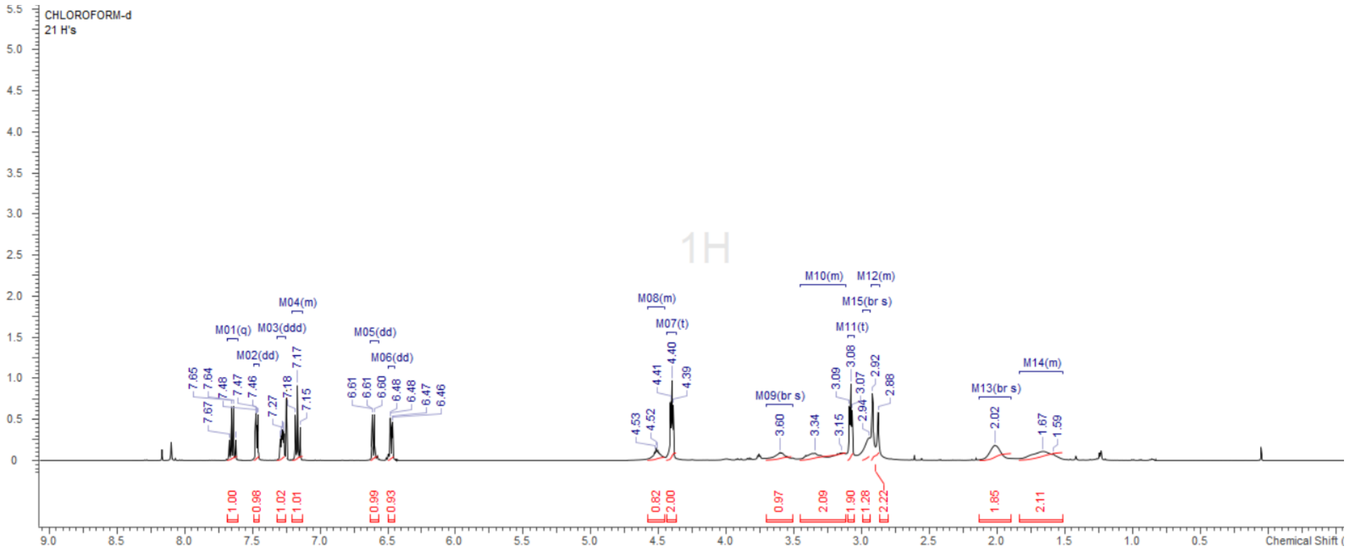

Figure S14. ^{1}H NMR (500 MHz, $CDCl_3$) spectrum of compound 29.

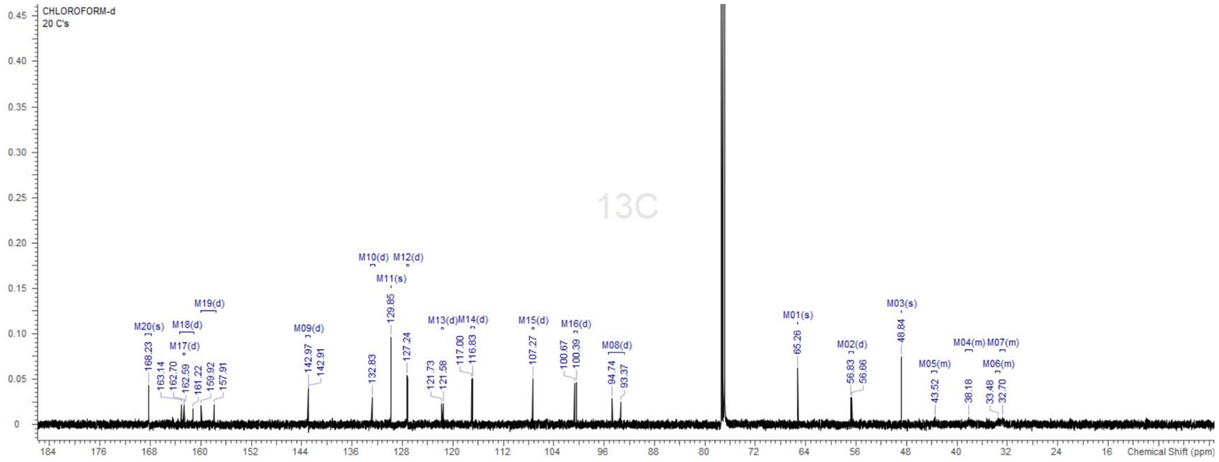

Figure S15. ^{13}C NMR (126 MHz, $CDCl_3$) spectrum of compound 29.

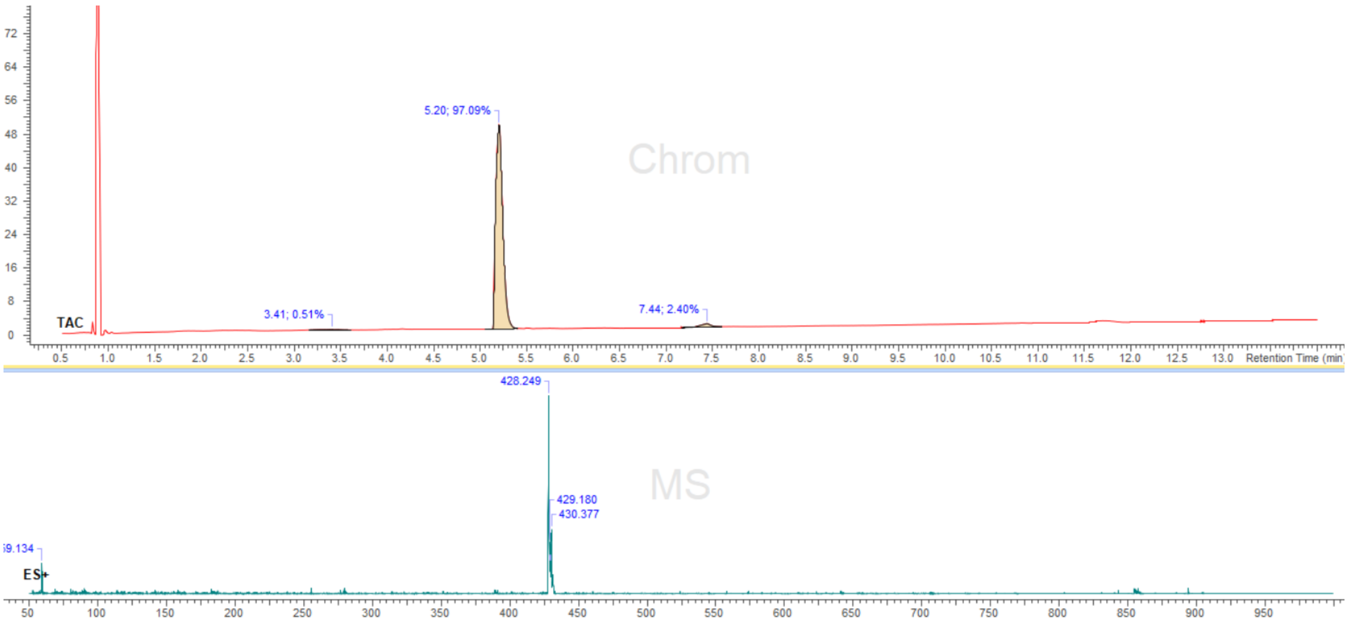

Figure S16. LC-MS chromatograms of compound 29 (97.09%).

Characterizations of compound 30.

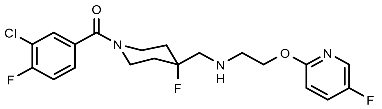

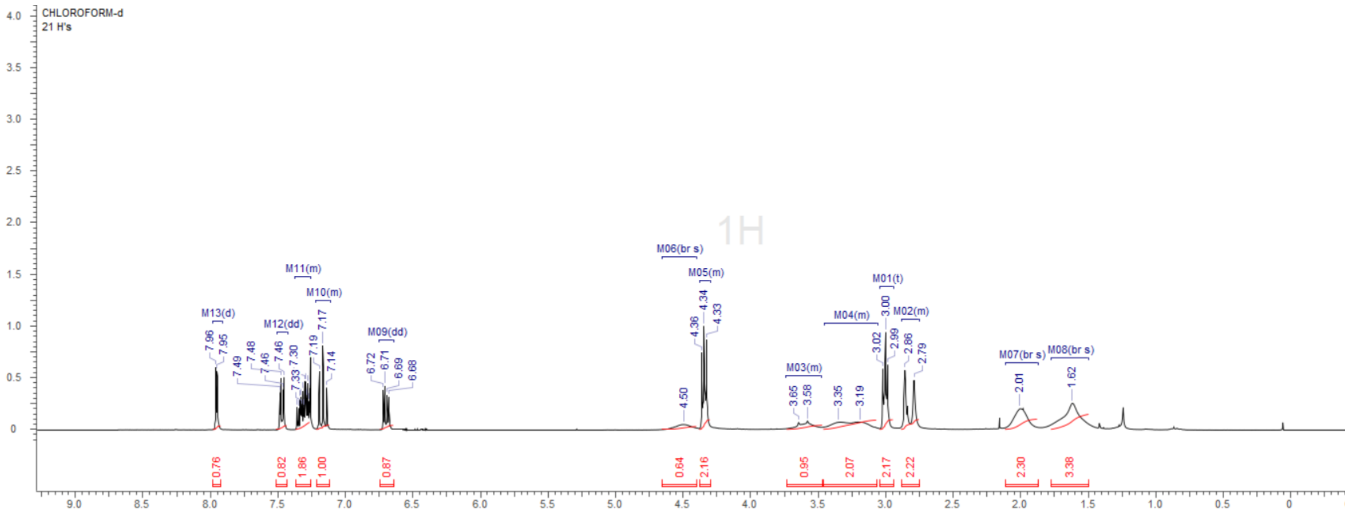

Figure S17. ^{1}H NMR (300 MHz, $CDCl_3$) spectrum of compound 30.

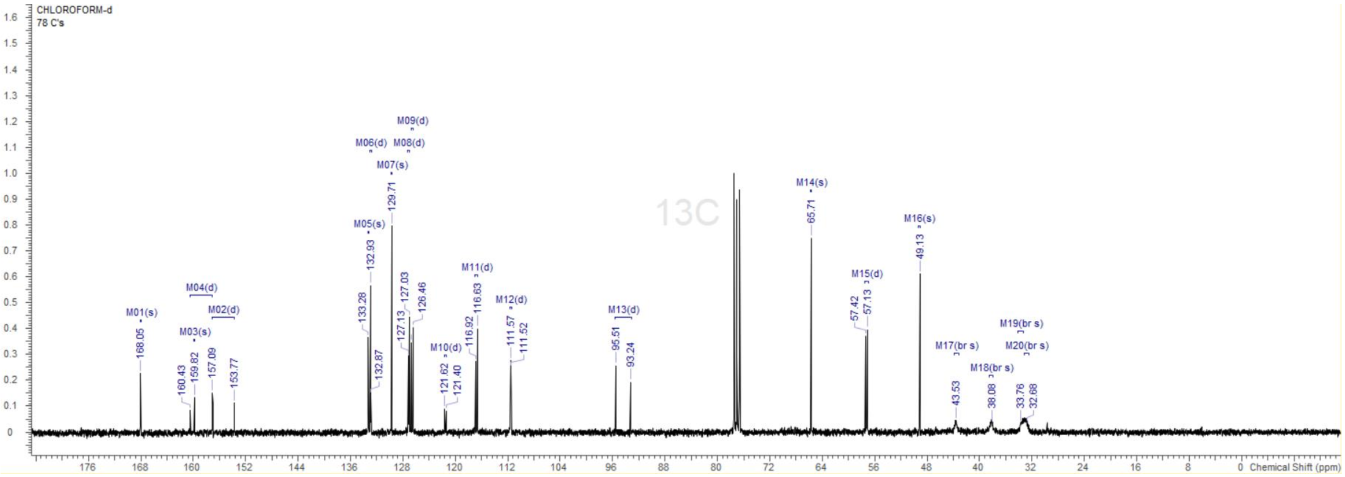

Figure S18. ^{13}C NMR (75 MHz, $CDCl_3$) spectrum of compound 30.

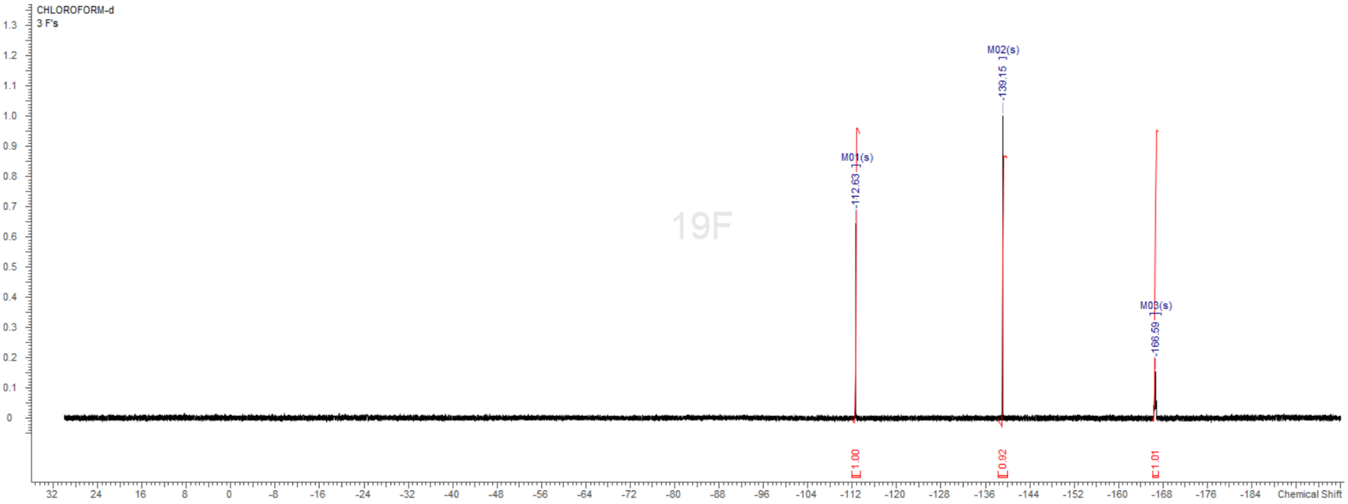

Figure S19. ^{19}F NMR (282 MHz, $CDCl_3$) spectrum of compound 30.

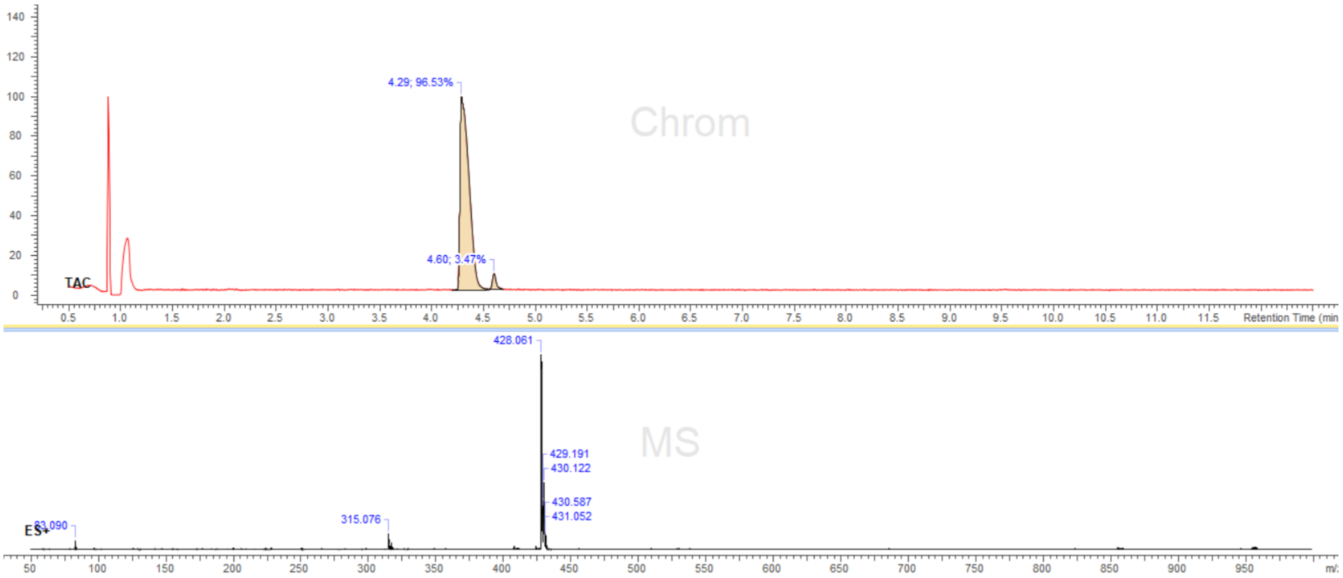

Figure S20. LC-MS chromatograms of compound 30 (96.53%).

Characterizations of compound 31.

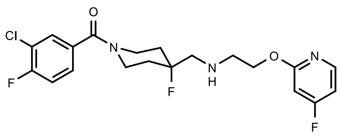

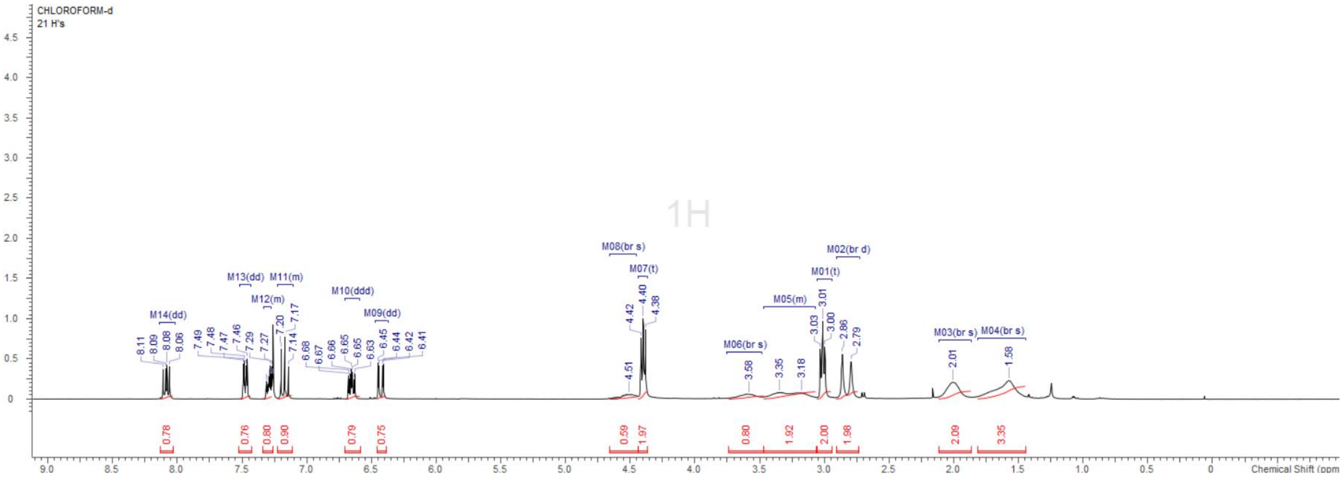

Figure S21. ^{1}H NMR (300 MHz, $CDCl_3$) spectrum of compound 31.

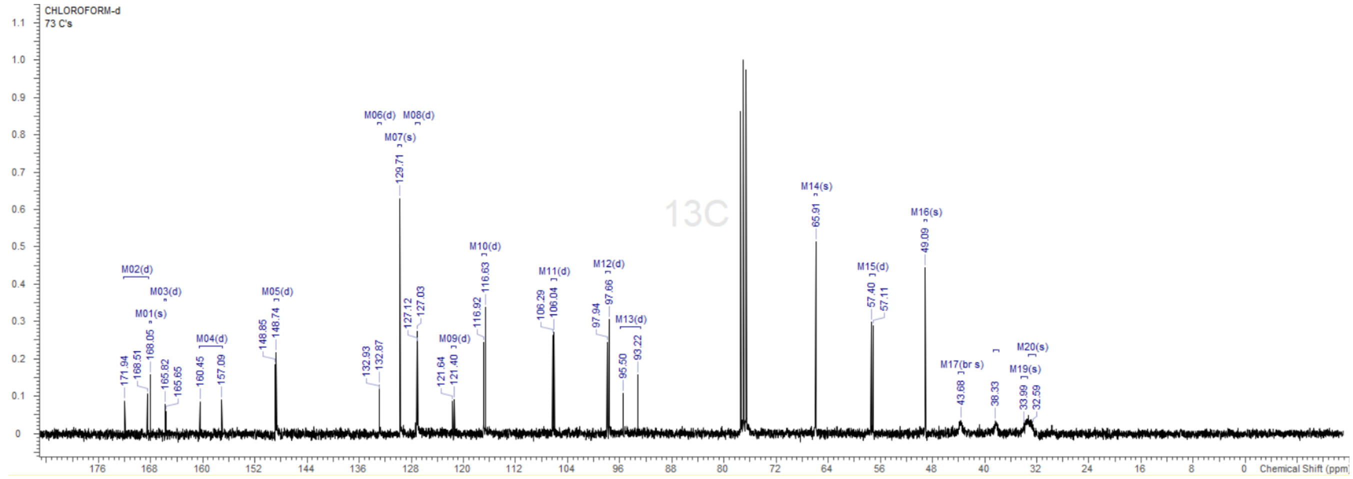

Figure S22. ^{13}C NMR (75 MHz, $CDCl_3$) spectrum of compound 31.

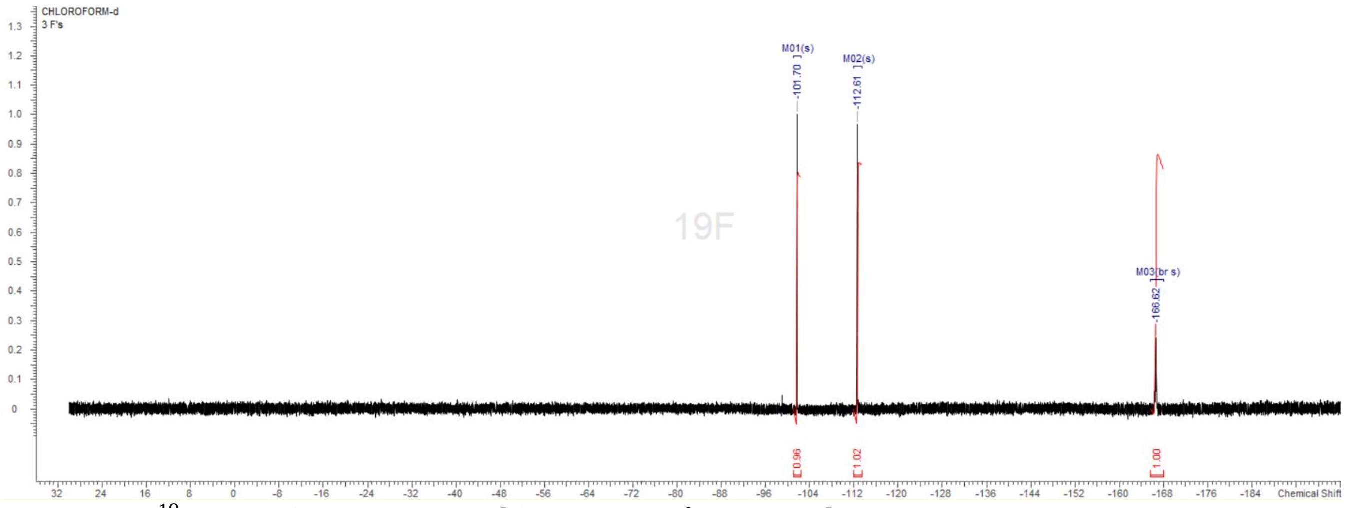

Figure S23. ^{19}F NMR (282 MHz, $CDCl_3$) spectrum of compound 31**.**

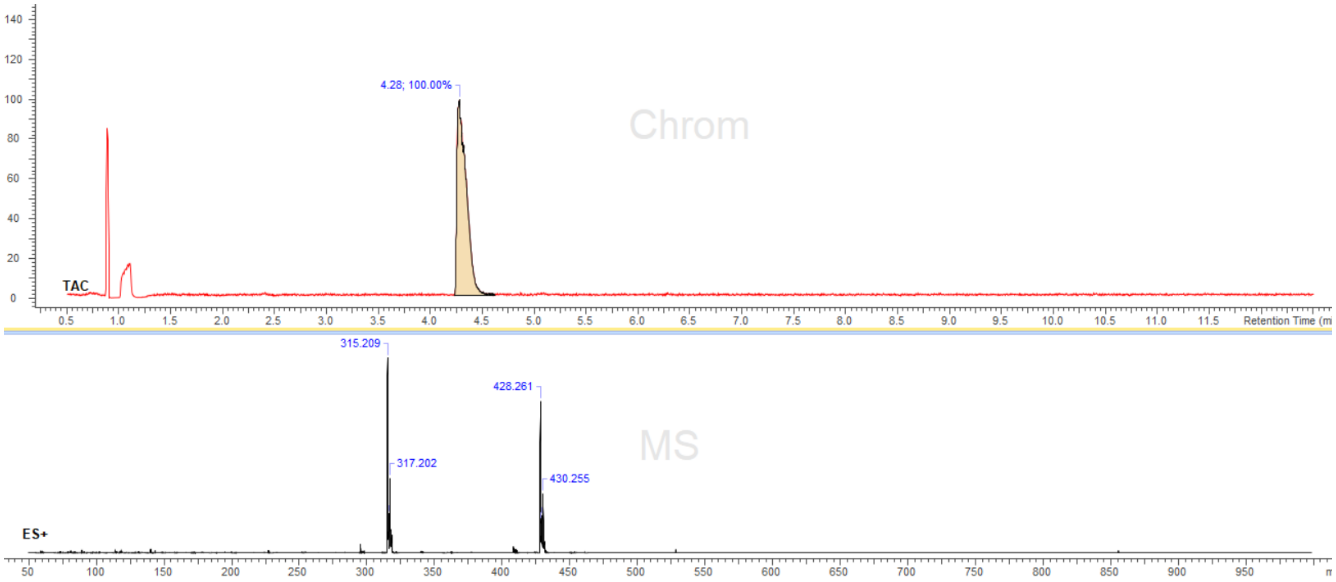

Figure S24. HPLC spectra of compound 31 (100.00%).

Characterizations of compound 32.

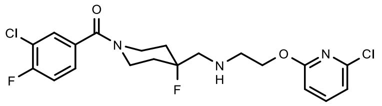

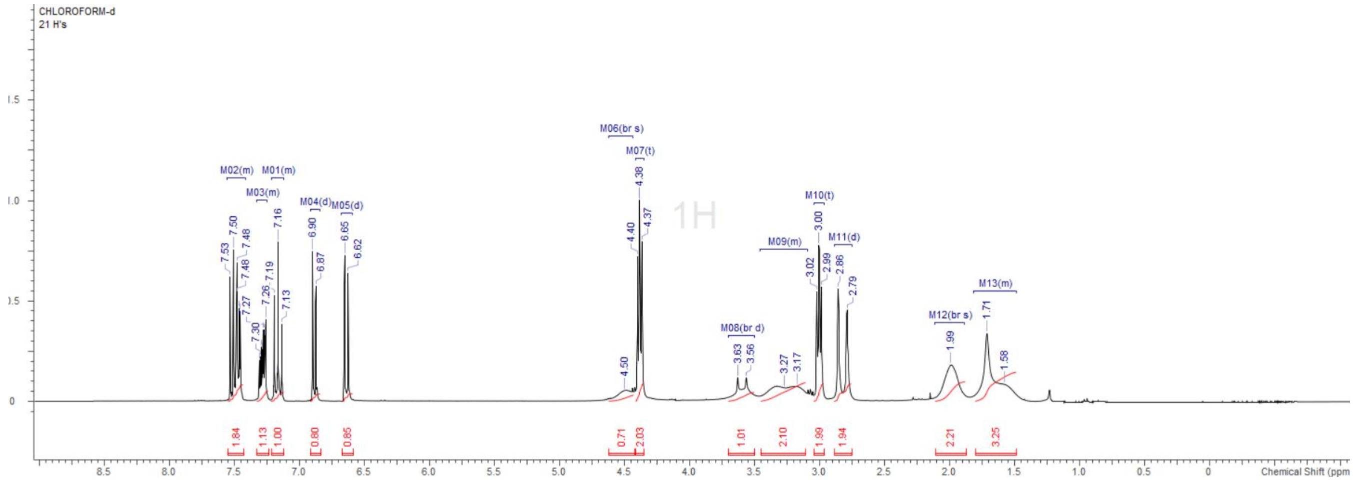

Figure S25. ^{1}H NMR (300 MHz, $CDCl_3$) spectrum of compound 32.

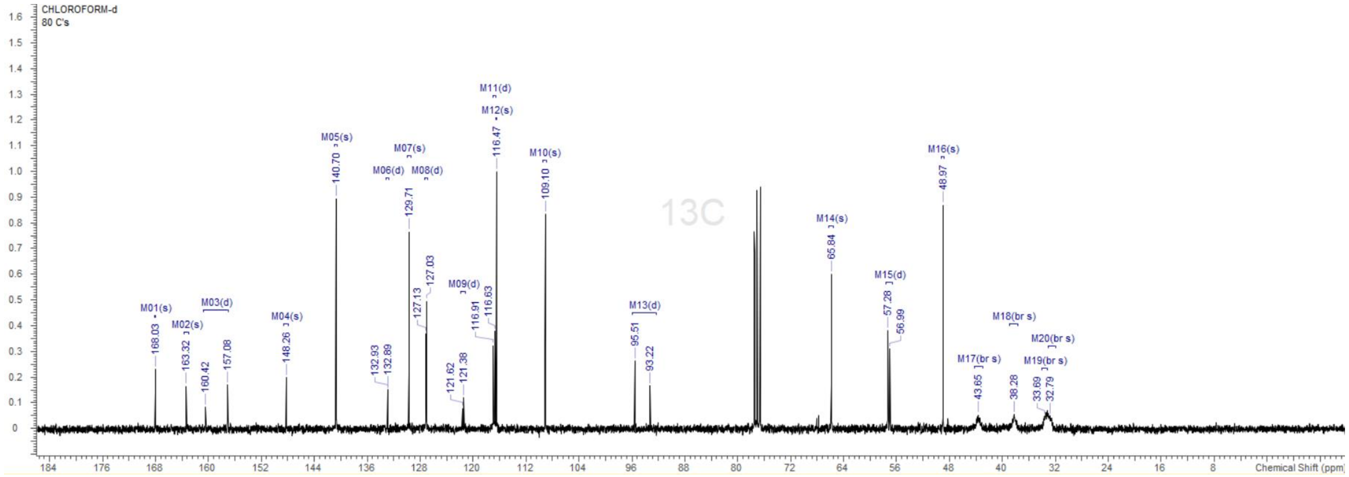

Figure S26. ^{13}C NMR (75 MHz, $CDCl_3$) spectrum of compound 32.

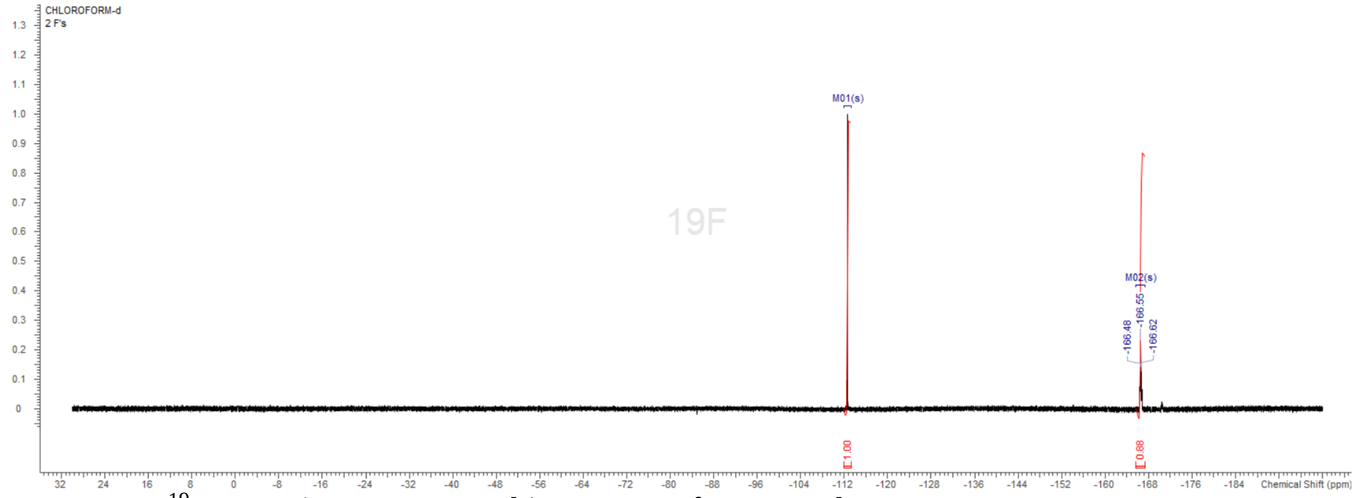

Figure S27. ^{19}F NMR (282 MHz, $CDCl_3$) spectrum of compound 32.

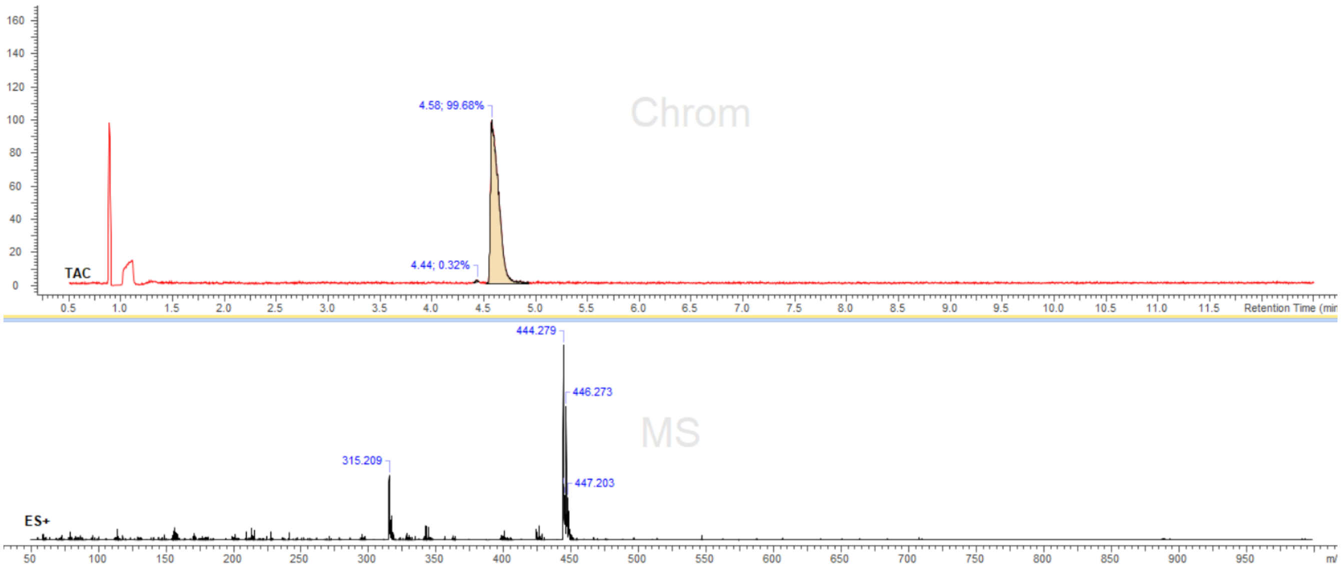

Figure S28. LC-MS chromatograms of compound 32 (99.68%).

Characterizations of compound 33.

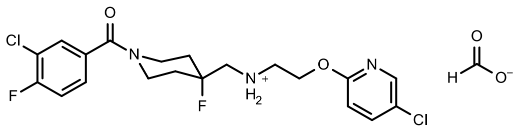

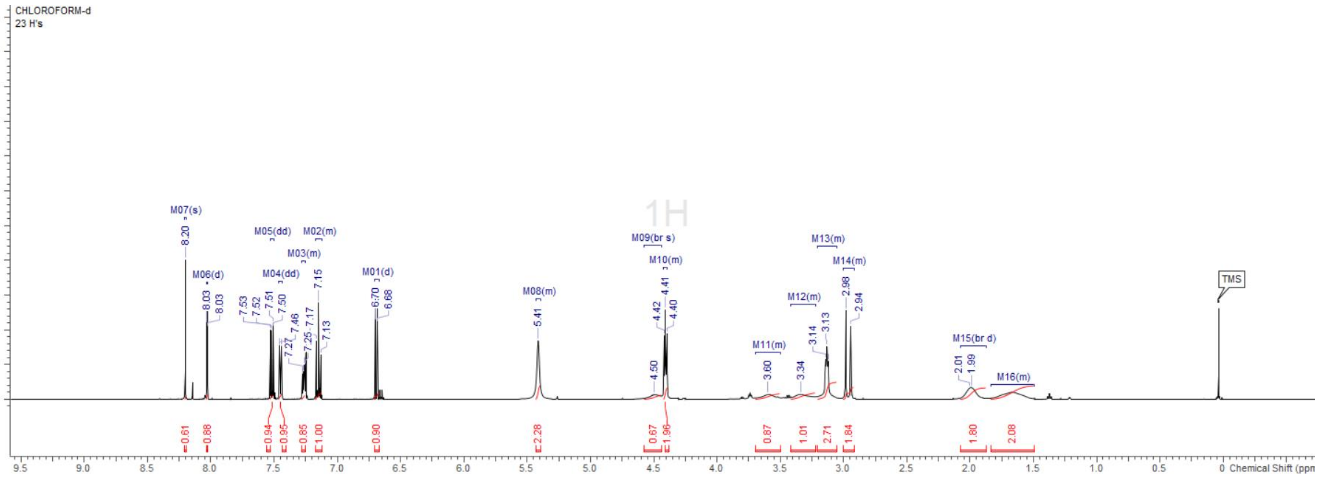

Figure S29. ^{1}H NMR (500 MHz, $CDCl_3$) spectrum of compound 33.

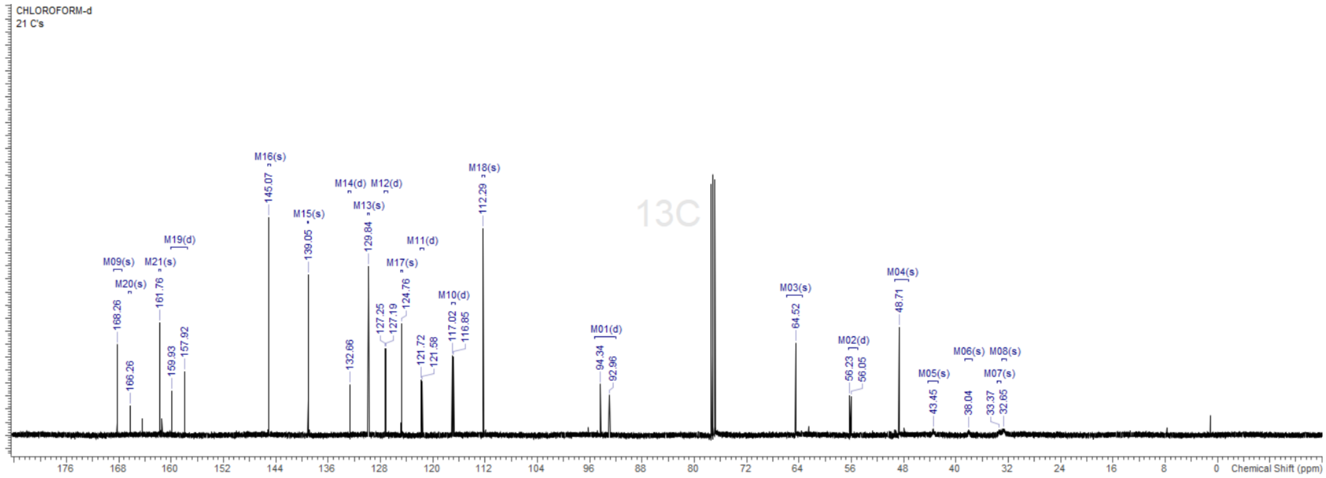

Figure S30. ^{13}C NMR (126 MHz, $CDCl_3$) spectrum of compound 33.

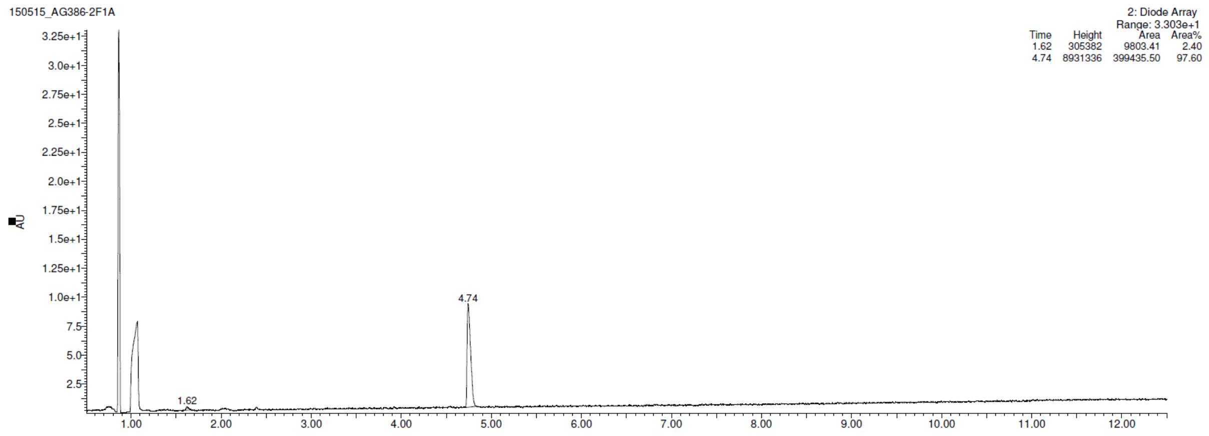

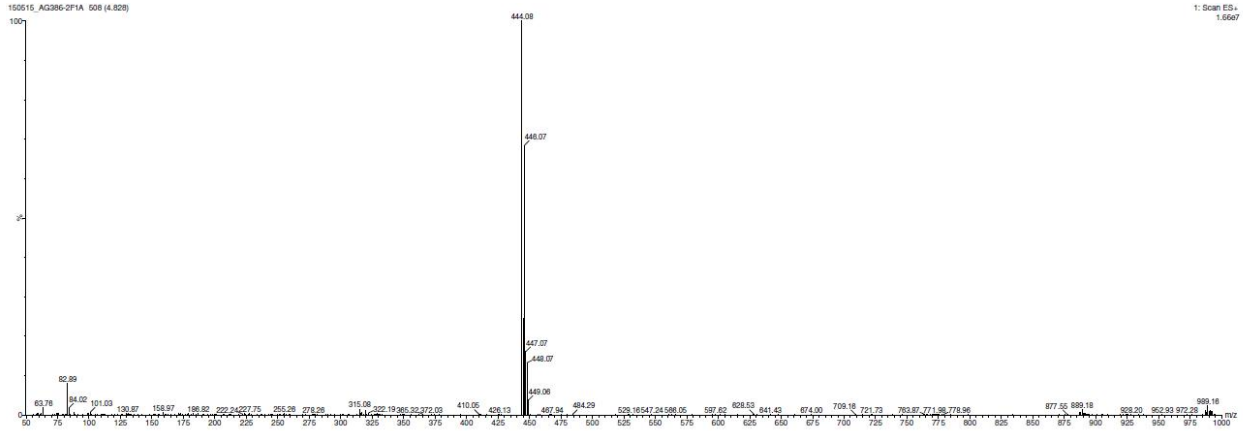

Figure S31. LC-MS chromatograms of compound 33 (97.60%).

Characterizations of compound 34.

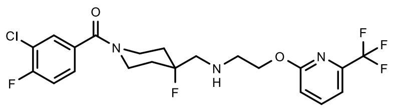

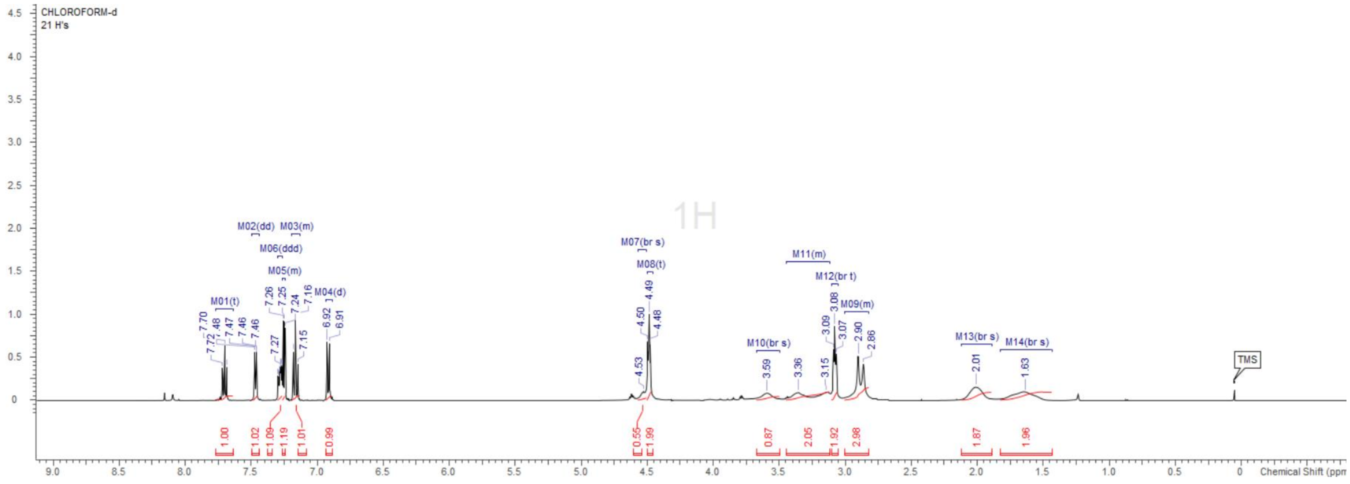

Figure S32. ^{1}H NMR (500 MHz, $CDCl_3$) spectrum of compound 34.

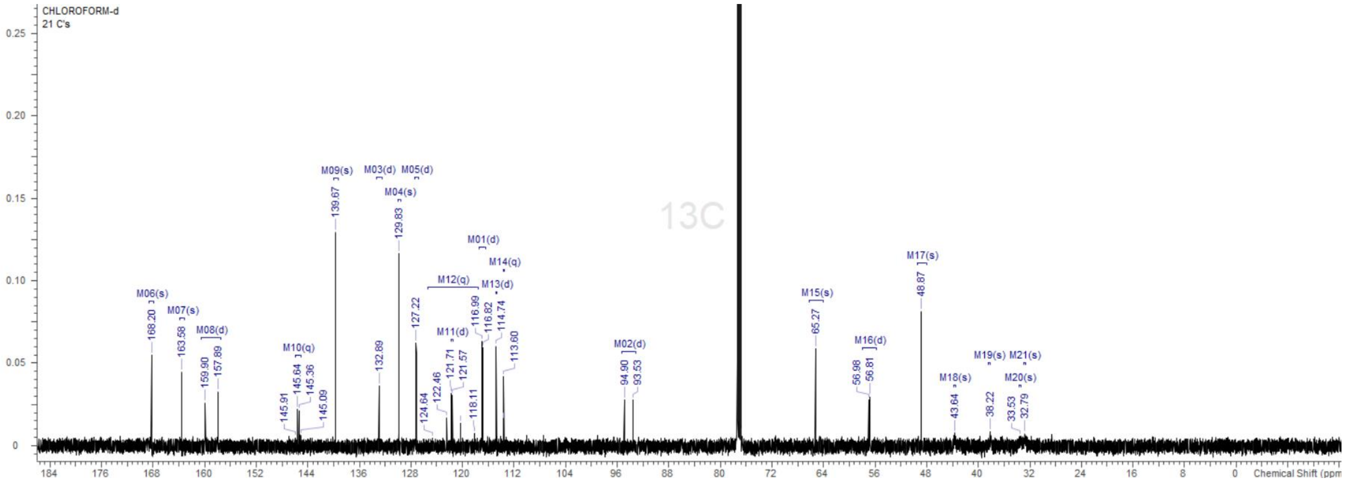

Figure S33. ^{13}C NMR (126 MHz, $CDCl_3$) spectrum of compound 34.

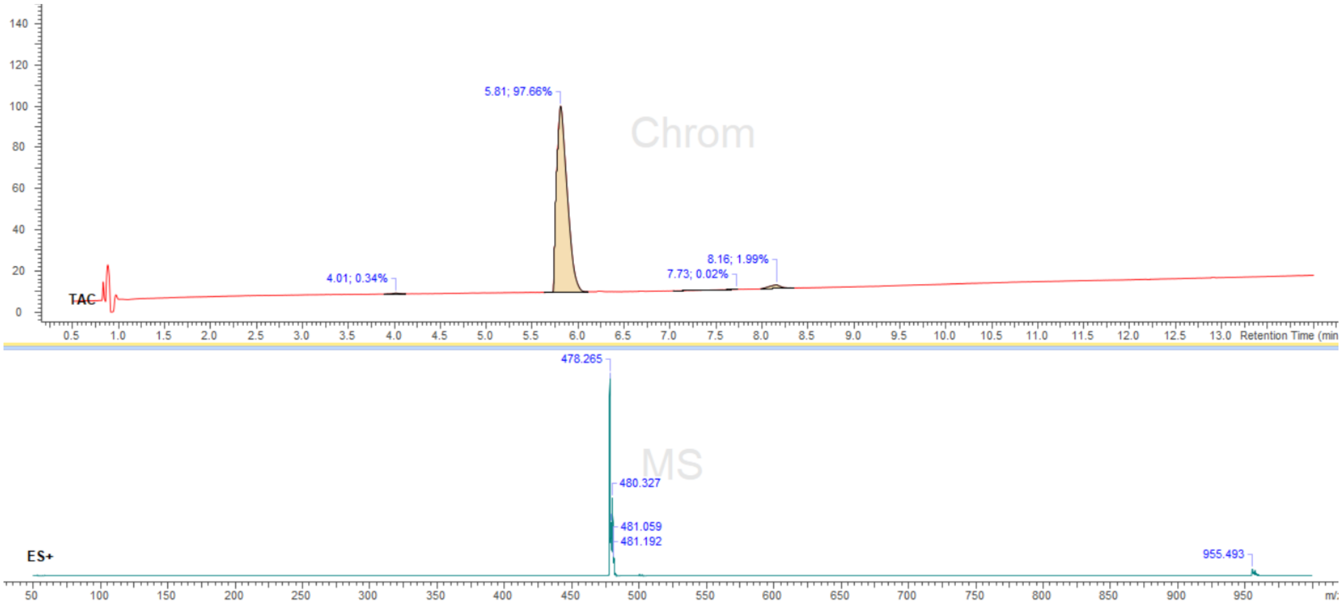

Figure S34. LC-MS chromatograms of compound 34 (97.66%).

Characterizations of compound 35.

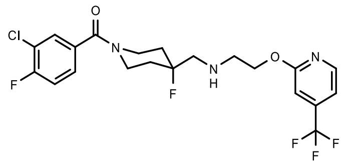

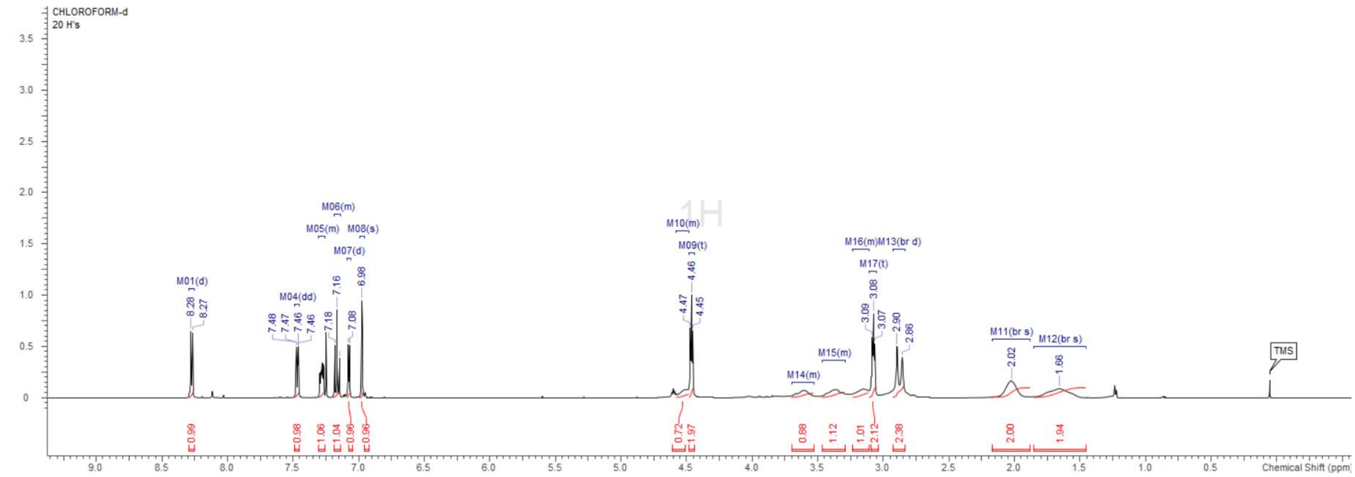

Figure S35. ^{1}H NMR (500 MHz, $CDCl_3$) spectrum of compound 35.

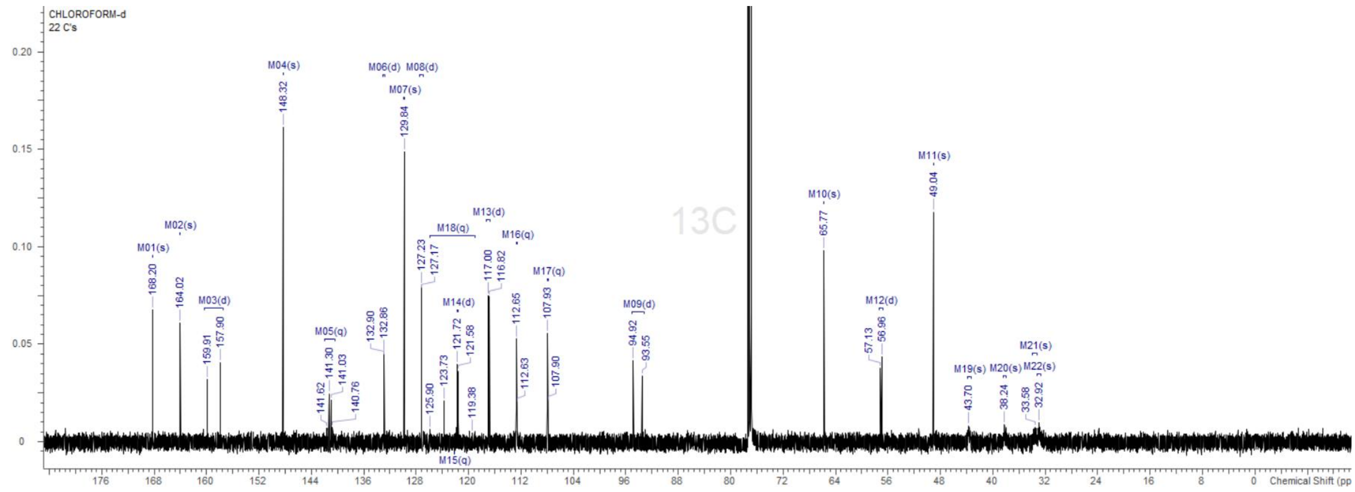

Figure S36. ^{13}C NMR (126 MHz, $CDCl_3$) spectrum of compound 35.

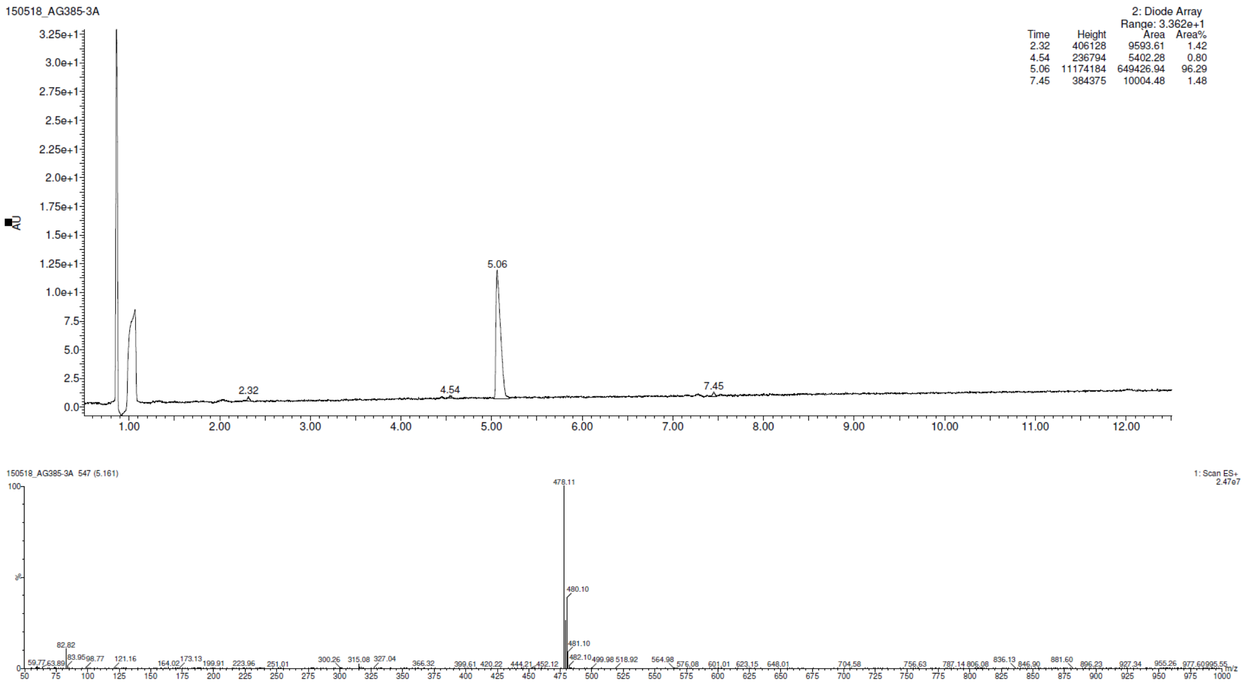

Figure S37. LC-MS chromatograms of compound 35 (96.29%).

Characterizations of compound 36.

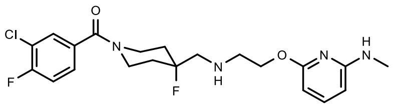

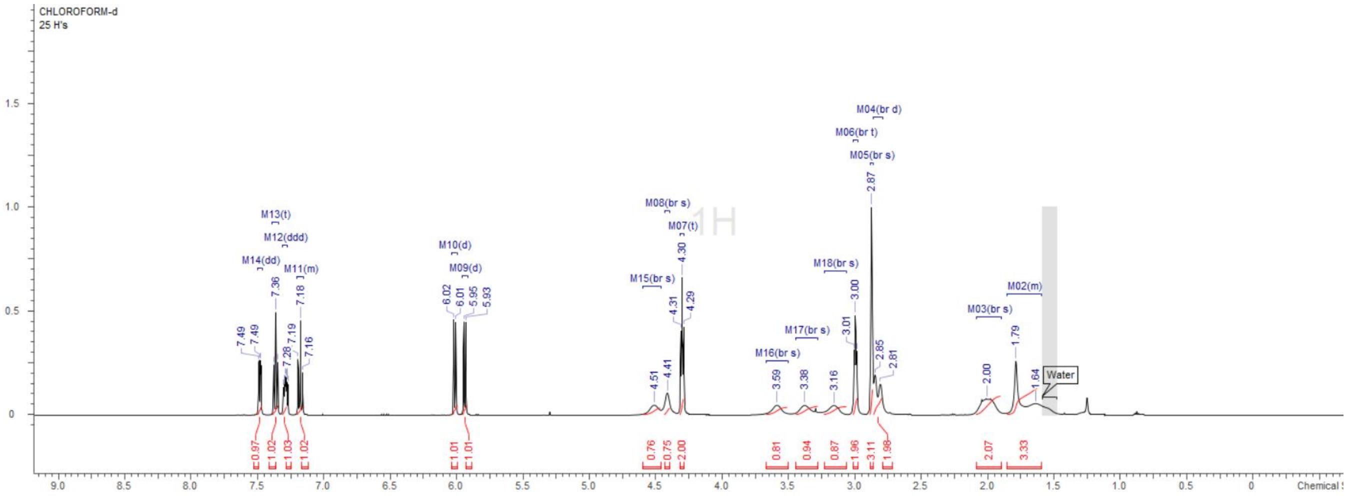

Figure S38. ^{1}H NMR (500 MHz, $CDCl_3$) spectrum of compound 36.

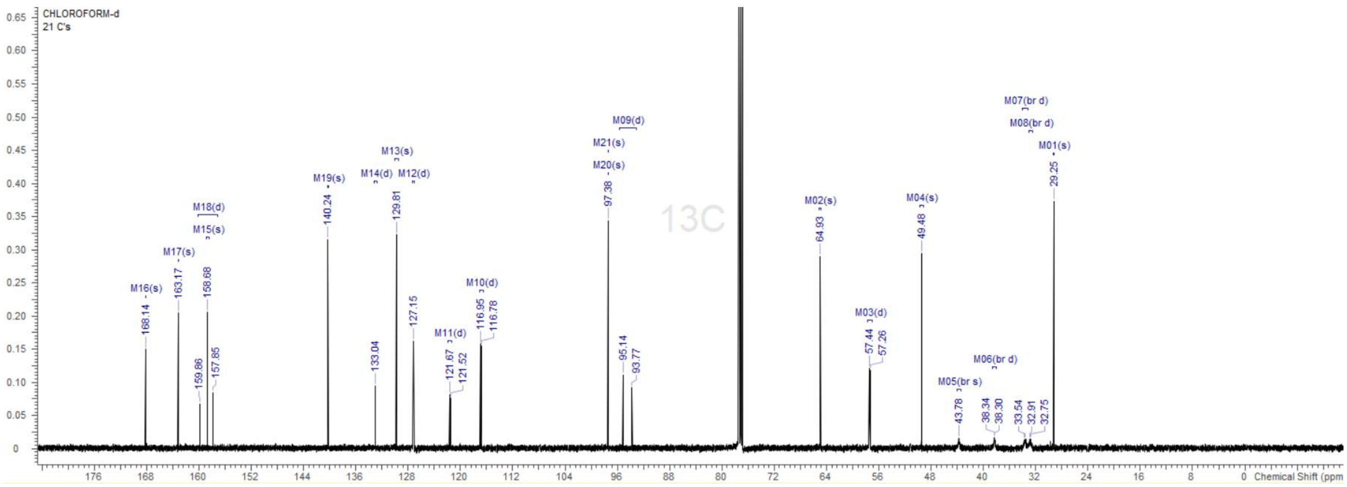

Figure S39. ^{13}C NMR (126 MHz, $CDCl_3$) spectrum of compound 36.

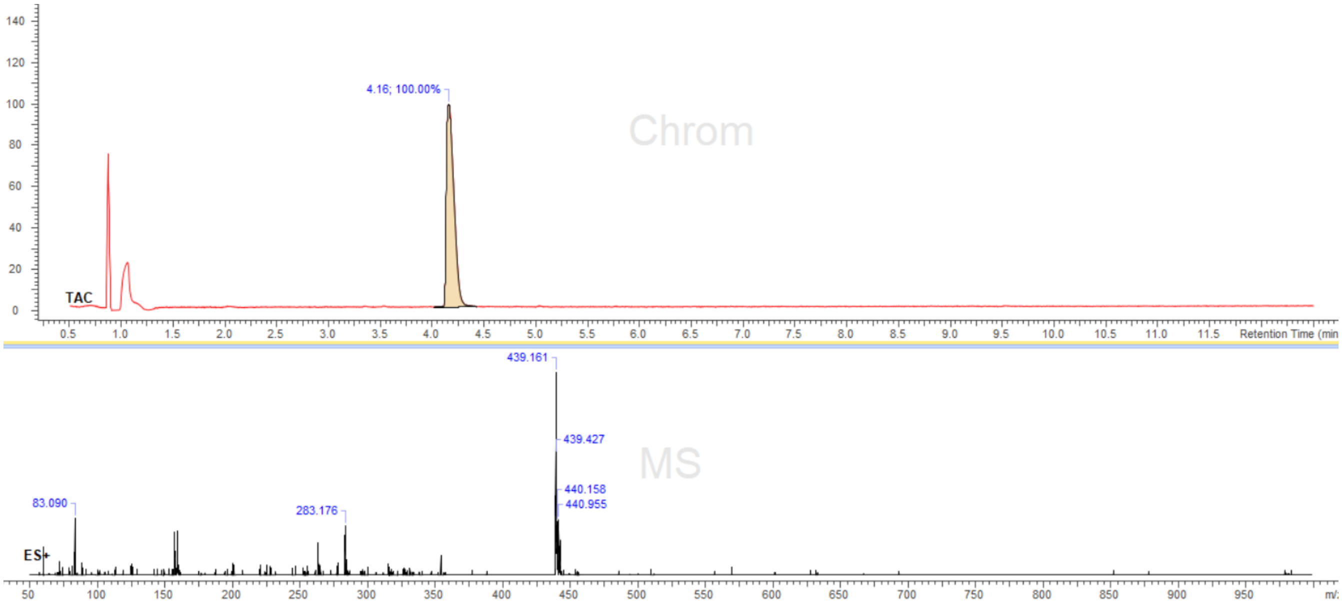

Figure S40. LC-MS chromatograms of compound 36 (100.00%).

Characterizations of compound 37.

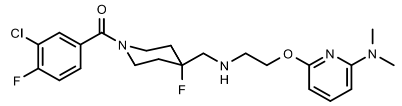

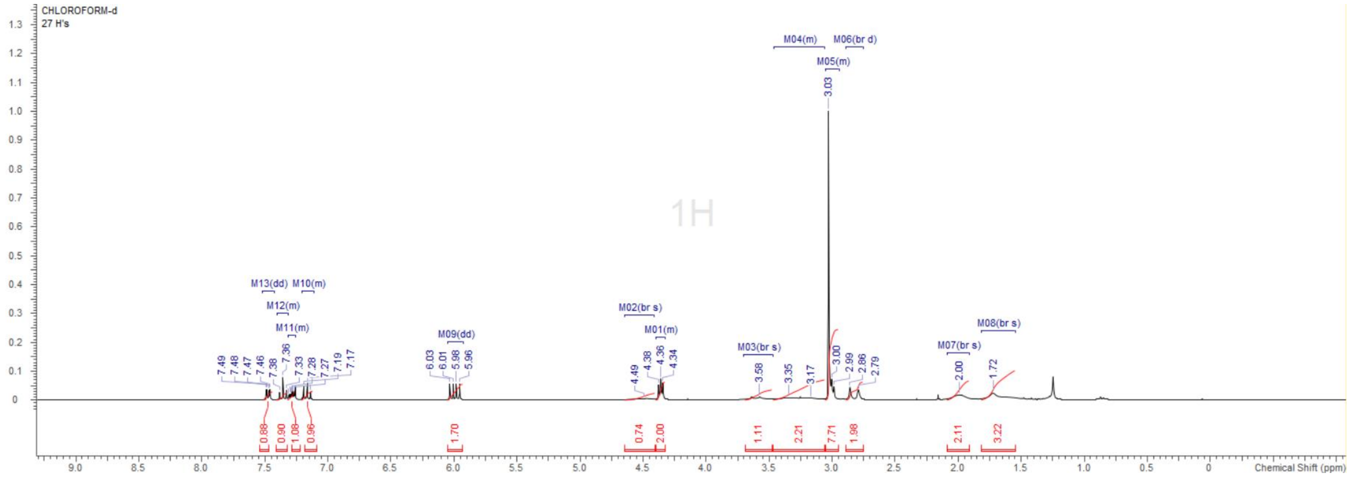

Figure S41. ^{1}H NMR (300 MHz, $CDCl_3$) spectrum of compound 37.

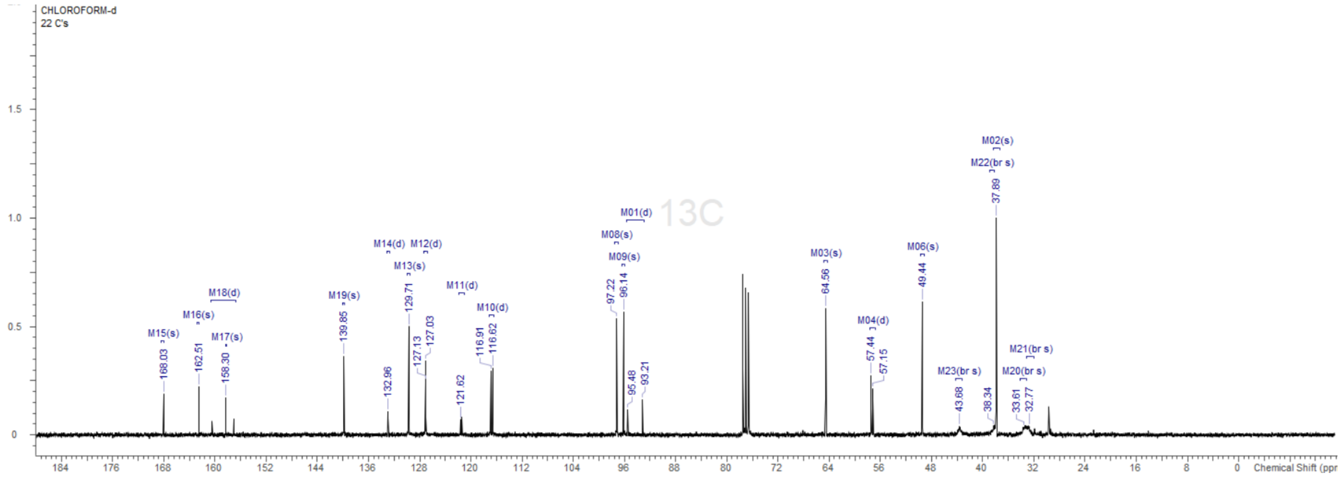

Figure S42. ^{13}C NMR (75 MHz, $CDCl_3$) spectrum of compound 37.

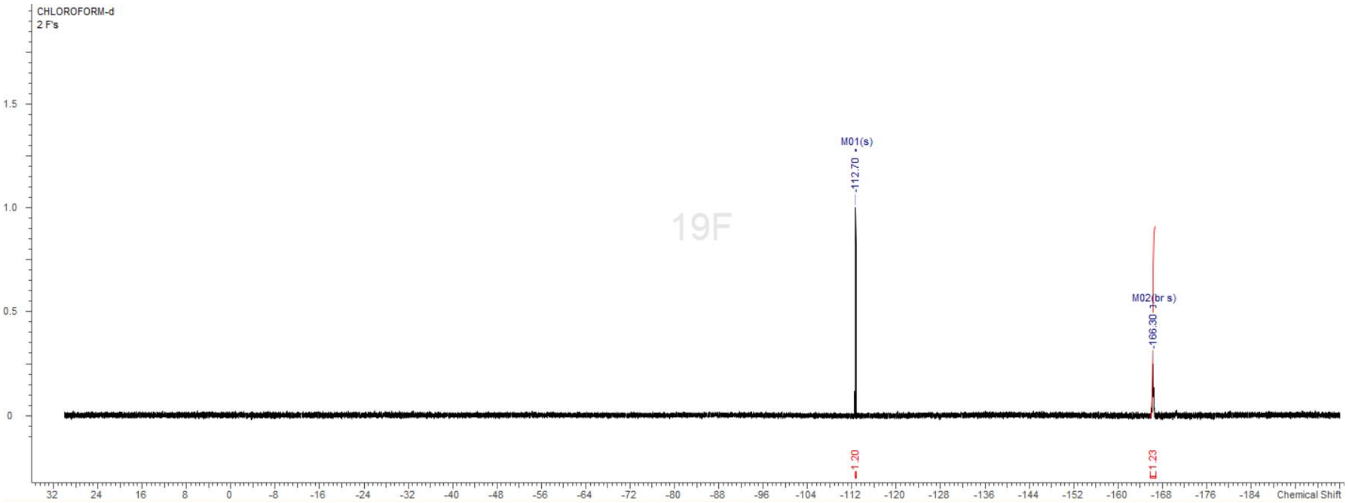

Figure S43. ^{19}F NMR (282 MHz, $CDCl_3$) spectrum of compound 37.

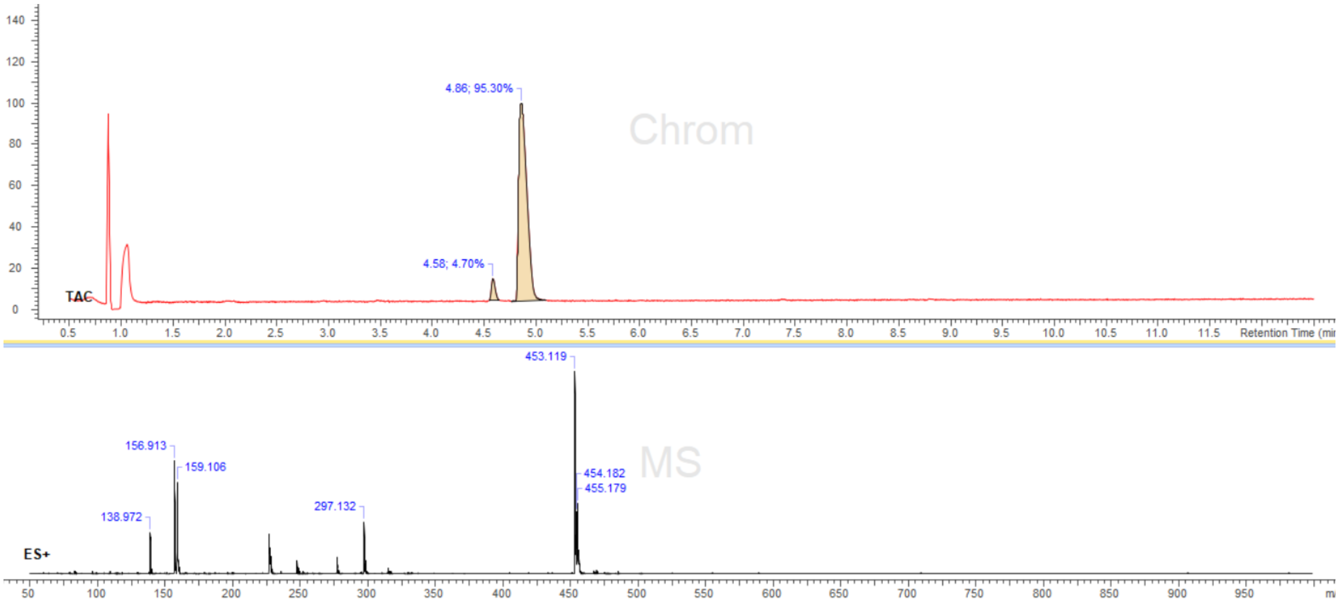

Figure S44. LC-MS chromatograms of compound 37 (95.30%).

Characterizations of compound 38.

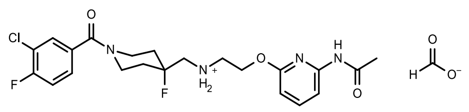

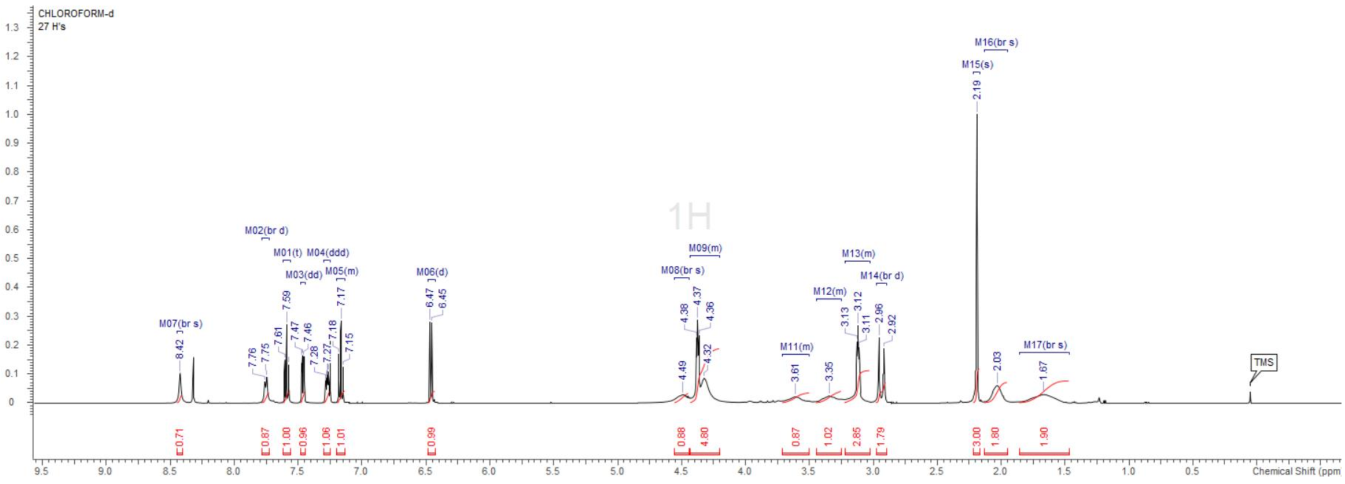

Figure S45. ^{1}H NMR (500 MHz, $CDCl_3$) spectrum of compound 38.

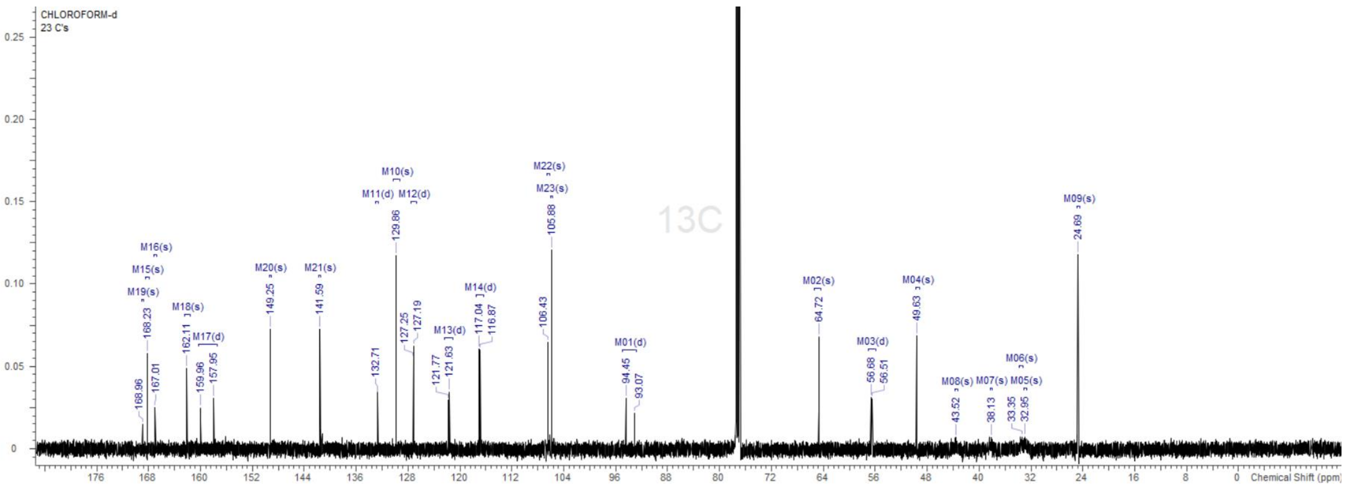

Figure S46. ^{13}C NMR (126 MHz, $CDCl_3$) spectrum of compound 38.

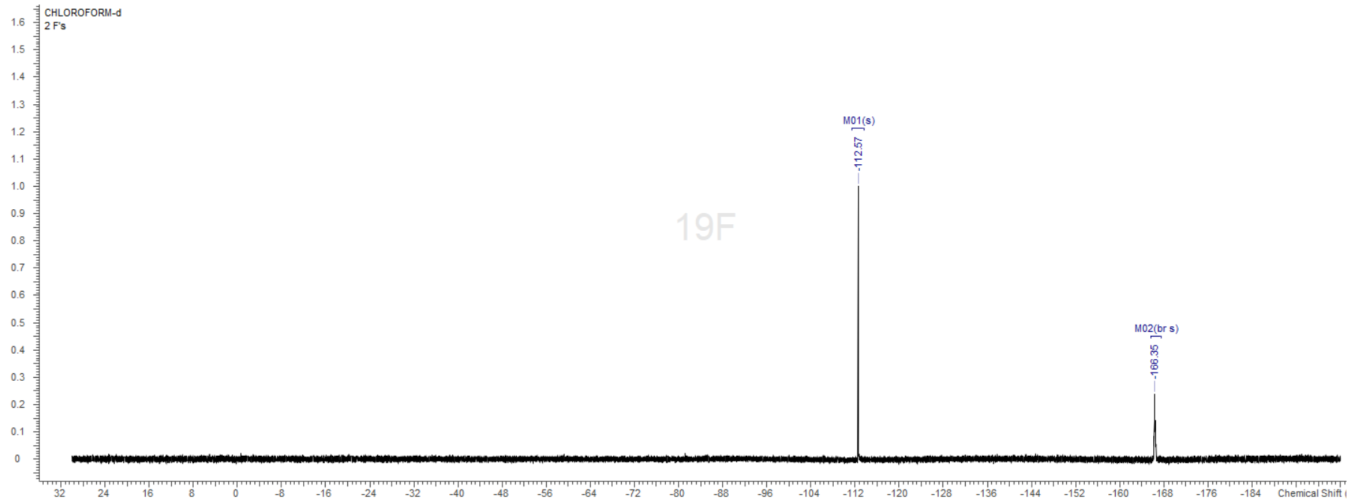

Figure S47. ^{19}F NMR (282 MHz, $CDCl_3$) spectrum of compound 38**.**

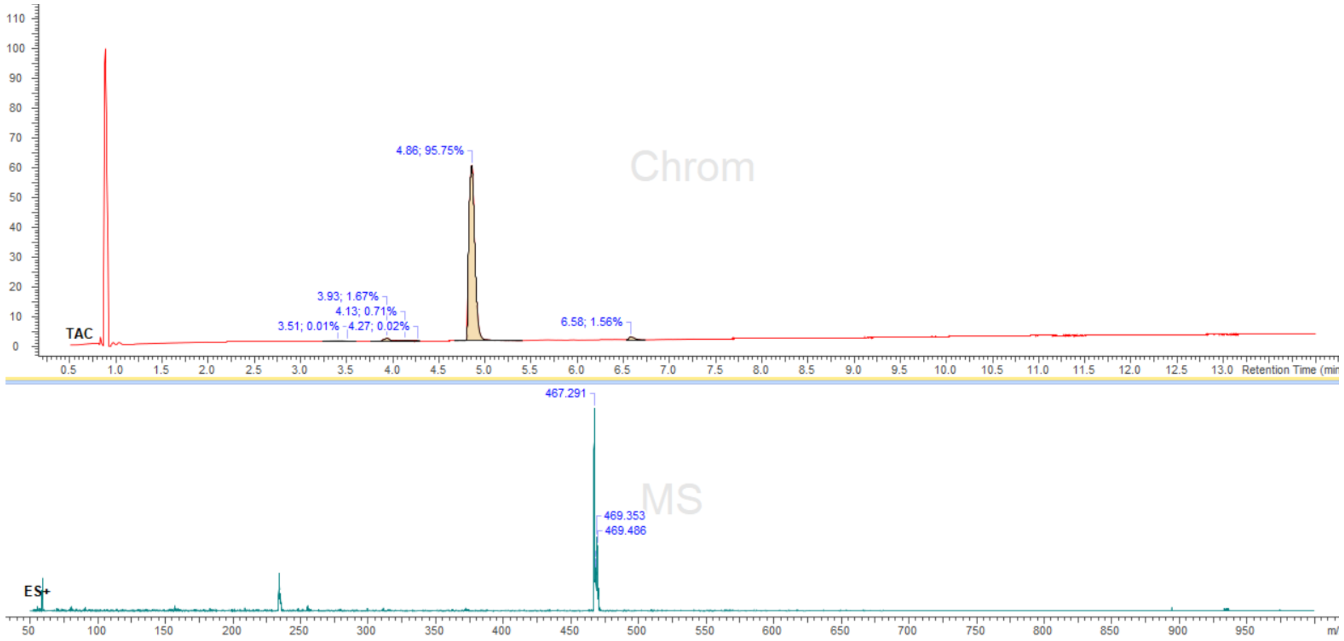

Figure S48. LC-MS chromatograms of compound 38 (95.75%).

Characterizations of compound 39.

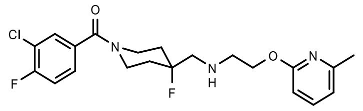

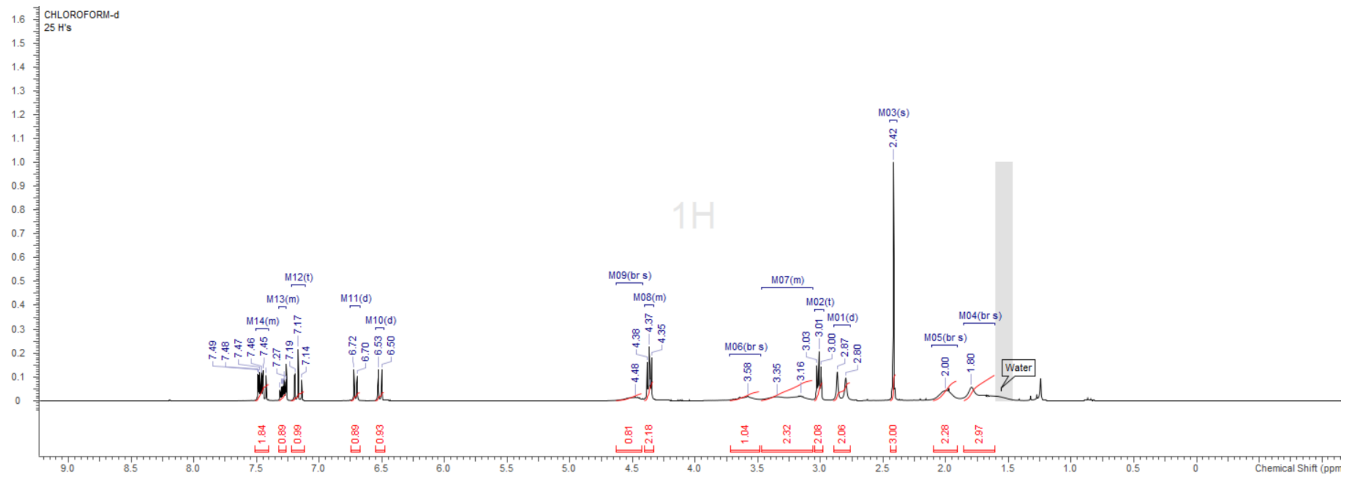

Figure S49. ^{1}H NMR (300 MHz, $CDCl_3$) spectrum of compound 39.

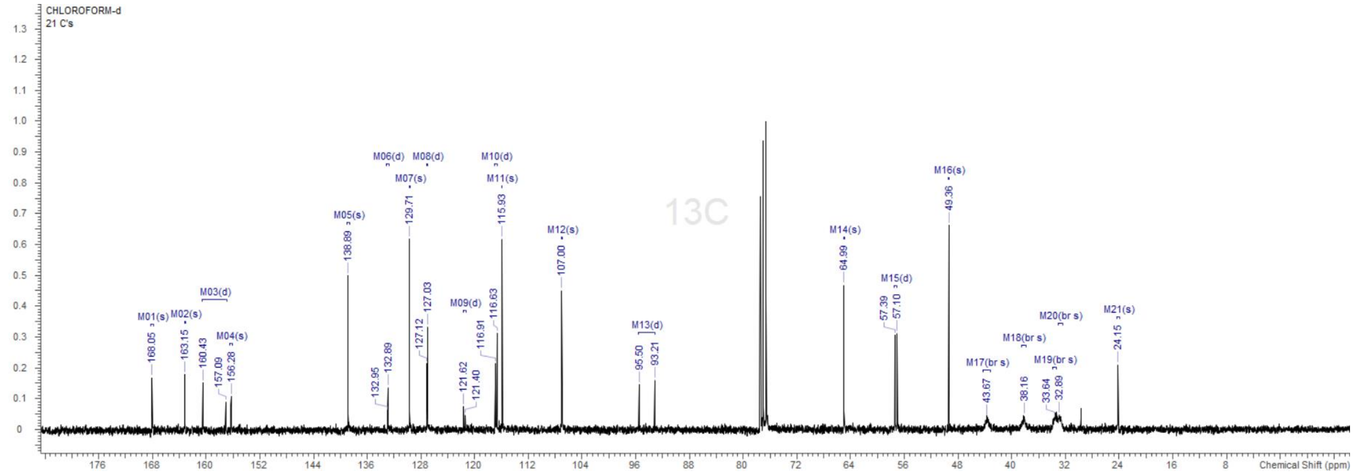

Figure S50. ^{13}C NMR (75 MHz, $CDCl_3$) spectrum of compound 39.

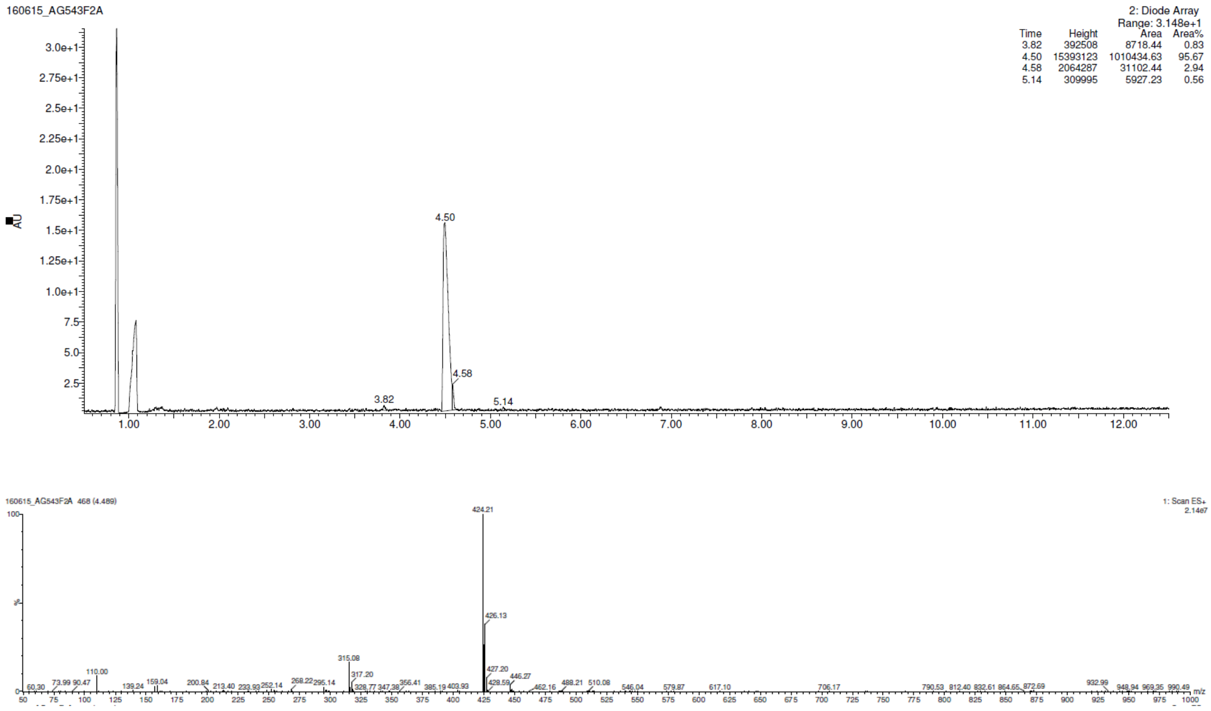

Figure S51. LC-MS chromatograms of compound 39 (95.67%).

Characterizations of compound 40 was described previously by Sniecikowska et al. JMC 2019.

Characterizations of compound 41.

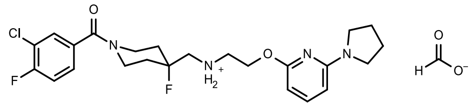

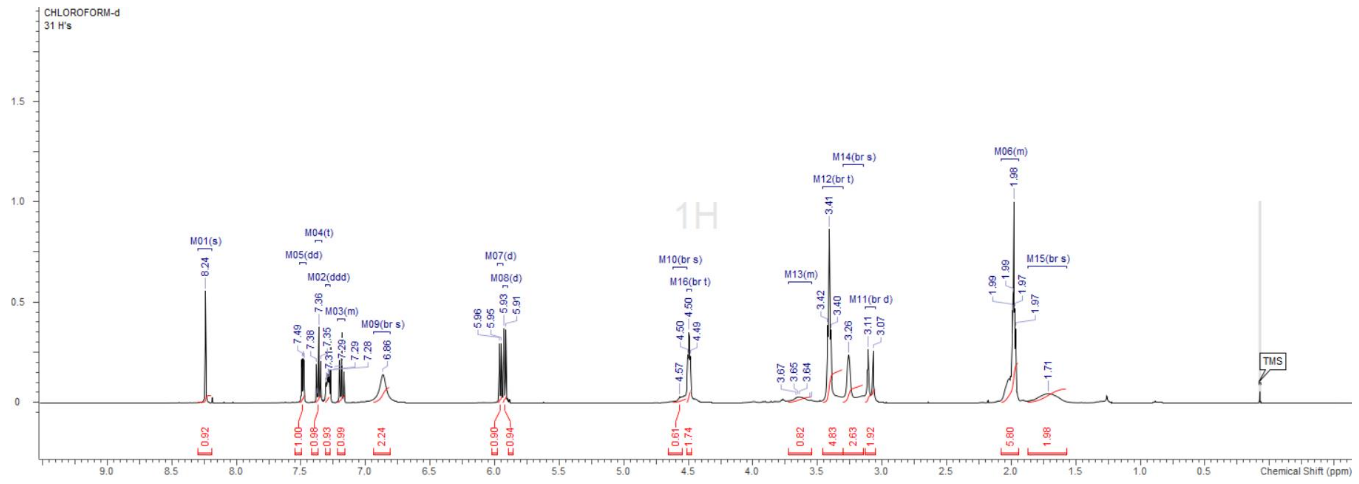

Figure S52. ^{1}H NMR (500 MHz, $CDCl_3$) spectrum of compound 41.

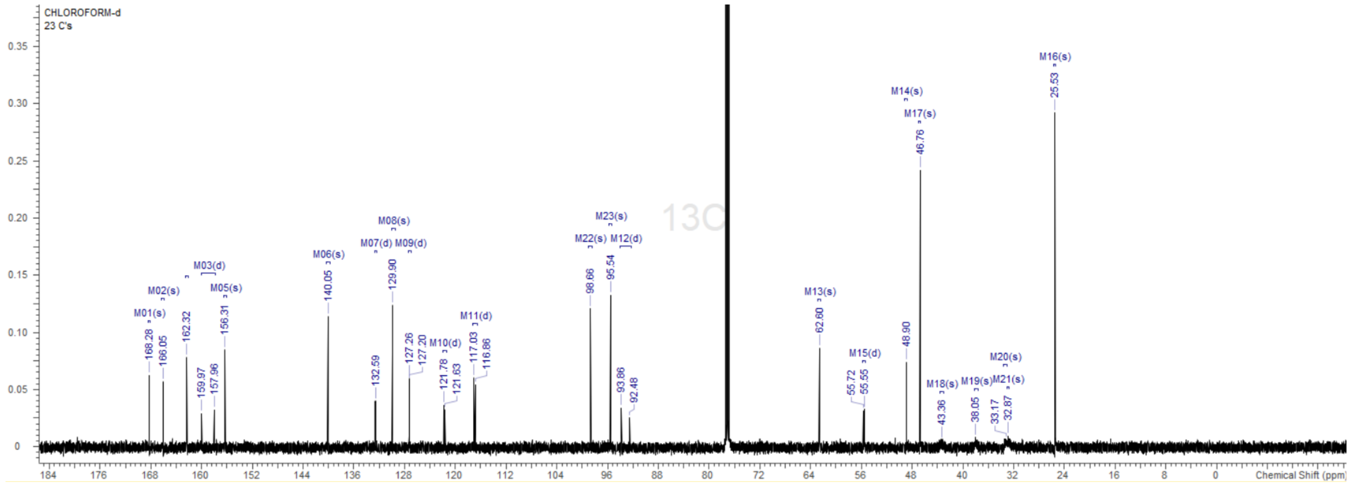

Figure S53. ^{13}C NMR (126 MHz, $CDCl_3$) spectrum of compound 41.

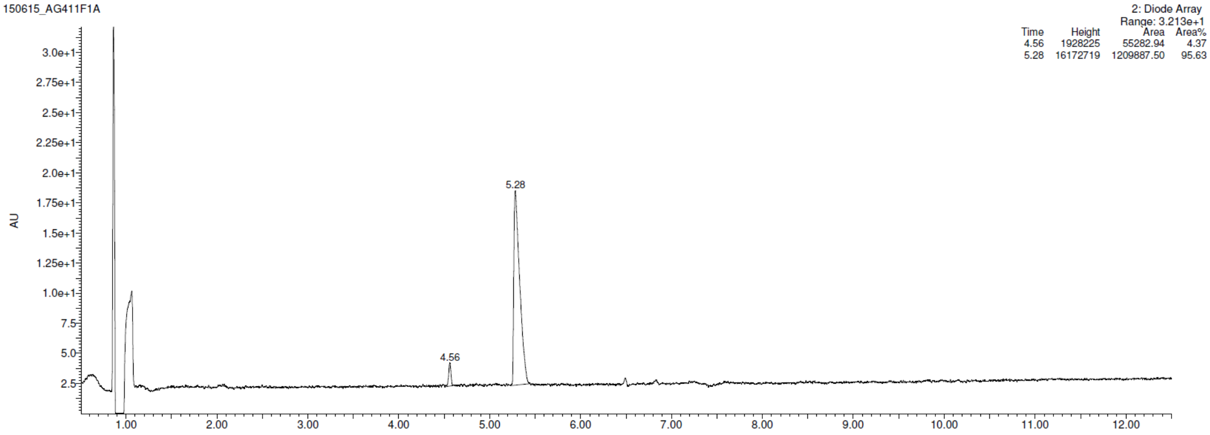

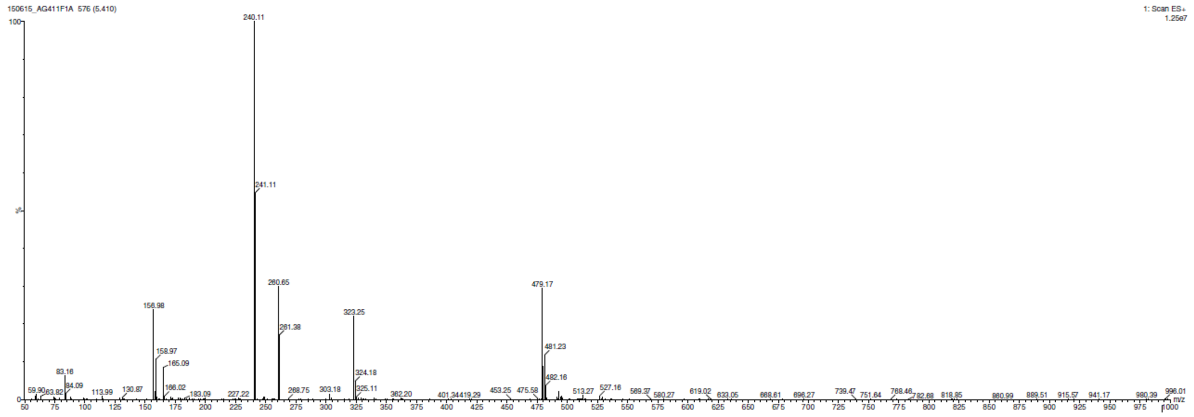

Figure S54. LC-MS chromatograms of compound 41 (95.63%).

Characterizations of compound 42.

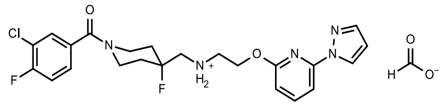

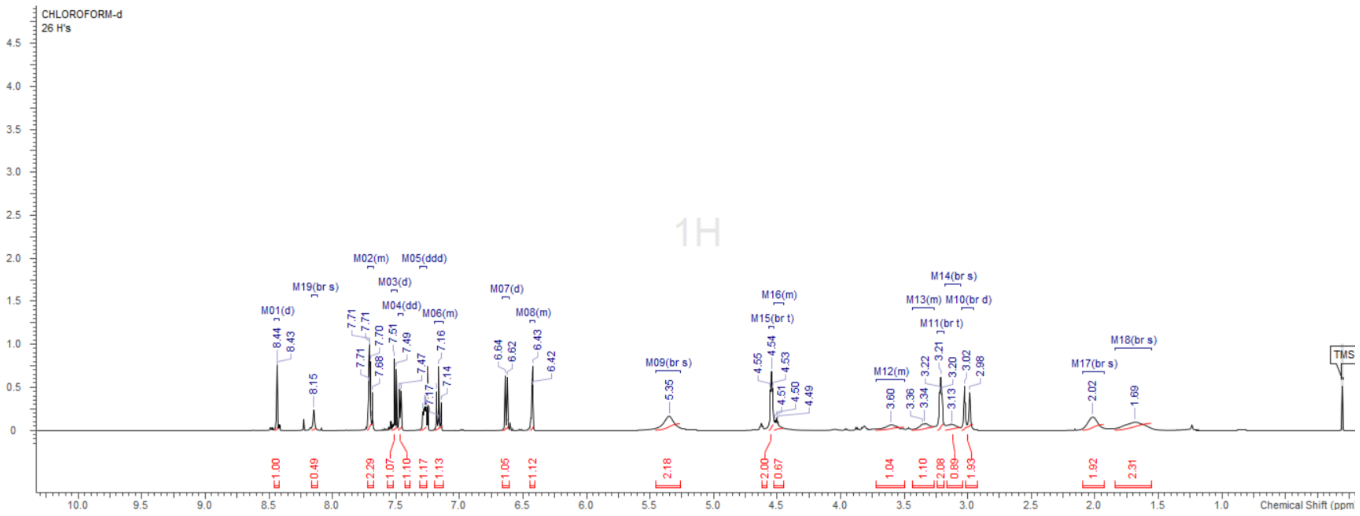

Figure S55. ^{1}H NMR (500 MHz, $CDCl_3$) spectrum of compound 42.

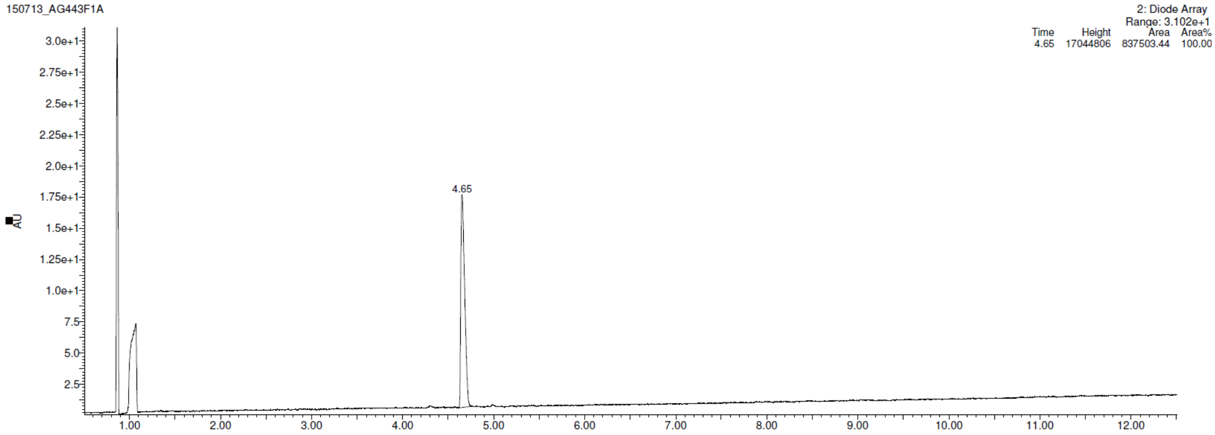

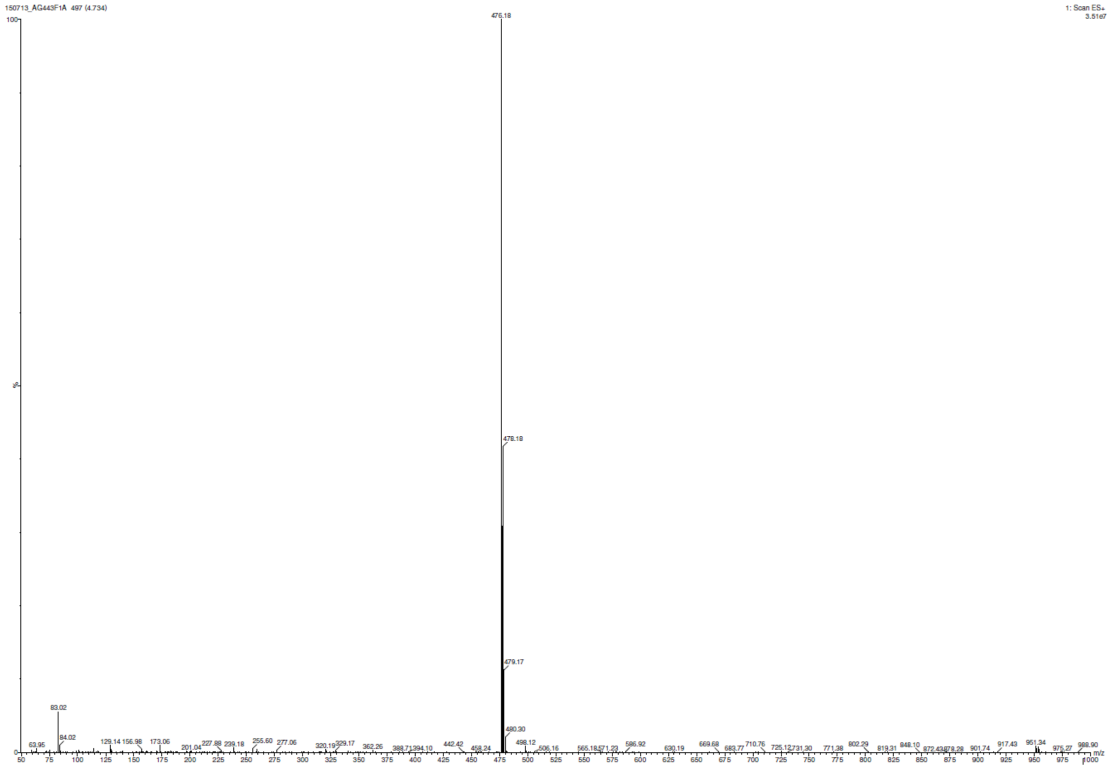

Figure S56. LC-MS chromatograms of compound 42 (100.00%).

5. **HPLC traces of the final compounds.**

Compound 25 (97.64%).

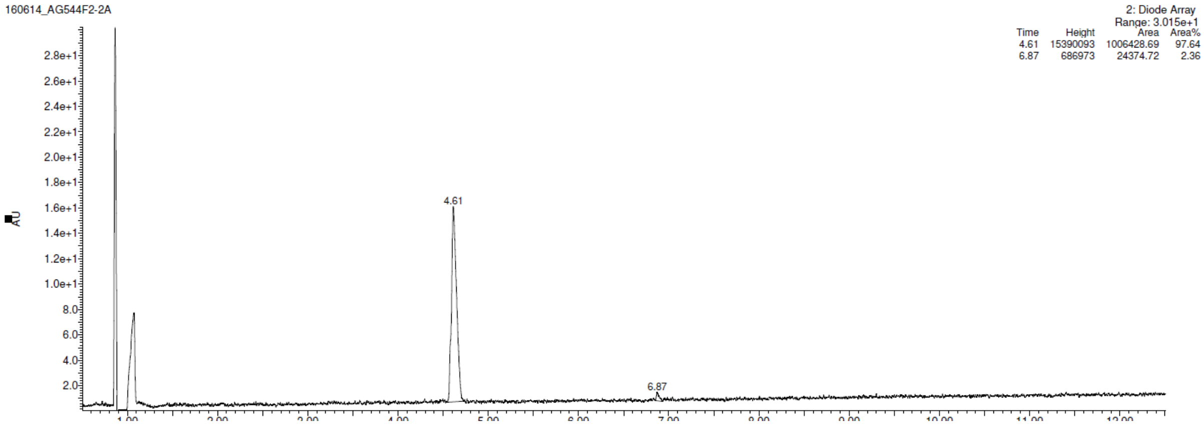

Compound 26 (100.00%)

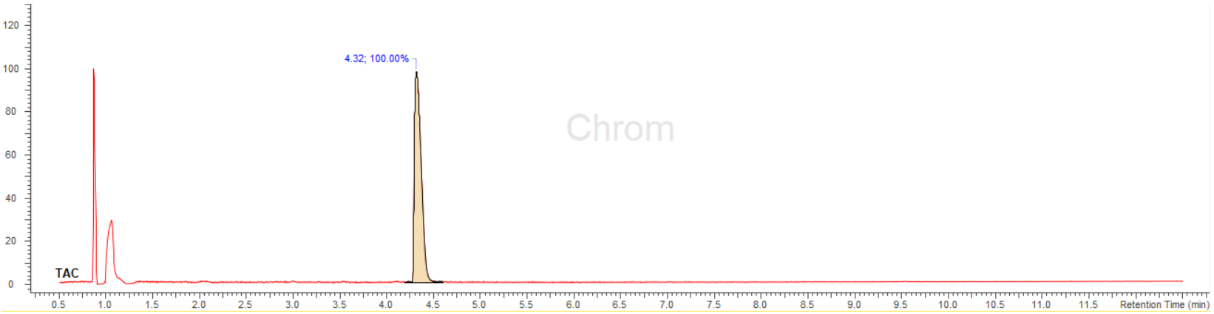

Compound 27 (95.80%)

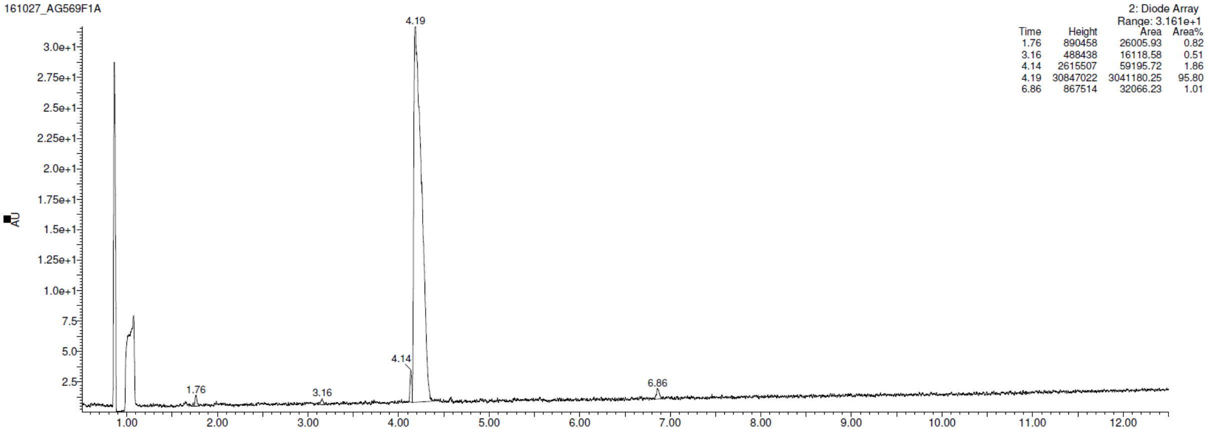

Compound 28 (95.85%)

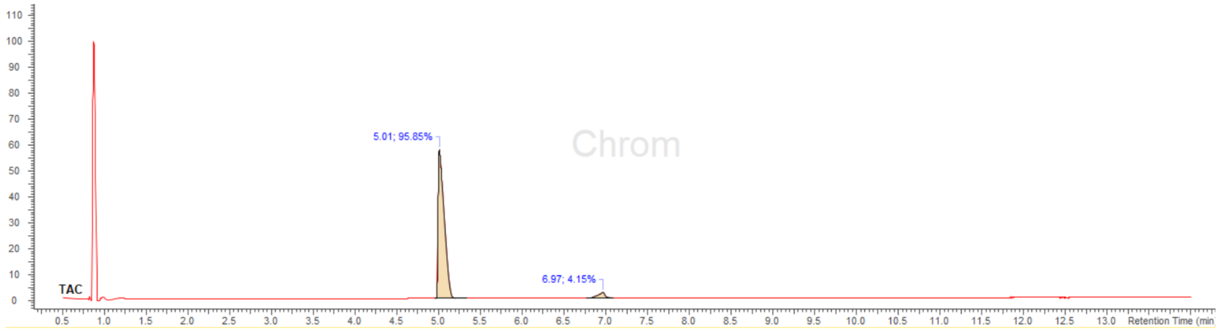

Compound 29 (97,09%)

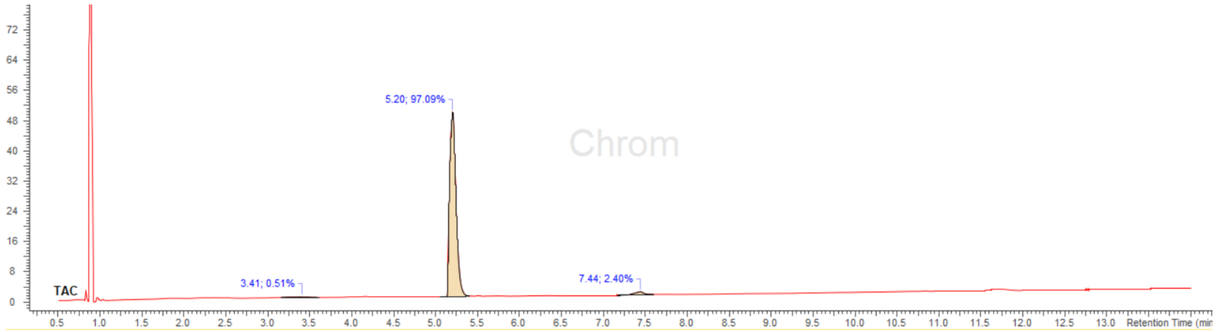

Compound 30 (96,53%)

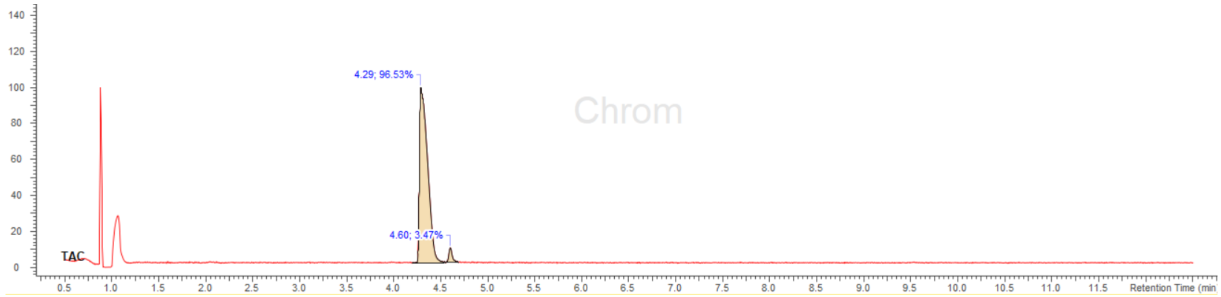

Compound 31 (100.00%)

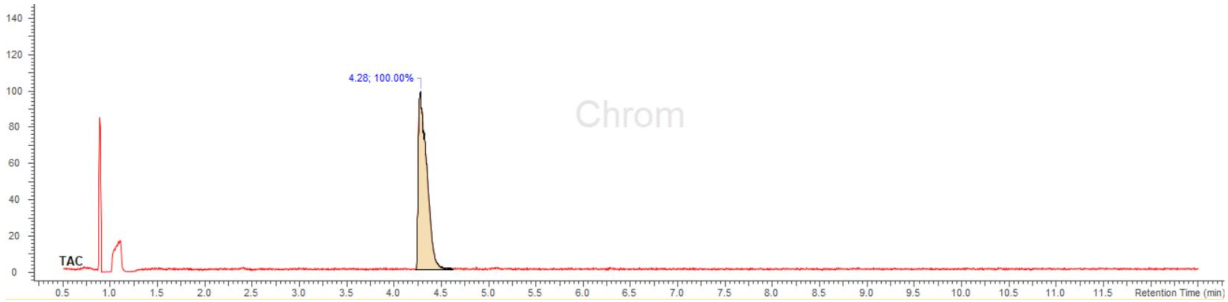

Compound 32 (99.68%)

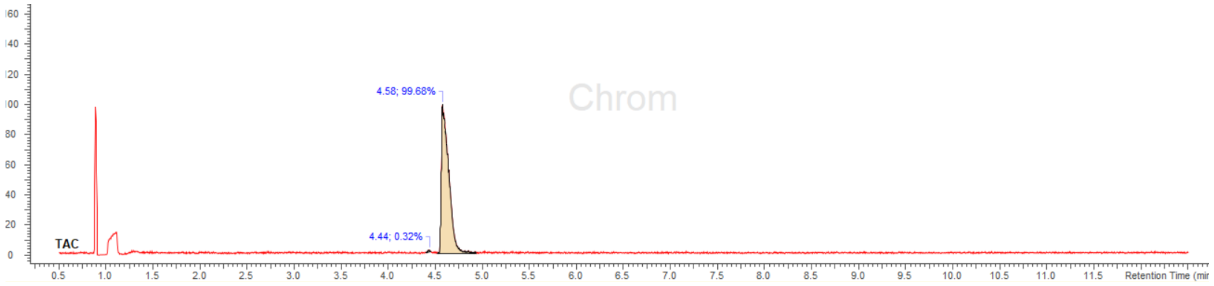

Compound 33 (97.60%)

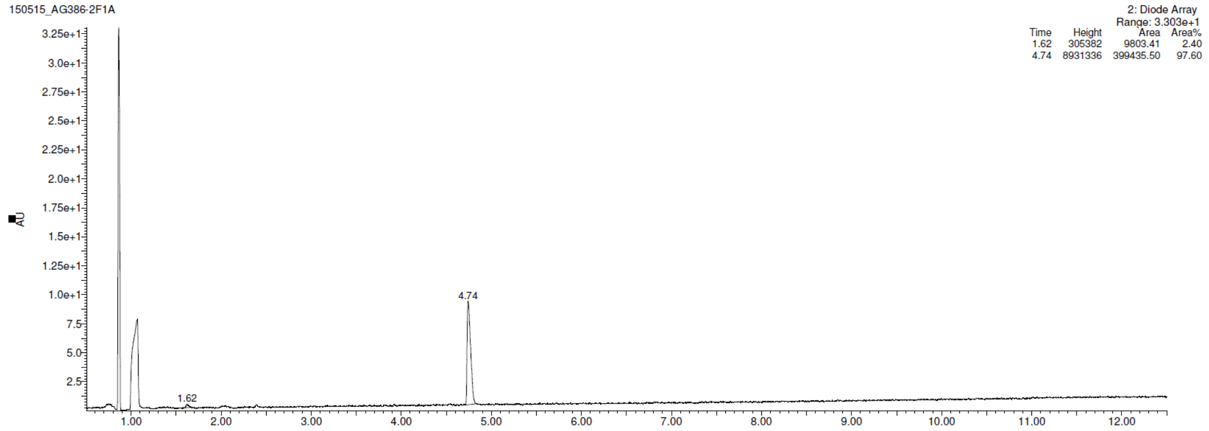

Compound 34 (97.66%)

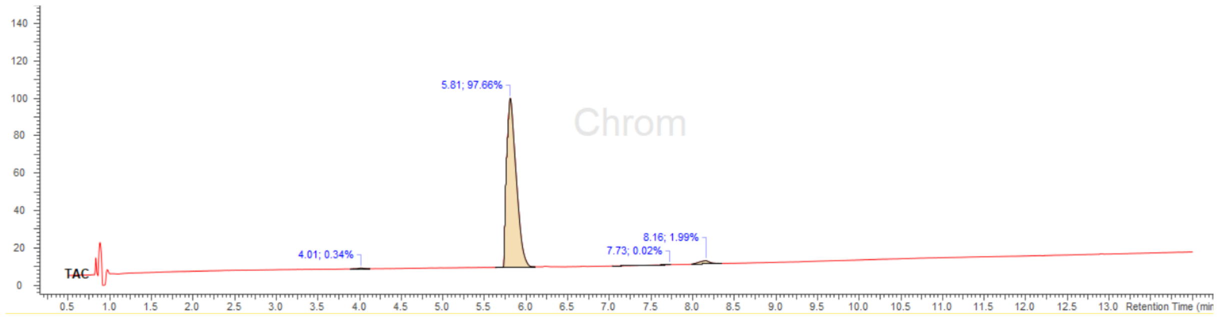

Compound 35 (96.29%)

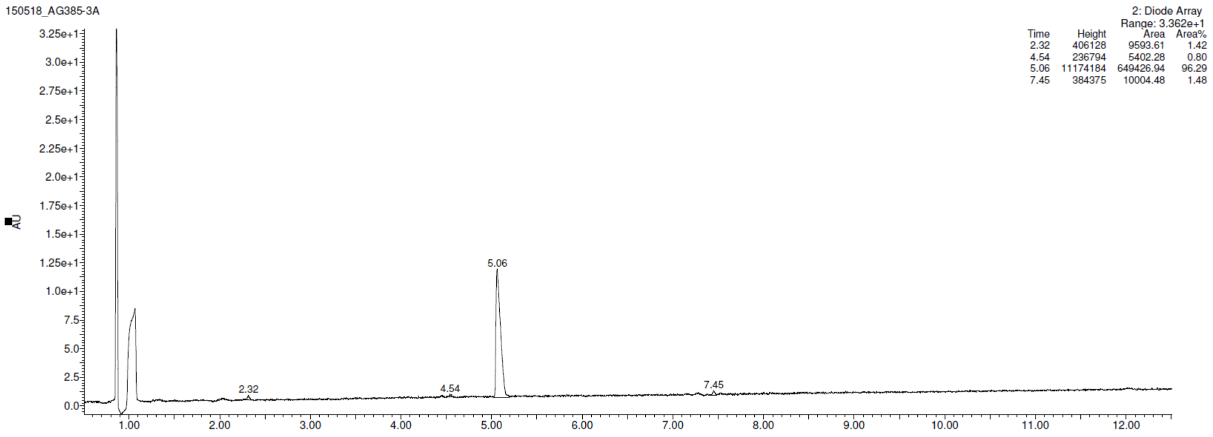

Compound 36 (100.00%)

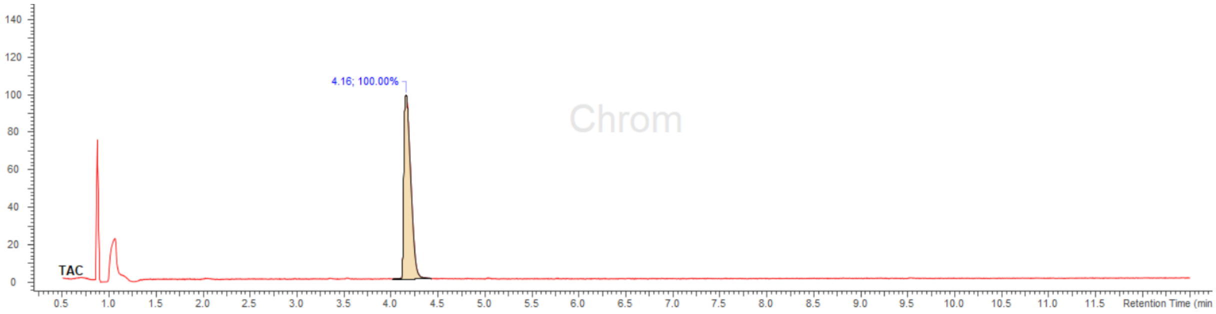

Compound 37 (95.30%)

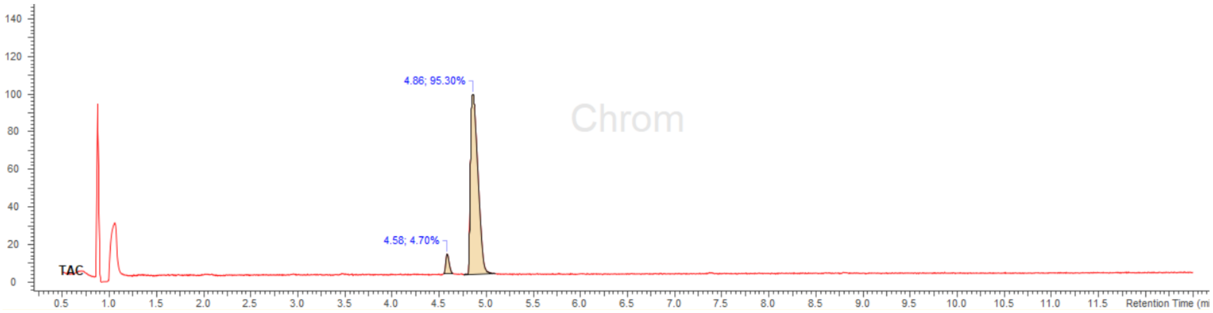

Compound 38 (95.75%)

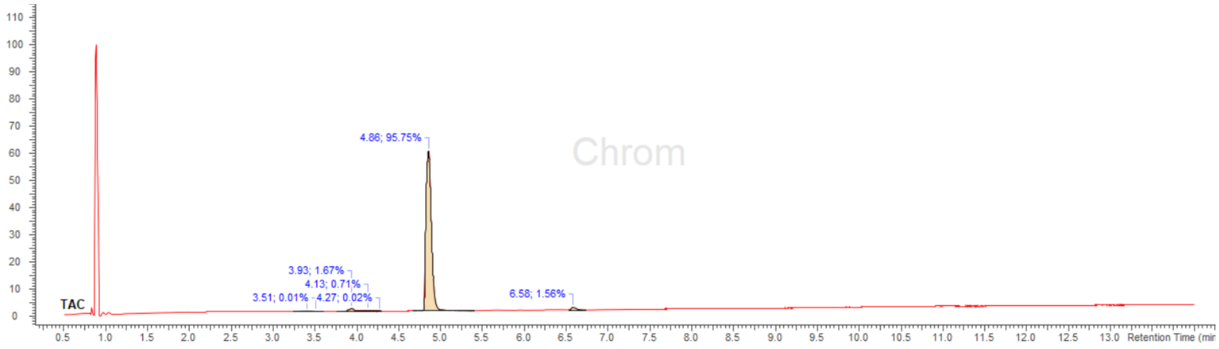

Compound 39 (95.67%)

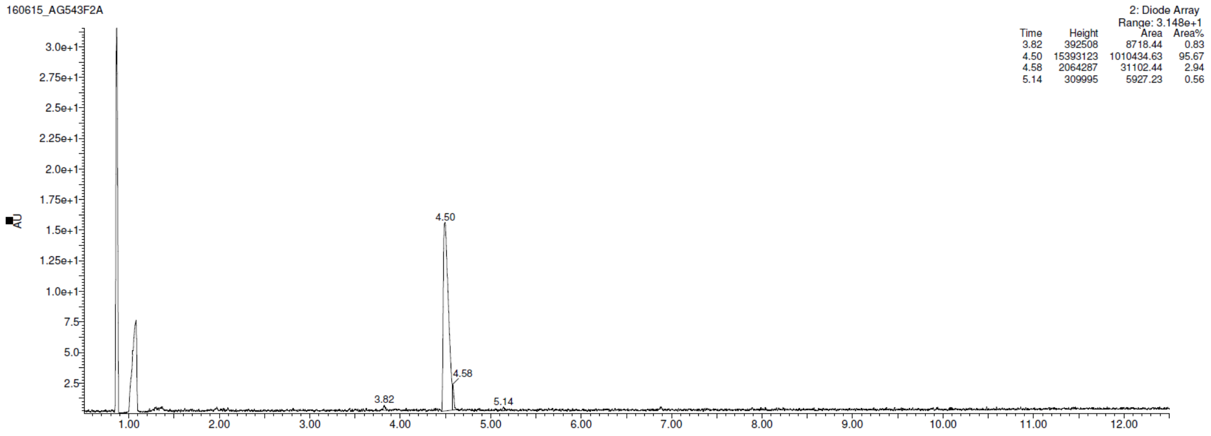

Compound 40 was described previously by Sniecikowska et al. JMC 2019.

Compound 41 (95.63%)

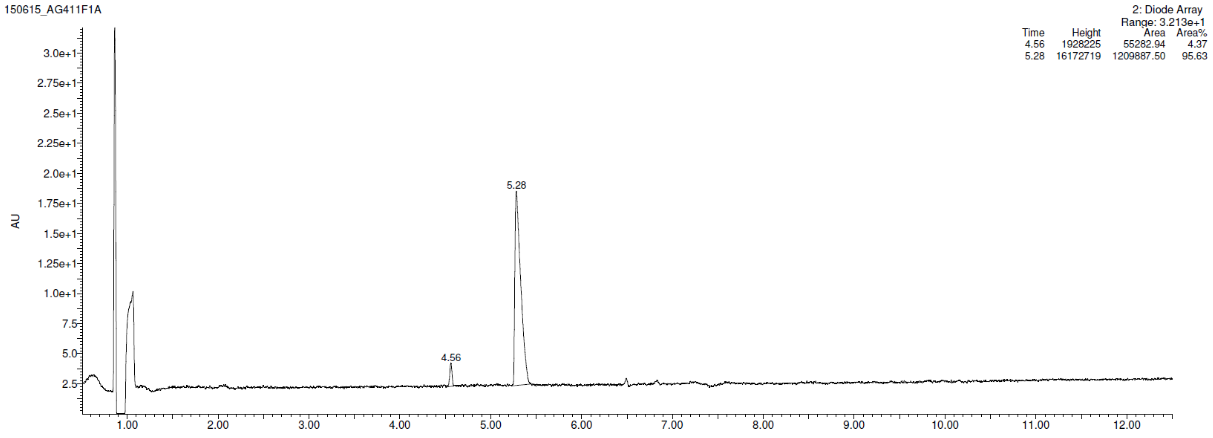

Compound 42 (100.00%)

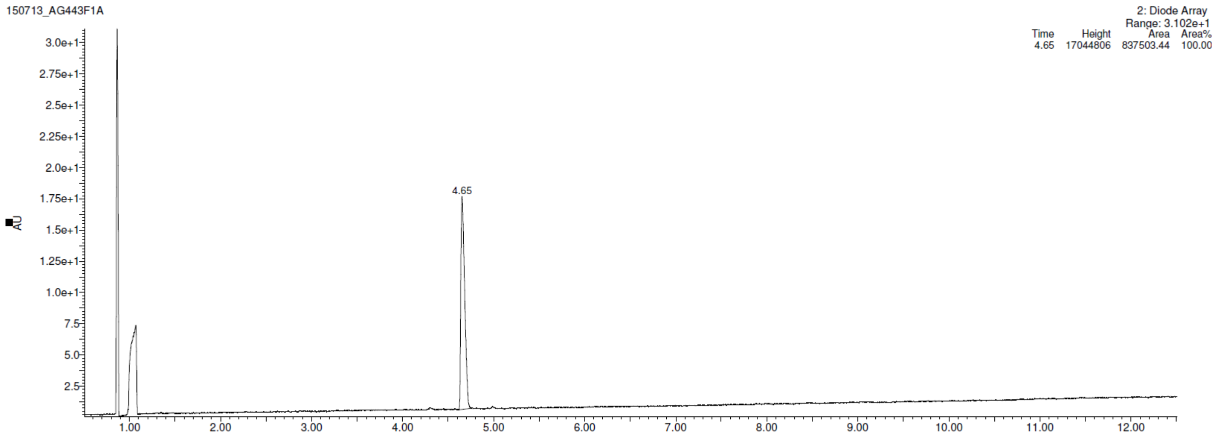

6. Optimized settings for quantitative analysis of the investigated compounds in the *In Vivo* Pharmacokinetic Studies

Table S7. Optimized settings for quantitative analysis of the investigated compounds.

Compound	Rt [min]	Transition	Cone potential [V]	Collision energy [eV]
27	3.76	440.1 → 126.0	38	16
		440.1 → 157.0		42
31	3.78	428.1 → 156.9	16	36
		428.1 → 315.1		12

7. References

(1) Juan, B. V.; Trias, C. E. New-4-(Pyrrolopyrimidin-6-Yl)Benzenesulphonamide Derivatives. EP1492793B1, January 16, 2008. https://patents.google.com/patent/EP1492793B1/en (accessed 2024-08-23).

(2) Sniecikowska, J.; Bucki, A.; Newman-Tancredi, A.; Varney, M. A. Compounds for Treating Disorders Sensitive to Serotoninergic Regulation Controlled by the 5-Ht1a Receptors. US2019194132 (A1), June 27, 2019. https://worldwide.espacenet.com/publicationDetails/biblio?FT=D&date=20190627&DB=&locale=en_EP&CC=US&NR=2019194132A1&KC=A1&ND=4 (accessed 2024-08-23).

(3) Espacenet – search results. https://worldwide.espacenet.com/patent/search/family/085897936/publication/CN115960032A?q=pn%3DCN115960032A (accessed 2023-07-20).

(4) Fernandez, M. C.; Gonzalez-Garcia, M. R.; Pfeifer, L. A. Benzyl Sulfonamide Derivatives Useful as Mogat - 2 Inhibitors. CA2859995A1, August 8, 2013.

(5) Harvey, A.; Bombrun, A.; Cooke, R.; Jeanclaude-Etter, I.; Kuchel, N.; Molette, J.; Mould, J.; Paul, D.; Singh, R.; Donini, C.; Colovray, V.; Avery, T.; Crossman, J.; Ripper, J. Amine Derivatives as Potassium Channel Blockers. US2018273476A1, September 27, 2018.

(6) Zielinski, J. Certain Substituted Aminothioethoxy Pyridines. US3535328A, October 20, 1970.

(7) Greul, J. N.; Schwarz, H.-G.; Alig, B.; Becker, A.; Portz, D.; Ilg, K.; Gorgens, U.; WELZ, C. Pyridyloxyalkyl Carboxamides and Use Thereof as Endoparasiticides and Nematicides. CA2890826A1, May 22, 2014. https://patents.google.com/patent/CA2890826A1/en (accessed 2024-08-23).

(8) Yamagishi, T.; Morita, M.; Shishido, Y.; Yamaguchi, R.; Gaja, N. Amide Derivatives as Nav1.7 and Nav1.8 Blockers. TWI769266B, July 1, 2022.
